# Theoretical Magnetic Relaxation and Spin–Phonon Coupling Study in a Series of Molecular Engineering Designed Bridged Dysprosocenium Analogues

**DOI:** 10.1021/acs.inorgchem.3c02916

**Published:** 2023-10-09

**Authors:** Kamil Kotrle, Mihail Atanasov, Frank Neese, Radovan Herchel

**Affiliations:** † Department of Inorganic Chemistry, Faculty of Science, Palacký University Olomouc, Olomouc CZ-77146, Czech Republic; ‡ Max-Planck-Institut für Kohlenforschung, Mülheim an der Ruhr D-45470, Germany; § Institute of General and Inorganic Chemistry, Bulgarian Academy of Sciences, Sofia 1113, Bulgaria

## Abstract

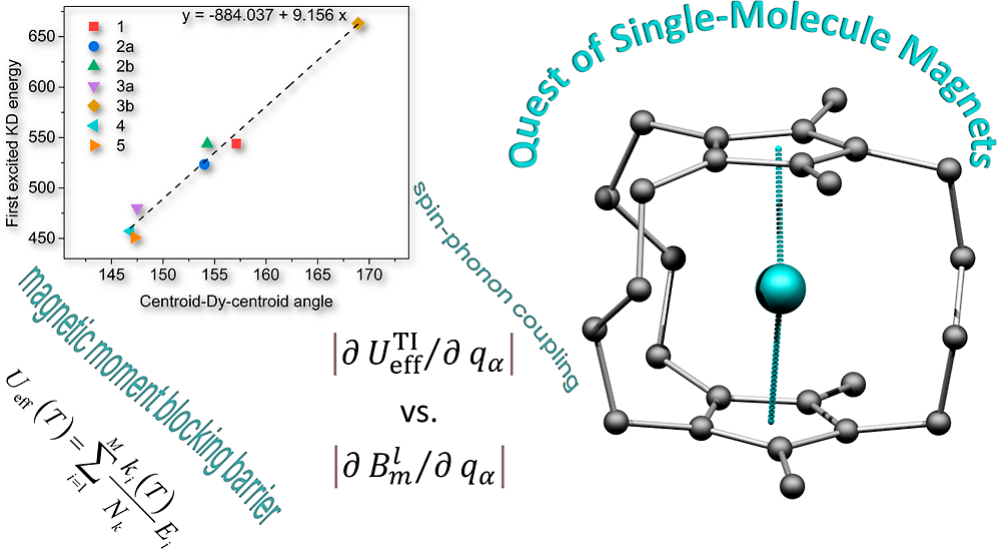

A detailed computational study of hypothetical sandwich dysprosium double-decker complexes, bridged by various numbers of aliphatic linkers, was performed to evaluate the effect of the structural modifications on their ground-state magnetic sublevels and assess their potential as candidates for single-molecule magnets (SMMs). The molecular structures of seven complexes were optimized using the TPSSh functional, and the electronic structure and magnetic properties were investigated using the complete active space self-consistent field method (CASSCF). Estimates of the magnetic moment blocking barrier (*U*_eff_) and blocking temperatures (*T*_B_) are reported. In addition, a new method based on computed derivatives of effective demagnetization barriers *U*_eff_ with respect to vibrational normal modes was introduced and applied to evaluate the impact of spin–phonon coupling on the SMM properties. On the basis of the computed parameters, we have identified promising candidates with properties superior to those of the existing single-molecule magnets.

## Abstract 2

This study explores modified dysprosium double–decker complexes with aliphatic linkers, assessing their potential as single-molecule magnets. Seven complexes were optimized and analyzed for electronic and magnetic properties. The effective demagnetization barriers (*U*_eff_) and blocking temperatures (*T*_B_) were estimated, and a novel method assessing spin–phonon coupling’s impact on SMM properties was introduced. Promising candidates surpassing current SMMs were identified based on computed parameters.

## Introduction

In recent years, research on single-molecule magnets (SMMs) has taken many remarkable steps toward increasing their blocking temperatures (*T*_B_) and relaxation times (τ). Today, the most interesting systems are unquestionably the lanthanide organometallic double-decker complexes. The increase of *T*_B_ from 20 K, in the complexes reported early on,^1^ to 60 K for [Dy(Cp^*t*Bu3^)_2_][B(C_6_F_5_)_4_] (Cp^*t*Bu3^ = 1,2,4 -tris-*t*-butylcyclopentadienyl)^2^ and to even higher temperatures, *T*_B_ = 80 K for [Dy(Cp^iPr5^)(Cp^Me5^)][B(C_6_F_5_)_4_]^3^ (Cp^iPr5^ = pentaisopropylcyclopentadienyl Cp^Me5^ = pentamethylcyclopentadienyl), paved the way for systematic increases in the performance of these remarkable SMM systems. In the search for even better compounds, new methods have been foreshadowed, like the replacement of carbon atoms in the cyclopentadienyl ring by heteroatoms^4^ or the creation of analogous [Ln^II^(Cp^iPr5^)_2_] type complexes.^5^ Although these attempts did not produce a blocking temperature of more than 80 K, the results are nonetheless promising, and additional studies can further push their limits. Sandwich complexes with different numbers of carbon atoms with remarkable magnetic properties and strong anisotropy, such as C_8_H_8_^2–^ or C_9_H_9_^–^ are also worth mentioning. Interestingly, the most promising complexes with these bigger ligands are complexes of erbium rather than dysprosium.^6^

With such progress, one question comes to mind—why is this class of complexes so much better than all the others? Their axial nature gives them the ability to have high energetic barriers, surpassing 1000 or even 2000 K. Although such high barriers have been seen before in pentagonal bipyramidal complexes,^1^ their blocking temperatures are much lower; the highest of them [Dy(Cy_3_PO)_2_(H_2_O)_5_]Cl_3_, is “only” 20 K. Nowadays, such behavior is usually explained by the Raman relaxation mechanism. This mechanism allows for a reversal of magnetization before reaching the temperature at which the Orbach relaxation process becomes dominant.^7^

We already know that the magnetic relaxation energy is preferably transferred via vibrations in the surroundings to the thermal bath, e.g., through the low-energy vibrational modes of the molecule or crystal lattice vibrations (phonons). Unfortunately, first-principles calculations of the precise phonon spectrum of a molecular crystal (see ref (45c) as an unprecedented model example using a frozen solution) to the required precision are only possible if the crystal structure is available, either experimentally or from periodic DFT methods. However, this method is not feasible for molecular structures that have been calculated ab initio by molecular engineering. Hence, the next best approximation is a study of how the spin degrees of freedom couple to the molecular vibrations. This provides at least the initial stage of the mechanism that eventually leads to the dissipation of energy. The hope is that this initial stage, which is specific to a given molecular system, allows a comparison of related molecular systems, because both optical and acoustic phonons at least partially interact with molecules through their vibrational modes, in the part of the spectrum of their respective energies.^8^ Thus, a careful study of how the spin degrees of freedom couple to the molecular vibrations is indispensable to provide insight into relaxation mechanisms.

This approach allows vibrational modes to be identified along which the spin-Hamiltonian (SH) parameters of the system significantly change. Such vibrations are labeled as “active” vibrations. Identifying such vibrational modes provides the insight necessary to engineer new systems, in which these modes are affected through the chemical modification of the molecular structure (e.g., the modification, addition, or removal of substituents or functional groups). The goal is to obtain a system in which magnetic energy transfer becomes less efficient, which leads to longer relaxation times and higher blocking temperatures. Similar ideas have been pursued for the [Dy(Cp^*t*Bu3^)_2_][B(C_6_F_5_)_4_] complex,^2^ where theoretical calculations have provided evidence for the importance of C–H vibrations, when H is directly connected to the cyclopentadienyl cycle. This was demonstrated in a study of a series of similar H-substituted and alkyl-substituted cyclopentadienyl complexes where big differences in relaxation times were observed.^9^

The application of ab initio approaches to explain magnetic anisotropy in dysprosium single-molecule magnets focuses on first-principles calculations of anisotropy barriers, relaxation times, or blocking temperatures at their equilibrium geometry. A way that can predict the effective anisotropy barrier *U*_eff_ is based on the knowledge of the molecular structure and energetic positions of the thermally accessible Kramers doublets (KDs).^10^ The only methods that yield systematically correct results for such systems are based on multireference wave functions, such as the complete active space self-consistent field (CASSCF) method or extensions of the method that introduce dynamic electron correlation, such as the N-electron valence perturbation theory to second order (NEVPT2)^11^ or the complete active space perturbation theory to second order (CASPT2).^12^

A very important parameter used in the prediction of *U*_eff_ is the magnetic dipole matrix elements for transitions between KDs. An alternative method was recently proposed that uses predictions of quantum tunneling rates based on ab initio computed *g*-factors.^13^

In practice, one can employ the matrix elements of the magnetic dipole operator as a means to estimate the tunneling matrix elements. In fact, we have used such matrix elements from CASSCF wave functions with some success in the past for the in silico design of molecular systems.^14^

Based on such models, various methods have been proposed for the calculation of the blocking temperature, which ultimately is the central macroscopic property of interest. It is important to study the effective barrier rather than the “nominal” barrier that is obtained from the energetic position of the KDs calculated at equilibrium geometry. Incorporating the tunneling dynamics explains why the effective barrier does not always strongly correlate with the nominal barrier. In fact, it has been shown that the majority of the discrepancy arises from different relaxation mechanisms as discussed in the work of Aravena et al.,^7^ in which a simple approximation method was proposed for the evaluation of blocking temperatures induced by the Orbach mechanism. The results agreed with the experimental blocking temperatures in some cases of high *T*_B_ complexes; other complexes having larger barriers, but low *T*_B_, have been rationalized by the application of the mechanism of Raman relaxation. However, the limitation of this method is that it only examines the static properties, based on electronic energy levels, and applies empirical observations to estimate *T*_B_. It does not provide reliable tools for the study of relaxation times, which require an analysis of phonon dynamics to estimate the attempt time, the τ_0_ coefficient in the τ = τ_0_ × exp(*U*_eff_*/k*_B_*T*) expression of the Orbach mechanism of magnetic relaxation.

Recently, a more refined model for magnetic relaxation was proposed; this is based on a consideration of the effect of vibrational normal modes on the magnetic sublevels.^15^ These studies have been validated with existing molecules and then applied to the design of dysprosocenium complexes with the desired SMM properties.^16^

**Scheme 1:**
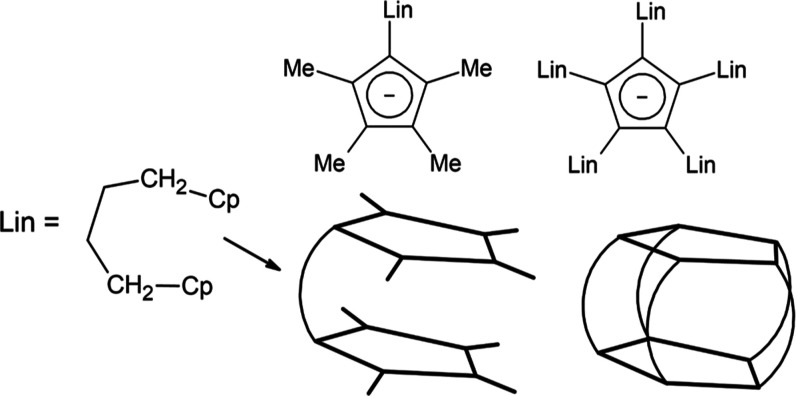
Illustration of the Key Structural Features of the Theoretical Complexes Studied

Here, we report our theoretical study of seven dysprosium systems with ligand (Me)_*n*_Cp(Lin)_5-*n*_Cp(Me)_*n*_, where Me = methyl and Lin = butylene linker between Cp rings, with various numbers of linkers and geometries. For clarity, they are listed in Table 1, and some examples are shown in Scheme 1.

**Table 1:** List of Studied Complexes **1–5**

name	number of linkers	number of methyls	linker positions	composition
**1**	1	4	1	DyC_22_H_32_
**2a**	2	3	1,2	DyC_24_H_34_
**2b**	2	3	1,3	DyC_24_H_34_
**3a**	3	2	1,2,3	DyC_26_H_36_
**3b**	3	2	1,2,4	DyC_26_H_36_
**4**	4	1	1,2,3,4	DyC_28_H_38_
**5**	5	0	1,2,3,4,5	DyC_30_H_40_

Similar linker bridged sandwich complexes have already been reported upon for transition metals with multiple structural types. Such are nickel complexes with one linker made of alkyl chains of different lengths (three, four, or six atoms) that accounts for the possibility of the inclusion of double bonds.^17^ Similar molecular structures have also been prepared with zirconium and may possibly be of use as catalysts for polymerization.^18^ Known molecular structures also exist for cobalt and rhodium, with doubly linked cyclopentadienylophane-type ligands with linkers that contain 4 carbon atoms.^19^

However, most metallocenophane-type structures are restricted to eighth group elements, especially iron and, to a lesser extent, also ruthenium. Because of this, only molecular structures with four-carbon linkers will be mentioned here, even though many different types have been reported. For iron, such compounds exist for any possible number of linkers, one of which is the so-called superferrocenophane, which is a cage with five linkers,^20^ although its synthesis is extremely complicated. An interesting effect has been observed: as the number of linkers increases, the distance between the iron and cyclopentadienyl decreases. In the case of ruthenium, only molecular structures with one^21^ or two linkers in the 1,3-positions^22^ are known.

This series of iron complexes inspired us to study similar complexes of dysprosium and the effect of molecular vibrations on the SMM parameters—the effective barriers *U*_eff_ and relaxation time along with the dependence of the number of linkers that are expected to induce rigidity and to have a desirable effect on their SMM properties.

## Computational Details

All of the calculations were carried out using the ORCA software suite. Geometrical optimization and frequency calculations were performed using the ORCA release 4.2.0. The CASSCF calculations were carried out in ORCA release 5.0.2./5.0.3.^23^ Geometry optimization with frequency calculations were done using the TPSSh *meta*-GGA functional,^24^ with a SARC2 basis set for dysprosium^25^ and TZVP bases from the Ahlrichs def2 basis set for all other elements.^26^ In these computations, ZORA scalar relativistic corrections were utilized.^27^ The RIJCOSX approximation was used to speed up the calculations^28^ using SARC/J as an auxiliary basis.^29^ With ORCA 4.2.0, large integration grid settings were used (Gridx9, Grid6, ORCA 4.2), with increased precision for dysprosium (SpecialGridIntAcc 10), along with tight SCF convergence criteria.

**Figure 1:**
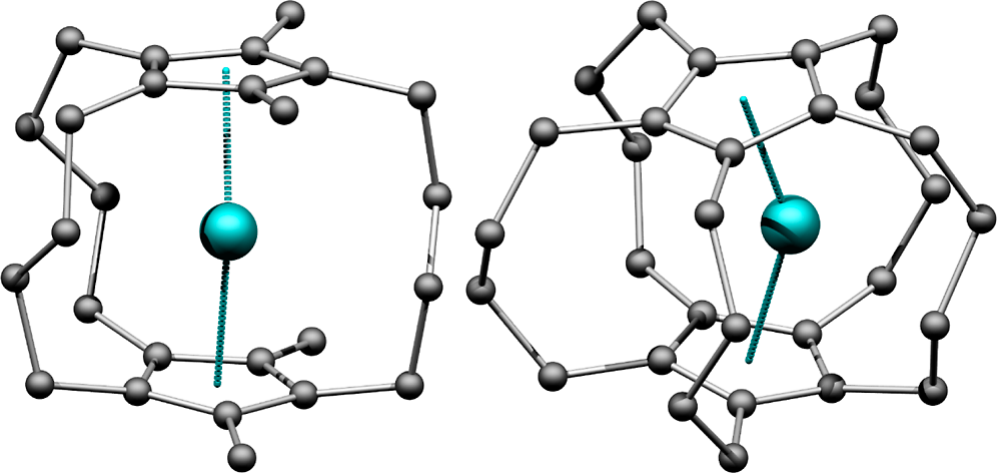
Computed molecular geometries of **3b** (left) and **5** (right). The hydrogen atoms are omitted for clarity. The dotted lines depict the centroid-Dy-centroid interactions.

The TPSSh functional was chosen because it is one of the best methods available to predict the geometries of lanthanide compounds.^30^ With this setup, optimized structures (Figures 1 and S1–S5), as well as vibrational frequencies (Table S1), were acquired. The convergence criteria were set to the “TightOpt” settings available in ORCA; if any imaginary frequencies were present, the optimization was rerun using the “VeryTightOpt” setting, and the increment in the numerical frequency calculation was set to 0.001.

State-averaged CASSCF calculations^31^ were done using the Sapporo basis set for dysprosium,^32^ along with the def2-TZVP basis set for other atoms, using the RIJCOSX approximation with an “AutoAux” automatically generated auxiliary base.^33^ The CASSCF used a set of seven active orbitals with nine electrons, CAS(9,7), which enabled the use of the ORCA ab initio ligand field theory (AILFT) module to relate the electronic structure to ligand field concepts.^34^ Relativistic effects of the CASSCF calculations were treated by the Douglas–Kroll–Hess (DKH) method.^35^ The SINGLE_ANISO program^36^ was also utilized through its interface with the ORCA program suite.

Spin–orbit coupling (SOC) within the scalar relativistic CASSCF wave functions was taken into account through the use of quasi-degenerate perturbation theory (QDPT). This produces a relativistic (field free) energy spectrum composed of KDs. Using these relativistic states, the matrix elements of the magnetic dipole operator were evaluated and further used to compute tunneling rates.

The molecular structures and related properties were modeled and viewed through the use of Avogadro,^37^ Mercury,^38^ and VESTA.^39^

## Results and Discussion

### Optimized Geometries and IR Spectra

The geometrical parameters of the optimized structures are listed in Table 2, and the coordinates of the optimized geometries are attached in a Supporting Information file. Visualizations of the structures are shown in Figures 1 and S1–S5.

**Table 2:** Comparison of the Selected Geometric Parameters Based on Cyclopentadienyl Ring Centroids between the TPSSh-Optimized Molecular Structures **1–5** and Selected Dysprosocenium Compounds **6–7**

complex	Dy-Centr1	Dy-Centr2	Centr1-Dy-Centr2
**1**	2.297	2.298	157.12
**2a**	2.293	2.298	154.02
**2b**	2.289	2.287	154.30
**3a**	2.264	2.265	147.48
**3b**	2.253	2.243	168.92
**4**	2.197	2.203	146.82
**5**	2.154	2.154	147.24
**6** (ref (3))^a^	2.324 [2.296]	2.348 [2.284]	168.9 [162.5]
**7** (ref (2))^a^	2.338 [2.318]	2.336 [2.314]	156.5 [152.6]

a Entries in square brackets are taken from reported X-ray data subject to disorder in the case of **6**.

It follows from Table 2 that the TPSSh functional provides geometrical parameters similar to those found in the molecular structures, confirmed by experimentation, of the already known dysprosocenium compounds [Dy(Cp^*t*Bu3^)_2_][B(C_6_F_5_)_4_] (**7**) and [Dy(Cp^iPr5^)(Cp^Me5^)][B(C_6_F_5_)_4_] (**6**). In addition, we can see a clear trend; as the number of linkers increases, the molecular structure becomes increasingly deformed. If the number of linkers is 3 or higher, the distance between the ring centroids and metal is shortened by about 0.05 Å for each linker added. This might contribute to the increase in instability of the complexes due to sterical deformation; however, we have decided that we will also perform calculations on these types of complexes (**3a**, **4**, and **5**) in order to study the effect of a higher number of linkers on the nuclear vibrations and their vibronic activity.

Similar, though less pronounced, effects are also seen for ferrocene derivatives, where the Fe-centroid distances become shorter as the number of linkers increases. It is also noteworthy, that with a smaller number of linkers, the angle between the centroids is slightly deformed from the ideal of 180° (Table 3).

**Table 3:** Comparison of Selected Geometric Parameters of Ferrocene Analogues Based on Cyclopentadienyl Ring Centroids

name (CCDC structure ID)	Fe-Centr1	Fe-Centr2	Centr1-Fe-Centr2
1,1′,2,2′-bis(tetramethylene)-ferrocene (COBSAN)^20^	1.625	1.646	173.75
(4)(3)(4)(1,2,3)ferrocenophane (BINMOA)^40^	1.638	1.639	172.19
(4)(4)(4)(4)(3)ferrocenophane (BETRUN)^41^	1.606	1.606	178.88

**Figure 2:**
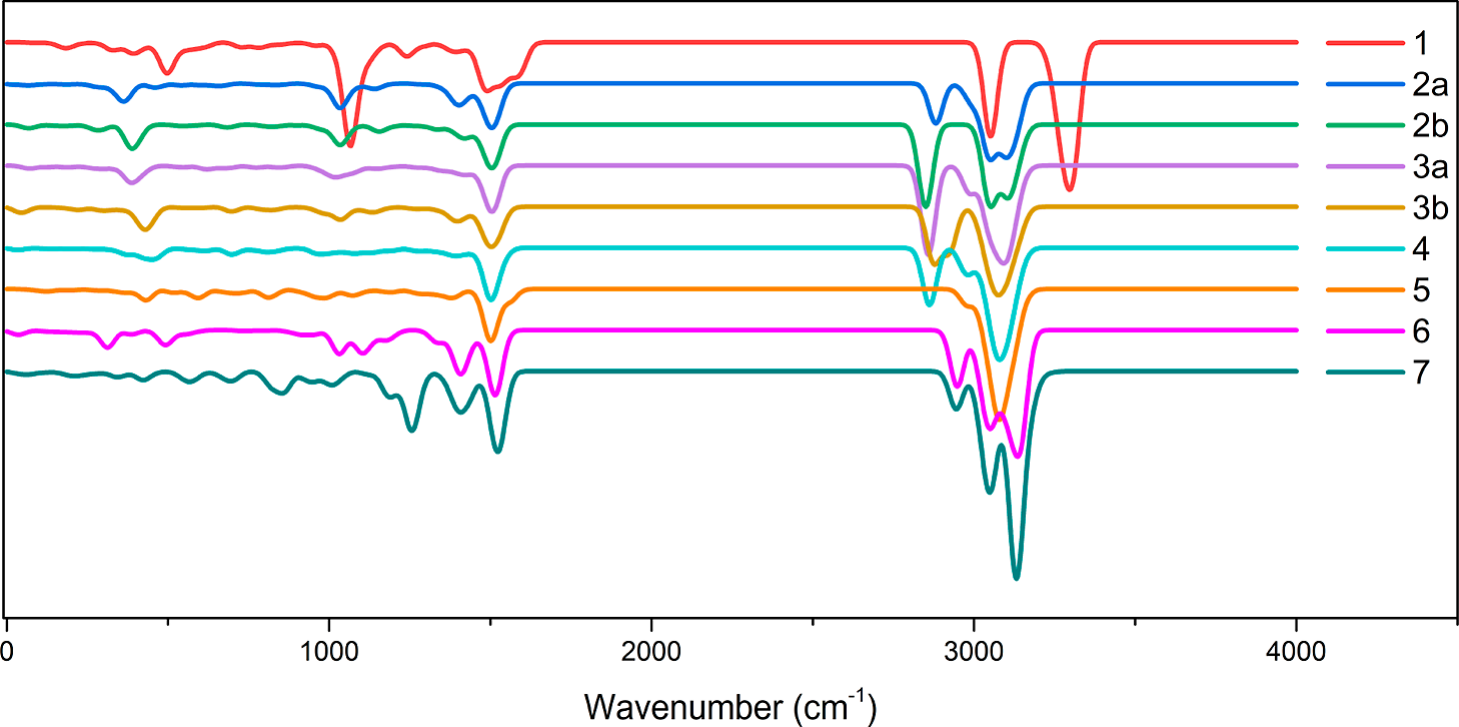
Simulated IR spectra for complexes **1–7** with the full width at half maximum (FWHM) set to 50 cm^–1^.

Computed vibrational frequencies show that none of the optimized structures have any negative imaginary frequencies, and thus, they represent true minima on the scalar relativistic ground-state potential energy surface. The vibrational spectra were simulated using the orca_mapspc program in infrared mode with the boundaries set between 0 and 4000 cm^–1^ (Figure 2). A full list of the frequencies can be found in Table S1.

The results show clear trends across the series. In the low-energy part of the spectra for complexes **1** and **2**, there are two areas with prominent vibrations around 500 and 1000 cm^–1^. As we compare complexes **1**–**5**, we see that these are slightly shifted and their intensity reduces. An inspection showed that these are vibrations from the methyl substituents on the cyclopentadienyl ring.

Similarly, interesting changes are seen around 2800 cm^–1^, and this was identified as stretching vibrations of the linkers’ methyl groups. Vibrations from carbons in the middle of a linker are at lower frequencies around 2800 cm^–1^, while vibrations from the groups in the neighborhood of the ring are slightly over 3000 cm^–1^. Complex **1** is an interesting exception; these vibrations are blue-shifted by about 200 cm^–1^, and the vibrations near to 3100 cm^–1^ correspond to the stretching of the methyl groups. In addition, in complex **5**, vibrational modes, due to the middle atoms in the linker chain, have lost their shift and are almost overlapping, creating only a shoulder at a peak of 3000 cm^–1^. Curiously, complexes **3a** and **4** have similar shoulders at 3000 cm^–1^, but in this case, these vibrations emanate from the remaining methyl groups. In complexes **2a** and **2b**, the same vibrations from the methyl groups are seen as a shoulder on the blue side above 3100 cm^–1^.

To allow comments on the rigidity of the molecular structures, we performed a very simple evaluation. Two different methods based on a comparison between the vibrational displacement vectors of selected atoms were used. The first method used to quantify vibrational displacement was a simple comparison of the sum of the sizes of the displacement vectors for each vibrational mode for the central dysprosium and neighboring carbon. In addition to the studied complexes, [Dy(Cp^*t*Bu3^)_2_]^+^ (**7**)^2^ and [Dy(Cp^iPr5^)(Cp^Me5^)]^+^ (**6**)^3^ were also added to the comparison. Their vibrational modes were calculated using the same methods applied to complexes **1**–**5**.

The second method was based on a comparison of the distribution of significant vibrations (those with a Dy atom displacement of more than 0.005 Å were considered to be significant) based on their frequency, as theoretically, shifting these vibrations toward higher frequencies should be beneficial for magnetic behavior.^42^

From this comparison (Figures S6 and S7), considering the results from the first method, it can be concluded that there is a general trend in the series of **1**–**5**. An increase in the number of linkers induces a decrease in vibrational displacement. However, complexes **6** and **7** have even lower values of vibrational displacement than **5**. Therefore, the expectation that a higher number of linkers restricts vibration seems to be at least partially correct; however, it also seems that nonlinked complexes, with substituted Cp rings, can also provide complexes with high rigidity.

An advantageous feature of the proposed linked complexes **1**–**5** is that they lack significant molecular vibrations in the area between 0 and 100 cm^–1^, especially complexes **4** and **5**, in contrast to the nonlinked complexes **6**–**7**, which have vibrations that are generally shifted toward lower frequencies. Thus, this justifies the concept that by adding linkers, it is possible to shift phonons to higher energies.

### Electronic Structures

**Figure 3:**
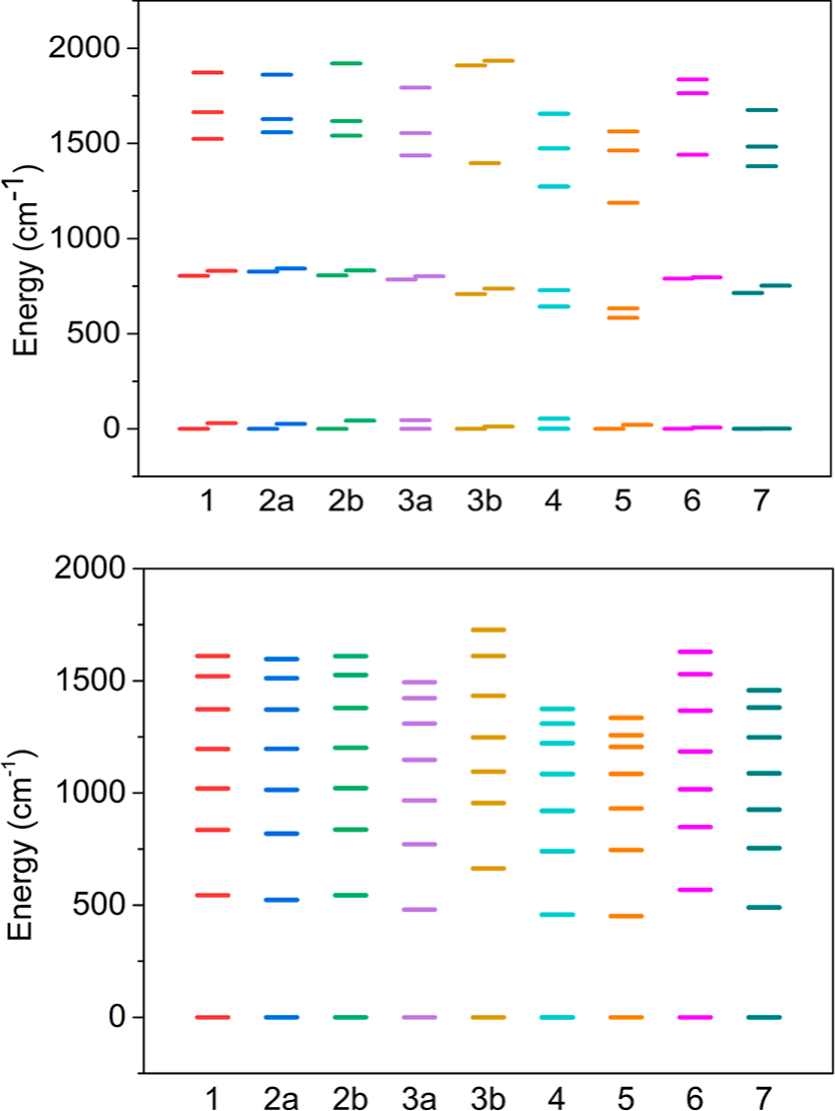
Energy splitting of f-orbitals (top) and Kramers doublets (bottom) of the ^6^H_15/2_ term for complexes **1**–**7**.

The CASSCF-based scalar relativistic f-orbital orbital energies (obtained from an AILFT analysis) and relativistic many particle spectra (originating from the ground ^6^H_15/2_ term) are shown in Figure 3 and are documented in Table S2.

The highest energy splitting of both the f-orbitals and KDs is observed for complex **3b**. However, the general trend across the series is that as the number of linkers increases, the ligand field splitting decreases for both KDs and f-orbitals (which are of course related).

The correlation between the ligand field splitting of the ^6^H_15/2_ ground state of Dy^3+^ into eight KDs with geometric parameters again confirms the general trend that ligand field splitting increases as axiality increases (Figure 3).^43^

**Figure 4:**
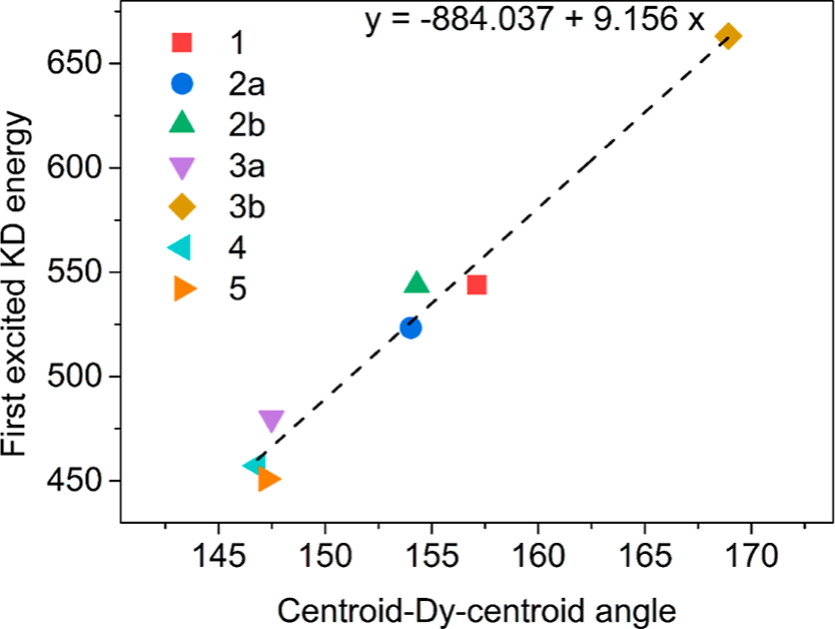
Correlation between the centroid-Dy-centroid angle and Kramers doublets (KD) energy splitting (cm^–1^) in the studied complexes **1**–**5**.

A strong correlation was found between the energy of the lowest excited KD and the angle defined by the ligand centroids and dysprosium atom; this confirms that axiality has the strongest impact on the energetic splitting of KDs. Interestingly, no similar correlation was found between the bond length and ligand field splitting. This correlation is particularly pronounced between **3a** and **3b**, which are both on different ends of the correlation line; this can be traced back to differences in the centroid-Dy-centroid angles, 148° for **3a** in comparison with 170° for **3b** (Table 2, Figure 4).

**Figure 5:**
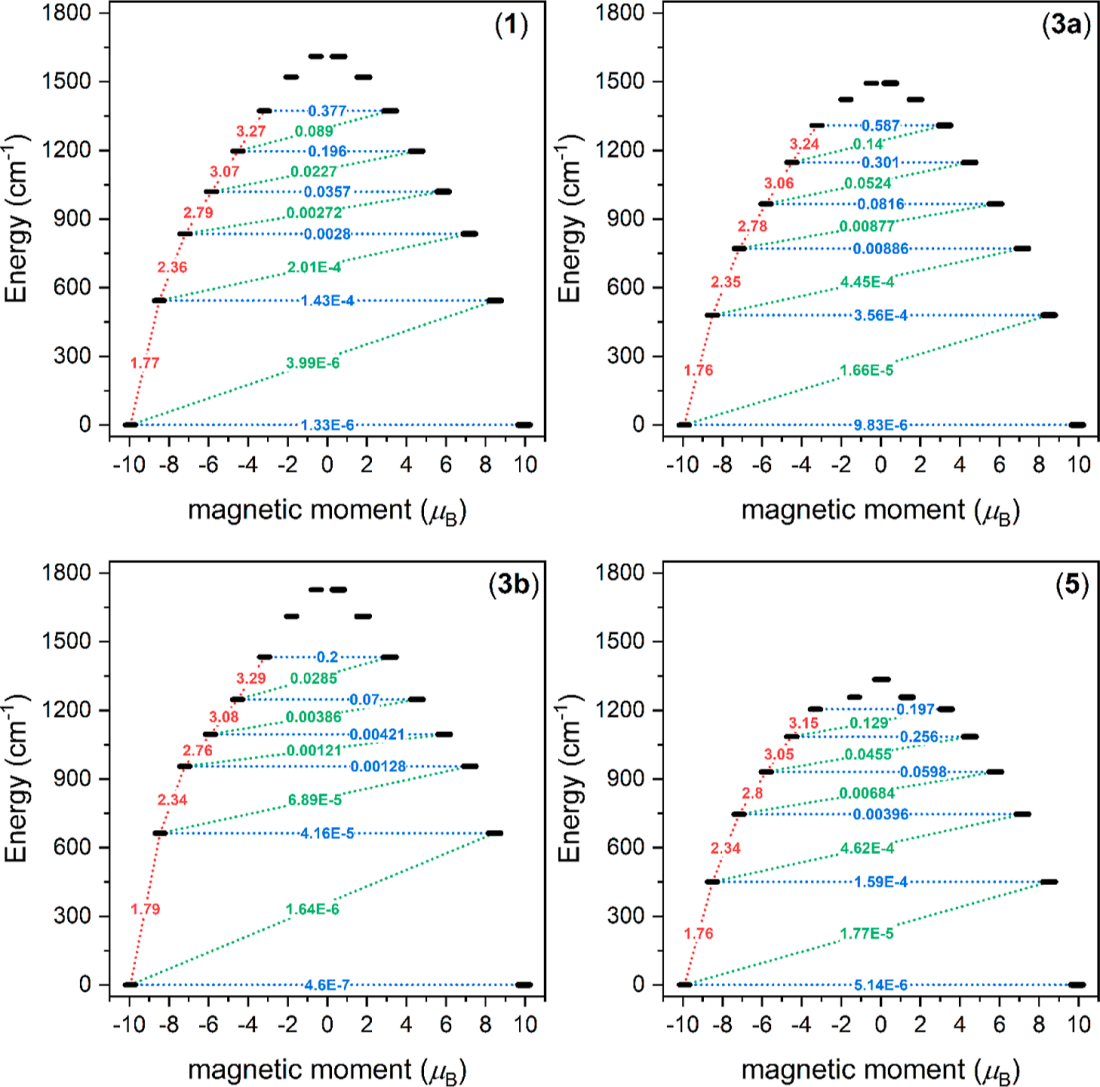
Visualization of the ab initio demagnetization magnetic dipole matrix elements for complexes **1**, **3a**, **3b**, and **5**. The numbers presented for the lowest six doublets represent the corresponding matrix element of the transverse magnetic moment (for values larger than about 0.1, an efficient relaxation mechanism is expected). Dashed blue lines refer to (temperature-assisted) quantum tunneling.

Next, we calculated the matrix elements for the magnetic transitions between the KDs and quantum tunneling rates; these are listed in Table 4 and depicted in Figure 5 (see also Figures S8–S10). The calculated *g*-factors for each KD are listed in Tables S3–S9.

**Table 4:** Energy of Kramers Doublets and Estimated Quantum Tunneling Rates

1	*E* (cm^–^^1^)	0	544	836	1020	1197	1373	1520	1611
	QTM	1 × 10^–^^6^	1 × 10^–^^4^	0.003	0.036	0.196	0.378	1.101	3.595
**2a**	E (cm^–^^1^)	0	523	819	1014	1197	1371	1512	1597
	QTM	1 × 10^–^^6^	1 × 10^–^^4^	0.002	0.031	0.188	0.279	1.055	3.422
**2b**	E (cm^–^^1^)	0	544	837	1022	1201	1379	1526	1610
	QTM	3 × 10^–^^6^	3 × 10^–^^4^	0.009	0.073	0.231	0.599	0.961	3.500
**3a**	E (cm^–^^1^)	0	480	771	966	1148	1309	1422	1494
	QTM	1 × 10^–^^5^	4 × 10^–^^4^	0.009	0.082	0.301	0.586	1.624	3.200
**3b**	E (cm^–^^1^)	0	663	955	1095	1248	1433	1610	1727
	QTM	5 × 10^–^^7^	4 × 10^–^^5^	0.001	0.004	0.070	0.202	1.012	3.590
**4**	E (cm^–^^1^)	0	457	740	921	1085	1222	1309	1375
	QTM	2 × 10^–^^5^	6 × 10^–^^4^	0.013	0.114	0.318	1.252	2.738	3.159
**5**	E (cm^–^^1^)	0	451	746	931	1085	1205	1258	1335
	QTM	5 × 10^–^^6^	2 × 10^–^^4^	0.004	0.060	0.256	0.196	2.945	0.779
**6**	E (cm^–^^1^)	0	569	848	1016	1185	1367	1530	1629
	QTM	1 × 10^–^^7^	1 × 10^–^^5^	0.001	0.002	0.047	0.097	0.365	3.496
**7**	E (cm^–^^1^)	0	490	754	926	1088	1248	1381	1458
	QTM	6 × 10^–^^8^	2 × 10^–^^5^	3 × 10^–4^	0.001	0.032	0.238	0.307	3.508

**Figure 6:**
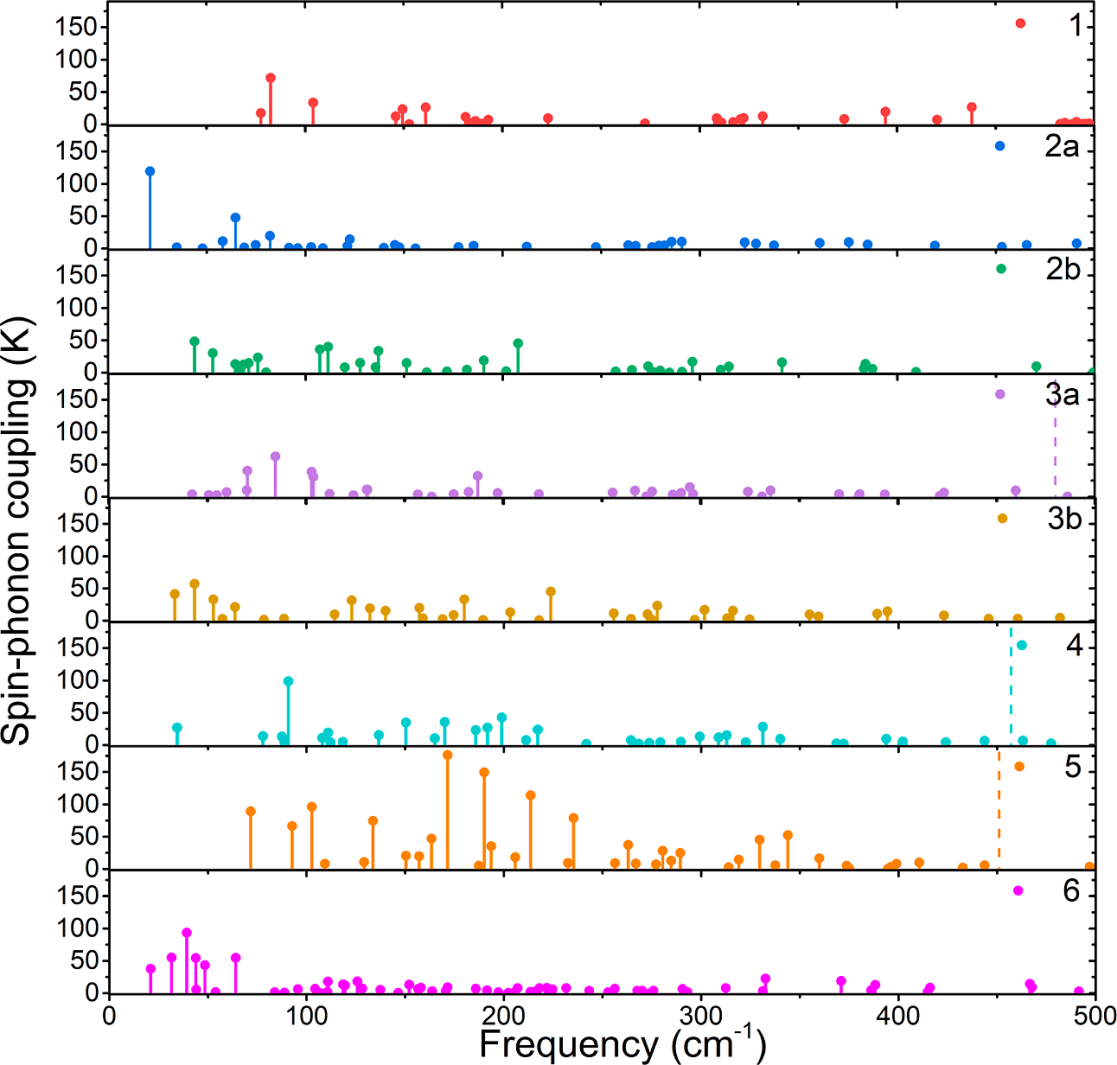
Comparison of the spin–phonon coupling parameters |∂*U*_eff_^TI^/∂*q*_α_| for complexes **1**–**6** calculated using eq 4.

**Figure 7:**
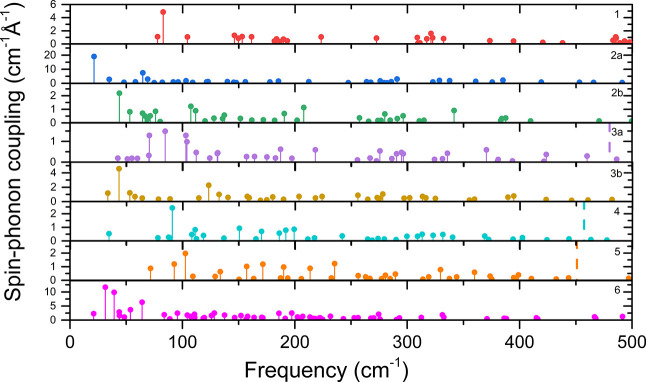
Comparison of the spin–phonon coupling parameters |∂*B*_*m*_^*l*^/∂*q*_α_| for complexes **1**–**6** from eq 6.

We have also attempted to estimate the effective barrier for the relaxation of magnetization (Table 5) using a method developed elsewhere^10^ with suitable modifications.^14^ The effective barrier *U*_eff_ was calculated using
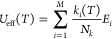
1where *M* is the number of KD states (*M* = 8 for Dy^III^), *E*_*i*_ is the energy of the respective state, and *N*_*k*_ is the normalization factor for *k*_*i*_(*T*) defined as *N*_*k*_ = Σ_*i*_*k*_*i*_(*T*). Finally, *k*_*i*_(*T*) are the demagnetization rates for the respective states calculated as
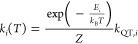
2where *k*_QT,*i*_ are the demagnetization magnetic dipole matrix elements listed in Table 4, related to the quantum tunneling within the given KD, *Z* is the partition function, and *k*_B_ is the Boltzmann constant [see the Supporting Information for the MATLAB script that details the calculation of *U*_eff_(*T*)].^44^ The temperature-dependences of *k*_*i*_(*T*) and *U*_eff_ for complexes **1**–**5** are plotted in Figures S11–S19.

**Table 5:** Estimated Magnetization Blocking Barriers and Blocking Temperatures, Calculated for a Temperature of *T* = 300 K from eq 1, and Temperature-Independent *U*_eff_, Calculated from eq 3

complex	**1**	**2a**	**2b**	**3a**	**3b**	**4**	**5**	**6**	**7**
*U*_eff_/K	2069	2072	1991	1888	2306	1777	1749	2233	2023
*T*_B_/K	73.9	74	71.1	67.4	82.3	63.4	62.5	79.8	72.3
*U*_eff_^TI^/K	2239	2228	2221	2057	2423	1880	1805	2315	2069

In their article, Aravena et al. have also suggested an approximation method for the prediction of the blocking temperature of the Orbach mechanism by dividing the theoretical energetic barrier by 28. This was derived in a recently published article^7^ from the formula of relaxation time, with the assumption, that *T*_B_ is temperature, when τ = 100 s, and τ_0_ ≈ 10^–11^ to 10^–12^. This has been shown to agree to an acceptable degree with the experimental values. The computed values of *U*_eff_ and *T*_B_ for complexes **1**–**5** are summarized in Table 5, where complexes **6** and **7** are also added for comparison purposes.

However, *U*_eff_(*T*), given by eqs 1 and 2, is temperature-dependent, while spin–phonon coupling is not. To overcome this controverse, we introduce a temperature-independent effective *U*_eff_^TI^ as an auxiliary quantity as per eq 3
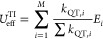
3by simply omitting the Boltzmann factors in eqs 1 and 2. Theoretically, this should serve as a limiting value for *U*_eff_, which considers equal occupation of all KDs.

These results show that once again, **3b** is an outlier among the calculated *U*_eff_ values for the complexes and is predicted to have a barrier that is at least 200 K higher than the rest. This correlates well with the predicted structural correlations discussed above. Computed values of *U*_eff_, *T*_B_, and *U*_eff_^TI^ for complexes **6** and **7** follow the order of parameters extracted from the interpretation of the experimental a.c. and d.c relaxation data **7**: *T*_B_ = 60 K, *U*_eff_ = 1761 K,^2^ and **6**: *T*_B_ = 80 K, *U*_eff_ = 2219 K.^3^

### Spin–Phonon Coupling

Phonons are vibrations in the crystal lattices. In the context of SMMs, they function as transmitters of energy to/from the magnetic centers that are in contact with the thermal bath (heat transfer) under the condition of thermal equilibrium. Recently, the need to include the effects of phonons to compute magnetic relaxation times has risen, and several methods to quantify their effect have been developed.^45^ In a nutshell, these methods rely on a study of the dependence of *g*-factors,^46^ zero-field splitting parameters *D* and *E*, crystal field operators,^47^ or energy separations between KDs.

Here, we introduce a new approach that takes advantage of an analysis of the impact of molecular vibration on *U*_eff_ over vibrational modes. We have attempted to use this method as it utilizes information from all KD energies and transition rates, weighted by, what should be, the most preferred relaxation pathway
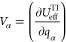
4where *U*_eff_^TI^ is calculated using eq 3 and *q*_α_ are normal modes.

From a computation point of view, the evaluation was done through the displacement of atomic positions in Cartesian coordinates by 0.05 Å in all directions and recomputing the CASSCF electronic structure with the deformed structure. The resulting values were transferred into dimensionless normal modes (eigenvectors of a Hessian matrix)
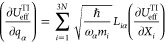
5where α corresponds to the respective normal mode, *m* is the atomic mass, ω_α_ is the vibration angular frequency, and *L* is the Hessian eigenvector matrix, and the summation is carried out over 3*N* Cartesian coordinates (*X*). As the normal modes are dimensionless, the resulting values of |∂*U*_eff_^TI^/∂*q*_α_| are given in units of *U*_eff_, which are the energy equivalents of K that are usually used for *U*_eff_. The outcome of these calculations for **1**–**6** is shown in Figure 6.

Only phonon modes below 500 cm^–1^ are shown as it is important to consider mostly those modes which are achievable by thermal excitations at lower temperatures. It is also noteworthy to mention that this is the energy range where vibrations involving close coordination environment take place. Necessarily, these will induce the largest changes of the magnetic anisotropy with molecular distortions. All of the calculated spin–phonon coupling coefficients are listed in Tables S10–S17.

The effect of spin–phonon coupling on magnetic relaxation will be now discussed in the light of the following points of departure: (i) lowest energy vibrations with maximum values of |∂*U*_eff_^TI^/∂*q*_α_| will tend to dominate Raman relaxation times and (ii) vibrations with maximum |∂*U*_eff_^TI^/∂*q*_α_| values in the vicinity of the lowest excited KD, considering vibrational line broadenings of the order of 10 cm^–1^ (see refs (45c),^45d^ and references cited therein) will mostly affect Orbach relaxation times.

For complex **1**, the vibration with the strongest coupling is the one at 83 cm^–1^. In this mode, the cyclopentadienyl rings move away from each other and modulate the centriod1-Dy-centroid2 angle, disturbing axiality (see animation comp1vibr83.gif). Interestingly, this vibration resembles the most active vibrations, which were found in our previous study on inorganic ring systems.^14^ However, in comparison to the other members of the series, vibrational modes of **1** are shifted to higher wavenumbers, thus tending to mitigate Raman relaxation terms. Interestingly, the lowest excited KD coincides with a vibration having a vanishingly small |∂*U*_eff_^TI^/∂*q*_α_| value (Table S10, Figure S22). This is in favor of increasing Orbach relaxation times.

In complex **2a**, the situation is quite similar to that of complex **1**; the first vibration at 22 cm^–1^ is very strongly coupled but shifted to lower energies compared to **1**, thus facilitating Raman relaxation (comp2avibr22.gif). As for **1**, overlap between the lowest KD is very small, which is expected to increase the Orbach relaxation times (Figure S23).

Unlike complexes **1** and **2a**, complex **2b** does not have a vibration that has a |∂*U*_eff_^TI^/∂*q*_α_| that is significantly larger than the others, with a maximal value of |∂*U*_eff_^TI^/∂*q*_α_| = 48 cm^–1^, a value in comparison with |∂*U*_eff_^TI^/∂*q*_α_| = 94 cm^–1^ for complex **2a**. The largest values are assigned to vibrations at 44 cm^–1^ (twisting of −CH_3_ groups and shift of the cyclopentadienyl ring, comp2bvibr44.gif) and 208 cm^–1^ (bending on linkers, comp2bvibr208.gif). Other significant vibrations are located at 53, 107, and 112 cm^–1^ (twisting of −CH_3_ groups on rings, comp2bvibr53.gif, comp2bvibr107.gif, and comp2bvibr112.gif, respectively). There are two weakly coupled vibrations in the vicinity of the lowest excited KD at 544 cm^–1^ (Figure S24).

Complex **3a** has four active vibrations; at 71 cm^–1^, a deformation of the molecule through twisting of the cyclopentadienyl rings (comp3avibr71.gif) is observed, with three further vibrations at 85, 103, and 104 cm^–1^, which move cyclopentadienyl ligands, leading to opening of the structure, similar to the active vibrations of previous complexes (animations comp3avibr85.gif, comp3avibr103.gif, and comp3avibr104.gif, respectively). There are no vibrations in the vicinity of the lowest excited KD at 480 cm^–1^, which is expected to favor increase of Orbach relaxation times (Figure S25).

For complex **3b**, the most important vibrations are at 33 and 43 cm^–1^, and again these are dominated by twisting of the −CH_3_ groups (comp3bvibr33.gif and comp3bvibr43.gif, respectively), accompanied by shifts of the cyclopentadienyl rings. Another significant vibration is found at 224 cm^–1^, with the contribution of the linker atoms (comp3bvibr224.gif). It is interesting that this molecule has a significantly smaller overlap of active vibration with the KD energies than previous complexes, particularly as far as the first excited KD at 663 cm^–1^ is concerned, where no vibrational modes are present nearby (see Figure S26).

In complex **4**, the most significant vibrations are at 34 and 91 cm^–1^, and both vibrations cause changes in the ligand–metal bonds due to the movement of one linker (comp4vibr34.gif and comp4vibr91.gif, respectively). Other prominent vibrations are at 170 and 199 cm^–1^, which are vibrations on linkers (comp4vibr170.gif and comp4vibr199.gif, respectively). We note on passing that rigidity induced by the presence of linkers leads to a shift of these vibrations to higher frequencies than in previous complexes. This is expected to suppress pathways for Raman relaxation. Interestingly, also the overlap of low KD energies, particularly the first excited doublet, with vibrations is very small (see Figure S27); as a result, no vibration is in resonance with its excitation energy.

Finally, in complex **5**, there are more significant vibrations than in the previous ones. The first three vibrations at 72, 93, and 103 cm^–1^ are the most important; all of them are vibrations of linkers that cause movement of the central dysprosium atom (comp5vibr72.gif, comp5vibr93.gif, and comp5vibr103.gif, respectively). There are other important vibrations; the highest of them is at 190 cm^–1^, which is vibration that causes movement in the cyclopentadienyl rings, similar to that described in other complexes (comp5vibr190.gif). There is a weak overlap between the lowest excited KD at 451 cm^–1^ and a neighboring vibration at 444 cm^–1^ (Figure S28).

Phonons have certain levels of anharmonicity, which results in broadening of their spectral line shape. Such effects are beyond all contemporary models of magnetic relaxation based on the Harmonic approximation.^48^ Anharmonicity is expected to lead to increase of the probability of nonresonant energy transfer, thus contributing to a possible increase in the relaxation rate (see refs (8),^45a^,^47^).

When comparing the phonon spectrum with the energies of KDs (Figures S20 and S21), it is visible that especially the first excited doublet in **3b** has the lowest degree of overlap with vibrations (Figure S26). Along with a higher KD splitting of complex **3b**, this is expected to contribute to the superiority of this complex over the other complexes with a smaller number of connections between rings. In particular, **2a** and **2b** have vibrations that overlap their first excited KD with large spin–phonon coupling matrix elements.

For comparison, we have attempted to use another common method for the calculation of spin–phonon coupling, which uses crystal field operators^47^
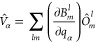
6

We have used second-order (*l* = 2) crystal field operators from the SINGLE_ANISO software for |*JM*⟩. The results suggest that to a certain degree, there is an agreement between the proposed |∂*U*_eff_^TI^/∂*q*_α_| method for the calculation of spin–phonon coupling coefficients (Figure 7).

In most complexes, the prominent vibrations arise at the same frequencies that were found when using the previous method, although their intensities may differ to a certain degree, and some disagreements might be found, for example, among the lowest-level vibrations of complex **6**, where |∂*U*_eff_^TI^/∂*q*_α_| shows quite a large number of active vibrations than for |∂*B*_*m*_^*l*^/∂*q*_α_|. Despite this, we think that |∂*U*_eff_^TI^/∂*q*_α_| is an interesting alternative method, as it is tied to *U*_eff_, a property that is inherently important for magnetic relaxation, and it also respects the probability of the relaxation pathway due to the use of magnetic moment matrix elements in the calculation.

## Conclusions

In this work, a theoretical study of seven hypothetical double-decker dysprosium complexes was carried out with one to five functional butylene groups connecting the two axial cyclopentadienyl ligands. Complexes of this type have been reported to function as SMMs with record magnetization blocking temperatures.^2,3^ The aim of the study was to analyze the effect of the number of linkers and geometry on the vibrational spectra, ligand field splitting of the ^6^H_15/2_ ground-state multiplet, and SMM properties using correlated CASSCF calculations.

We applied a model put forward by Aravena et al.,^7,10^ which was shown to reproduce effective demagnetization barriers and blocking temperatures reasonably well for a series of well-documented Dy^3+^-based SMMs. In this model, magnetic dipole matrix elements for each KD and its CASSCF energies were employed in the calculation of its contribution to the thermally assisted quantum tunneling rates and effective demagnetization barriers. When applying the model to the series of complexes studied here, with the use of DFT-optimized geometries, we came to the conclusion that complex **3b**, with the highest axiality (as reflected by the angle contended between the centroids of the two ligands and Dy, 170°), outperformed the other complexes in the series. These considerations, based on the assumption of a frozen geometry, have been extended to account for the entire set of molecular vibrations of each complex. More specifically, the linear derivatives of the energies of the eight KDs and the matrix elements of the magnetic dipolar transitions that connect components with magnetizations of the opposite sign were used to compute the (∂*U*_eff_^TI^/∂*q*_α_)_o_. This approach is an alternative method for the expression of spin–phonon coupling, which utilizes the influence of molecular vibrations on the energies of KDs and the transition probabilities between magnetic sublevels. We see some advantages to this method, such as the clear relation to perhaps the most important characteristic of SMMs, and also to its ability to weigh the individual contribution of vibrations, through their role in the relaxation pathway. A comparison with the frequently used |∂*B*_*m*_^*l*^/∂*q*_α_| method shows similar vibrations active in magnetic relaxation.

From this approach, one thing we observe is the energetic profile of the vibration, where complexes **1** and **5** are different from the other complexes studied as their vibrations are shifted toward higher frequencies, which should be beneficial due to the lower thermal population of active modes. From the viewpoint of values of spin–phonon coupling, it seems to be quite the opposite. In the middle of the series, complexes **2b–4** seem to have better parameters in terms of their lower values of spin–phonon coupling in low-lying vibrational modes. Specifically, complex **3b** is also interesting as it has a low overlap between the vibrational modes and electron transition, and in combination with a predicted high barrier and reasonably low spin–phonon coupling, it is, in our opinion, the most promising complex from the series that was studied.

## Supporting Information Available

Molecular geometries of complexes **1–5**, visualizations of their molecular structures, additional plots of the magnetization blocking barriers, lists of molecular vibrations and spin–phonon coupling coefficients, and Python and MATLAB scripts for the calculations of the spin–phonon coupling (PDF)
Optimized molecular structures 1–7 (XYZ)
Molecular vibrations (ZIP)

## Supplementary Material

ic3c02916_si_001.pdf

ic3c02916_si_002.xyz
