# Supplementary material for: Theoretical Magnetic Relaxation and Spin–Phonon Coupling Study in a Series of Molecular Engineering Designed Bridged Dysprosocenium Analogues
Source: Inorg Chem. 2023 Oct 9;62(42):17499–509. doi: 10.1021/acs.inorgchem.3c02916 (PMC10598879; doi:10.1021/acs.inorgchem.3c02916)
Supplement: Supplementary file 1 — ic3c02916_si_001.pdf [file ic3c02916_si_001.pdf]

Supporting Information

to

A theoretical magnetic relaxation and spin-phonon coupling study in a series of molecular engineering designed bridged dysprosocenium analogues

Kamil Kotrle [a], Mihail Atanasov [b,c,], Frank Neese [b], Radovan Herchel [a,*]*

[a] Department of Inorganic Chemistry, Faculty of Science, Palacký University Olomouc, Olomouc CZ-77146, Czech Republic

[b] Max-Planck-Institut für Kohlenforschung, Mülheim an der Ruhr D-45470, Germany.

[c] Institute of General and Inorganic Chemistry, Bulgarian Academy of Sciences, 1113 Sofia, Bulgaria

Corresponding Authors:

Mihail Atanasov, E-mail: mihail.atanasov@kofo.mpg.de

Radovan Herchel, E-mail: radovan.herchel@upol.cz

Table S1: List of vibrational frequencies in cm^{-1} for studied complexes 4
Table S2: Calculated energies of f-orbitals of studied complexes 11
Table S3: Calculated g-factors for Kramers doublets for 1 11
Table S4: Calculated g-factors for Kramers doublets for 2a 11
Table S5: Calculated g-factors for Kramers doublets for 2b 11
Table S6: Calculated g-factors for Kramers doublets for 3a 11
Table S7: Calculated g-factors for Kramers doublets for 3b 12
Table S8: Calculated g-factors for Kramers doublets for 4 12
Table S9: Calculated g-factors for Kramers doublets for 5 12
Table S10: List of spin phonon coupling coefficients for 1 13
Table S11: List of spin phonon coupling coefficients for 2a 17
Table S12: List of spin phonon coupling coefficients for 2b 21
Table S13: List of spin phonon coupling coefficients for 3a 25
Table S14: List of spin phonon coupling coefficients for 3b 30
Table S15: List of spin phonon coupling coefficients for 4 35
Table S16: List of spin phonon coupling coefficients for 5 40
Table S17: List of spin phonon coupling coefficients for 6 45
Figure S1: The computed molecular geometry of 1. The hydrogen atoms are not shown for clarity. The centroid-Dy-centroid contacts are depicted by dotted lines. 51
Figure S2: The computed molecular geometry of 2a. The hydrogen atoms are not shown for clarity. The centroid-Dy-centroid contacts are depicted by dotted lines 51
Figure S3: The computed molecular geometry of 2b. The hydrogen atoms are not shown for clarity. The centroid-Dy-centroid contacts are depicted by dotted lines. 52
Figure S4: The computed molecular geometry of 3a. The hydrogen atoms are not shown for clarity. The centroid-Dy-centroid contacts are depicted by dotted lines. 52
Figure S5: The computed molecular geometry of 4. The hydrogen atoms are not shown for clarity. The centroid-Dy-centroid contacts are depicted by dotted lines 53
Figure S6: Sum of all (3N-6) vibrational displacement vectors for selected atoms, used to quantify rigidity of studied complexes. Selected atoms were central Dy atom, and ten carbon atoms, connected directly to Dy. Sum of displacement is divided by number of vibrations for respected complex, and also by number of atoms used for calculation (1 for Dy, 10 for C). 54
Figure S7: Comparison of numbers of significant vibrations, and their positions on energy scale. Significant vibrations are selected as those, which has displacement vector of Dy atom larger than 0.005 Å 55
Figure S8: Visualization of *ab initio* magnetization blocking barrier for complex 2a 56
Figure S9: Visualization of *ab initio* magnetization blocking barrier for complex 2b 56
Figure S10: Visualization of *ab initio* magnetization blocking barrier for complex 4 57

Figure S11: Temperature dependence of calculated U_{eff} (left), and relative contribution of each Kramers doublet to the relaxation calculated as $k_i(T)/N_k$ (right) for 1 58

Figure S12: Temperature dependence of calculated Ueff (left), and relative contribution of each Kramers doublet to the relaxation calculated as ki(T)/Nk (right) for 2a 59

Figure S13: Temperature dependence of calculated U_{eff} (left), and relative contribution of each Kramers doublet to the relaxation calculated as $k_i(T)/N_k$ (right) for 2b 59

Figure S14: Temperature dependence of calculated U_{eff} (left), and relative contribution of each Kramers doublet to the relaxation calculated as $k_i(T)/N_k$ (right) for 3a 59

Figure S15: Temperature dependence of calculated U_{eff} (left), and relative contribution of each Kramers doublet to the relaxation calculated as $k_i(T)/N_k$ (right) for 3b 60

Figure S16: Temperature dependence of calculated U_{eff} (left), and relative contribution of each Kramers doublet to the relaxation calculated as $k_i(T)/N_k$ (right) for 4 60

Figure S17: Temperature dependence of calculated U_{eff} (left), and relative contribution of each Kramers doublet to the relaxation calculated as $k_i(T)/N_k$ (right) for 5 61

Figure S18: Temperature dependence of calculated U_{eff} (left), and relative contribution of each Kramers doublet to the relaxation calculated as $k_i(T)/N_k$ (right) for 6 61

Figure S19: Temperature dependence of calculated U_{eff} (left), and relative contribution of each Kramers doublet to the relaxation calculated as $k_i(T)/N_k$ (right) for 7 61

Figure S20: Spin-phonon coupling spectrum of $\boldsymbol{\partial U}\mathbf{effTI}/\boldsymbol{\partial q\alpha}$ with shown energies of Kramers doublets transitions, marking their overlap with vibrations. 62

Figure S21: Spin-phonon coupling spectrum of $\boldsymbol{\partial Bml/\partial q\alpha}$ with shown energies of Kramers doublets transitions, marking their overlap with vibrations 63

Figure S22. Spin-phonon coupling parameters $\partial U_{eff}^{TI} / \partial q_\alpha$ of vibrational frequencies in the vicinity of the transition (in red) from the ground to the lowest excited Kramers doublet (in blue) for complex 1. 64

Figure S23. Spin-phonon coupling parameters $\partial U_{eff}^{TI} / \partial q_\alpha$ of vibrational frequencies in the vicinity of the transition (in red) from the ground to the lowest excited Kramers doublet (in blue) for complex..65

Figure S24. Spin-phonon coupling parameters $\partial U_{eff}^{TI} / \partial q_\alpha$ of vibrational frequencies in the vicinity of the transition (in red) from the ground to the lowest excited Kramers doublet (in blue) for complex 2b. 66

Figure S25. Spin-phonon coupling parameters $\partial U_{eff}^{TI} / \partial q_\alpha$ of vibrational frequencies in the vicinity of the transition (in red) from the ground to the lowest excited Kramers doublet (in blue) for complex 3a. 67

Figure S26. Spin-phonon coupling parameters $\partial U_{eff}^{TI} / \partial q_\alpha$ of vibrational frequencies in the vicinity of the transition (in red) from the ground to the lowest excited Kramers doublet (in blue) for complex 3b. 68

Figure S27. Spin-phonon coupling parameters $\partial U_{eff}^{TI} / \partial q_\alpha$ of vibrational frequencies in the vicinity of the transition (in red) from the ground to the lowest excited Kramers doublet (in blue) for complex 4. 69

Figure S28. Spin-phonon coupling parameters $\partial U_{eff}^{TI} / \partial q_\alpha$ of vibrational frequencies in the vicinity of the transition (in red) from the ground to the lowest excited Kramers doublet (in blue) for complex 5. 70

Figure S29. Spin-phonon coupling parameters $\partial U_{eff}^{TI} / \partial q_\alpha$ of vibrational frequencies in the vicinity of the transition (in red) from the ground to the lowest excited Kramers doublet (in blue) for complex 6. 71

Script 1: A script for U_{eff} calculation....72

Script 2: Python script for extracting ORCA outputs into input files for Script 2 and Script 3....74

Script 3: Matlab script for calculating $\boldsymbol{\partial UeffTI/\partial q\alpha}$ spin-phonon coupling for individual vibrations 77

Script 4: A Matlab script for generating xyz files with displacement of every atom in Cartesian coordinates....82

Script 5: Matlab script for calculating $\boldsymbol{\partial Bml/\partial q\alpha}$ spin-phonon coupling for individual vibrations 85

Table S1: List of vibrational frequencies in cm^{-1} for studied complexes

1	2a	2b	3a	3b	4	5	6	7
0	0	0	0	0	0	0	0	0
0	0	0	0	0	0	0	0	0
0	0	0	0	0	0	0	0	0
0	0	0	0	0	0	0	0	0
0	0	0	0	0	0	0	0	0
0	0	0	0	0	0	0	0	0
77.6	21.3	43.8	42.6	33.2	34.4	71.6	21.03	19.69
82.7	34.8	53.1	51	43.2	77.9	92.7	31.49	28.89
104.3	48	64.5	55.1	52.8	87.5	102.7	39.28	31.67
146.1	58.2	66.7	60	57.4	88.7	109.3	43.83	34.34
149.5	64.7	68.7	70.2	63.8	90.8	129.2	44.08	39.37
152.9	69	71.3	70.6	78.3	108.1	133.7	48.41	49.64
161.3	74.8	76	84.7	88.6	111	150.4	53.82	51.93
181.6	82.1	80.1	103.1	114.3	112.2	157.2	64.1	60.24
183.6	91.8	107.5	104	123	118.5	163.5	83.81	63.53
186.5	96.1	111.6	112.3	132.3	136.7	171.6	88.84	74.87
189.7	103.1	120.1	124.4	140.1	150.4	187.4	95.6	81.04
193.1	109	127.9	131.1	157.3	165.1	190.1	104.34	83.62
223.3	121.5	135.7	131.5	159	170.1	193.7	107.28	88.79
272.4	122.6	137.2	157.1	169.1	185.8	205.9	110.66	118.6
308.8	139.9	151.5	164.1	174.6	191.7	213.6	110.91	120.88
309.4	145.5	161.6	175.2	180	199	232.6	118.58	125.35
311.1	147.8	171.9	182.7	189.5	211.3	235.4	119.54	129.32
317.2	155.9	182	187.3	203.3	217.2	256.4	125.83	168.25
320.9	177.7	190.5	197.4	217.9	241.9	263.1	128.34	168.41
322.4	185.3	201.9	218.3	223.9	264.5	266.9	137.51	173.4
332.1	212.4	207.9	255.7	255.7	268.5	277.2	146.45	188

373.4	247.4	257.4	267	264.5	273.7	280.6	152.13	195.03
394.3	263.7	265.6	272.7	272.9	279.4	284.8	156.63	200.09
420.5	267.4	273.9	275.8	274.9	289.8	289.4	158	202.06
438	275.9	276.7	286.3	277.8	299.4	314	163.73	211.72
482.9	279.4	279.9	290.5	296.8	308.9	319.2	170.67	213.53
483.7	282.1	284.7	294.9	301.7	313	329.6	171.58	221.2
485.3	285.7	291	296.5	313.2	322.7	337.6	185.85	225
485.7	290.9	296.2	324.3	316.1	331.4	344	191.49	227.01
488.9	322.7	310.7	331.4	324.6	340.1	359.9	197.29	235.3
491.1	328.5	314.8	335.7	354.9	368.6	373.8	202.33	240.19
493.2	337.6	341.6	370.5	359.5	372.2	375.2	204.9	249.99
495.5	360.8	383.1	380.4	389.2	393.8	394.6	207.08	250.23
497.7	375.5	384	380.9	394.3	402	396.1	213.53	253.36
502.5	385.1	387.5	393.6	423.1	402.2	399	216.46	254.45
506.2	419.1	409.6	421.6	445.8	424	410.5	218.13	259.46
544	453.1	470.5	423.7	460.5	443.6	432.6	221.86	263.72
549.3	465.7	499.6	460	481.8	463.1	443.6	222.57	268.03
551	491.1	520.6	486.3	527.2	477.3	497	224.72	268.23
557.1	523	531.5	504.1	532.1	502.4	507.3	231.65	279.37
575.3	532.1	533.2	523.3	535.1	508.2	510.6	243.26	279.73
585	539.4	543.1	534.5	546.5	530.2	519.3	252.85	283.57
607.5	544.7	544.8	534.7	548.9	542.8	519.5	256.26	287.75
612.7	552.5	555.8	546.3	552.6	550	532.1	267.67	289.93
618.3	557.1	563.2	556.1	561.9	553.1	556.3	270.24	290.26
627.3	597	588.5	558.3	573.8	569	564	274.6	295
724.1	600.9	589.3	616.5	598.2	576.6	593.7	275.87	298.69
737	640.8	660.3	618.4	614.9	613.2	595.3	290.57	324.03
776.9	645.6	668.7	656	693.1	620	613.8	293.06	324.07
797.5	669.5	686.1	659.7	695	678.1	623.9	312.5	337.32
833.6	683.3	687.9	686.8	703.8	690.7	664	331.32	337.54
842.6	725.3	727.1	692.9	707.9	697.4	677.5	332.61	337.84
855.4	754.3	770.1	716.8	712.8	702.1	695.7	370.95	339.82
860.8	773.5	772.8	733.5	752.9	703.9	699.4	386.19	344.62
890.1	786.7	783.2	770.1	760.5	720.5	719.3	388.28	345.5
910.6	815.9	799.1	774.9	767.8	758	731.2	414.88	355.07
958.6	823.9	816.6	782	783.9	762.7	740.3	416.08	355.1
1011.8	835.9	825.2	813.3	798.6	771	752.9	466.61	368.44
1015.1	850.6	850.8	825.2	804.8	780.5	756.4	467.74	371.04
1026.4	897.5	899.8	827.6	814.8	804.8	761.5	491.49	377.35
1029	938.8	912.8	841.8	833.1	808.7	787.7	508.42	377.69
1055.8	952.4	954.9	851.1	848.5	816.8	801.8	538.5	382.22
1061.7	966.8	961.1	881.5	890.4	826.9	805.3	541.84	382.68
1064	976.8	970.3	922.7	897.1	839.2	805.7	542.86	409.51
1065.2	980	978.6	943.7	932.2	844.8	811.1	548.63	411.89
1067.2	1000.1	992	956.6	942.1	870.9	817	550.38	422.08
1068.7	1007.4	1006.8	971.2	947.4	891.9	836.9	562.83	423.59

1071.2	1024	1030.8	972.7	952.7	929.2	839.7	563.89	449.19
1072.7	1027.7	1031.2	974.5	974	937.3	868	589.9	450.37
1074.2	1031.1	1034.5	994.3	983	944.6	873	623.31	461.3
1081.9	1032.9	1036.2	1002.3	993	947.3	907.8	627.85	461.45
1095.7	1034.3	1040.1	1005.5	1001.9	959.3	910.3	723.17	539.38
1112.5	1037.3	1041.7	1016.9	1017.7	962.9	928.5	726.64	540.98
1114	1042.7	1045.1	1022	1026.4	964.9	937.6	731.22	545.17
1117.6	1050.3	1046.4	1030.6	1032.1	991.6	938.3	750.43	545.39
1119.5	1071.5	1056.3	1033.9	1034.9	996.6	947.5	751.02	563.8
1137.8	1078.1	1059.7	1048	1037.9	999.3	952.1	802.29	565.22
1162.2	1078.8	1070.9	1050.8	1040.1	1012.7	952.5	803.73	586.71
1209.4	1089.6	1081.2	1054.9	1044.8	1015.8	960.2	866.68	587.42
1236.9	1100.7	1086.7	1069.9	1052.3	1034	971.2	874.17	633.64
1240.8	1109.3	1097.3	1076.7	1056.9	1042.6	985.6	881.69	634.32
1246.3	1126.3	1136.1	1081.9	1065.7	1047	990.6	908.76	674.9
1254.5	1140	1138.1	1089.5	1067.7	1047.1	996.4	910.64	675.77
1287.4	1155.3	1149.3	1093.4	1073.9	1054.8	1008.4	914.69	703.14
1297.3	1160.2	1152.6	1108.6	1088.2	1072.8	1010.2	917.01	707.66
1326	1170.4	1156.8	1111	1106.9	1075.3	1021.2	918.1	809.37
1354.6	1191.3	1161.9	1132.3	1126.7	1079.3	1026.5	921.66	809.87
1365.5	1200	1194.1	1146.3	1140.3	1091.5	1058.1	924.85	814.13
1393.1	1212.1	1200.2	1153.8	1142.3	1091.7	1062.3	949.75	815.61
1424.4	1251.9	1255.6	1160.1	1153.7	1105.2	1068.3	954.64	824.83
1425	1263.7	1260.5	1162.9	1155.8	1123.9	1078.7	955.95	827.73
1426.2	1282.2	1282.9	1184.2	1180.5	1130.1	1083.2	958.49	831.12
1428.2	1290.2	1285	1193.7	1181.1	1133.7	1083.9	960.66	831.55
1429.3	1326.4	1313.5	1204	1192.2	1146.6	1100.8	962.55	862
1431.5	1334	1315.5	1247.8	1205.3	1154.3	1114.3	964.25	863.68
1433.3	1342.2	1333	1251.6	1244.5	1181.1	1124.3	1011.91	922.43
1434.6	1347.3	1335.2	1263	1253.5	1181.8	1130.4	1018.89	923.04
1461.6	1353	1344.2	1275.7	1259.4	1182.9	1154.6	1029.59	923.12
1464.7	1358.4	1348.3	1278.4	1269.2	1204.2	1156.7	1032.06	923.37
1467.1	1377.4	1376.1	1281.3	1277.5	1247	1173.2	1036.16	926.52
1470.9	1382.6	1378.1	1302.9	1286.4	1250.4	1178.4	1040.34	927.9
1475.7	1395.1	1395.1	1323.1	1302.7	1265.3	1180.6	1042.4	929.34
1476.6	1400	1404.1	1324.9	1306.8	1265.7	1181.9	1076.06	929.42
1478	1405.4	1408.1	1333	1324.9	1270.4	1201.3	1076.6	933.2
1479.7	1408	1409.9	1334	1328	1274.1	1240	1095.68	933.49
1481.3	1419	1418.2	1351	1328.5	1279.7	1242.1	1097.91	941.74
1482.5	1420.3	1419	1351.8	1330	1283.2	1252.9	1114.42	942.35
1483.6	1421.4	1421.7	1358.2	1333.7	1298	1258.9	1115.49	945.63
1485.3	1423.7	1423.8	1370.1	1338.8	1300.4	1267.5	1120.54	946.05
1485.7	1427.9	1425	1376.5	1346	1305.2	1270.7	1121.49	950.45
1487.5	1430.1	1425.9	1377.6	1357.1	1312	1271.4	1128.43	950.71
1488.3	1434.8	1433.9	1380.5	1368.6	1326.6	1273.4	1130.14	953.09
1488.7	1436.3	1439.5	1395.5	1371.5	1331.2	1273.6	1130.74	954.92

1496.5	1459.4	1456.1	1401	1389.7	1334.6	1275.6	1132.47	956.39
1497.9	1459.9	1458.4	1405.9	1391.6	1342.9	1293.5	1134.85	957.61
1502.8	1470.5	1479.4	1412.7	1398.7	1351.1	1294.1	1160.67	1002.48
1515.8	1472.3	1482.2	1414.8	1402.3	1354.9	1299.7	1163.85	1003.09
1525	1485.7	1487.2	1417.4	1417.3	1363.7	1306.4	1172.32	1022.36
1531.4	1486.9	1487.6	1419.2	1421.1	1367.5	1306.6	1183.72	1022.84
1545.5	1487.8	1488.8	1424.1	1422.8	1372.3	1309.9	1184.35	1032.63
1547.2	1490.8	1490.3	1429	1423.2	1374.4	1321.3	1200.76	1033.7
1552	1492.4	1493.4	1430.7	1426.7	1376.9	1333.7	1205.58	1034.12
1556.8	1493.8	1496.5	1431.9	1431.7	1382	1336.1	1318.42	1035.03
1577.5	1496.4	1499.7	1459.1	1458.7	1389.3	1338.7	1324.6	1036.72
1583.4	1497.8	1500.6	1459.5	1467.1	1392.9	1340.9	1332.06	1037.34
1596.8	1500.6	1502.3	1472.3	1472	1397.6	1351.6	1335.71	1037.9
1600.5	1502.5	1503.2	1473.4	1479.3	1399.7	1352	1343.52	1038.17
3051.6	1504.1	1504.5	1487.4	1484.9	1404.5	1352.7	1346.3	1041.97
3236.8	1506.1	1504.7	1488.1	1487.5	1409.3	1357.2	1346.78	1042.22
3249.2	1506.6	1512.3	1489.5	1488.9	1416.8	1359.1	1353.81	1121.32
3261	1508.6	1514	1490.9	1492	1421.7	1367.4	1354.93	1121.99
3262.5	1510.8	1515.9	1491.1	1494.3	1427.6	1369.6	1357.45	1174.61
3263.7	1515.9	1519	1493.6	1495.3	1428.9	1375.5	1394.99	1175.37
3266.2	1517.7	1519.5	1494.4	1495.9	1446.6	1380.1	1395.55	1187.04
3266.6	1521.1	1522.4	1495.9	1497.6	1447.5	1380.8	1396.81	1189.98
3268.5	1524.9	1524	1496.9	1499.7	1474.8	1388.3	1400.65	1201.07
3269.7	1525.2	1527.5	1497.4	1502.3	1476.4	1388.6	1403.61	1204.47
3271.7	2881.8	2845.2	1501.8	1508.2	1481.3	1390.4	1405.85	1217.52
3274.9	2985.1	2856.3	1504.6	1510.9	1485.3	1395.5	1406.37	1219.95
3275.7	3007.4	3039.2	1508.5	1514.7	1486.7	1403.6	1407.38	1220.89
3279.3	3040.6	3039.3	1510.6	1518.1	1488.6	1404.3	1408.23	1221.36
3282	3041.8	3043.6	1511.4	1520.1	1489.9	1410.4	1415.51	1222.65
3283.2	3042	3043.7	1511.7	1522.9	1492.1	1411.2	1419.38	1224.99
3285.1	3042.9	3045.7	1514.2	1525.5	1492.8	1448.8	1420.9	1227.08
3286.3	3043.1	3048.2	1515.1	1528.2	1494.2	1449.6	1422.01	1227.17
3290.6	3051.1	3049.1	1518.5	1532.3	1496.1	1464.1	1422.9	1244.95
3291.7	3051.5	3049.6	1520.9	1533.4	1497.6	1466	1423.94	1245.37
3293.9	3053	3057.7	2853.8	2872	1498.5	1479.3	1425.14	1252.59
3294.8	3062.1	3057.9	2864	2874.6	1501.1	1480.9	1428	1253.16
3295.7	3075	3069.4	2985.4	2913.9	1503.3	1484.1	1429.62	1258.46
3296.8	3078.7	3070	2986.2	2928.5	1504	1485.2	1430.04	1259.03
3299.2	3090.6	3083	3035.7	3022.5	1506.7	1489.6	1434.33	1285.47
3301.4	3093.4	3084.2	3036.8	3024.3	1509.4	1489.9	1440.22	1289.35
3305.9	3097.7	3100	3041.8	3039.2	1511.4	1492.3	1456.05	1353.5
3308.6	3098.8	3100.1	3042.5	3045.2	1523.2	1492.5	1458.96	1356.57
3311	3099.2	3100.6	3046.2	3045.8	1534.4	1494.1	1470.85	1376.02
3311.7	3099.8	3100.7	3046.7	3060.8	1540.8	1494.5	1474.96	1376.42
3313.5	3104.6	3105.9	3061.9	3061.7	2859.6	1496	1483.97	1386.24
3314.6	3105.6	3106	3065.1	3064.4	2867	1497.1	1486	1386.55

3106.4	3107.6	3066.4	3066.6	2971	1499	1486.7	1400.38
3108.4	3108.1	3072	3071.7	2985.7	1504.4	1490.98	1400.81
3110.5	3112.4	3078.1	3071.9	3027.5	1506.3	1492.01	1401.1
3111	3113.1	3078.3	3079.3	3027.9	1509.4	1496.38	1401.76
3126.9	3117.4	3086.6	3084.9	3042.7	1512.6	1497.46	1403.23
3127.1	3118.5	3088.2	3089.1	3043.5	1525.9	1498.14	1403.86
3128.3	3129.8	3091	3091.6	3050	1561.3	1499.96	1404.79
3132.3	3131.1	3091.5	3092.8	3052.2	1562.4	1500.37	1406.39
3139.1	3134.4	3096.7	3097.2	3058.6	2975.8	1506.07	1409.49
3143.7	3135.5	3097.2	3098	3061.2	2980.4	1506.65	1409.79
3145.7	3148.3	3099.8	3099.1	3061.8	3032.8	1507.09	1415.81
3151.5	3148.9	3100.8	3103.1	3068	3033.2	1507.27	1417
		3104.6	3106.1	3069	3034.8	1508.35	1432.81
		3108.6	3108.2	3073.6	3034.8	1508.63	1433.75
		3109.7	3112.6	3075.4	3046.5	1511.93	1435.22
		3114.7	3113.6	3077.5	3046.6	1514.77	1435.66
		3115.9	3115.2	3080.4	3061.6	1515.6	1440.08
		3117	3119.4	3083.9	3064.1	1516	1441.17
		3130.2	3121.2	3087.9	3064.3	1516.87	1485.36
		3134	3124	3088.8	3064.6	1516.95	1486.08
		3143.3	3142.6	3094.8	3065	1517.99	1486.46
		3143.8	3142.9	3096.1	3073	1518.38	1487.56
		3151.2	3143.8	3098.6	3073.8	1520.07	1488.89
		3151.3	3146	3100.8	3074.2	1521.1	1492.44
				3101.1	3075.9	1522.33	1493.76
				3105.2	3079.3	1523.3	1493.91
				3106.9	3079.8	1524.12	1494.14
				3108.3	3084	1529.15	1497.05
				3110.7	3084.2	2946.3	1497.28
				3118	3089.7	2949.25	1497.77
				3119.6	3090.8	3016.21	1498.48
				3130.7	3091.1	3019.88	1500.86
				3143.2	3092	3037	1501.54
				3144.5	3092.1	3041.42	1501.94
				3150.3	3104.1	3041.91	1504.28
				3151.7	3107.5	3043.05	1504.98
					3107.8	3043.6	1507.26
					3109.9	3044.17	1507.63
					3115.4	3058.35	1513.87
					3115.9	3059.21	1514.51
					3120.1	3059.36	1514.59
					3120.9	3060.51	1514.92
					3122	3060.72	1518.84
					3123	3081.01	1519.07
					3131.2	3085.43	1520.69
					3131.9	3094.13	1521.94

3158.6	3096.25	1522.98
3159.4	3097.56	1525.68
	3098.68	1528.71
	3104.13	1529.03
	3104.17	1529.35
	3104.89	1530.17
	3106.88	1532.38
	3107.39	1533.92
	3107.83	1537.25
	3110.95	1537.95
	3112.42	2944.75
	3113.23	2947.96
	3129.08	3019.19
	3132.77	3020.82
	3133	3040.52
	3136.26	3040.6
	3136.36	3046.59
	3137.49	3046.87
	3137.82	3047.1
	3139.07	3047.82
	3139.74	3051.89
	3140.48	3052.2
	3140.74	3053.44
	3140.99	3053.5
	3143.03	3058.14
	3144.39	3058.18
	3145.87	3064.02
	3149.23	3064.13
	3150.55	3079.99
	3153.47	3080.75
	3154.29	3108.34
	3156.02	3109.14
		3120.77
		3120.91
		3124.48
		3125.93
		3127.2
		3127.66
		3129.03
		3129.48
		3132.36
		3132.52
		3132.68
		3132.82
		3133.9
		3134.01

3135.29
3135.55
3137.22
3137.32
3138.04
3138.64
3140.47
3140.81
3143.24
3143.46
3144.19
3144.27
3173.06
3173.18
3177.31
3177.65
3187.27
3187.28
3230.01
3230.8
3233.04
3233.35

Table S2: Calculated energies of f-orbitals of studied complexes

**1**	**2a**	**2b**	**3a**	**3b**	**4**	**5**
0.00	0.00	0.00	0.00	0.00	0.00	0.00
29.90	25.90	43.30	45.20	12.10	53.10	21.20
803.90	825.90	806.50	785.00	708.20	642.50	583.20
829.70	842.70	832.40	801.70	736.50	728.50	632.80
1524.00	1558.50	1540.80	1436.80	1395.80	1272.40	1188.00
1663.70	1628.10	1617.60	1553.90	1909.90	1473.30	1462.50
1872.10	1860.80	1920.30	1793.10	1934.20	1655.60	1563.10

Table S3: Calculated g-factors for Kramers doublets for 1

KD Energy (cm^{-1})	g_x	g_y	g_z
0	0.00000382	0.00000418	19.88335003
543.716	0.00041692	0.00044370	16.97038524
835.451	0.00760373	0.00912562	14.34912398
1019.900	0.10238952	0.11169340	11.69789762
1196.351	0.47911065	0.68775503	9.02598335
1372.804	0.52122195	1.65758976	6.35836676
1520.291	4.45632401	3.65662967	2.08781063
1610.746	1.14785123	7.13578565	13.64367712

Table S4: Calculated g-factors for Kramers doublets for 2a

KD Energy (cm^{-1})	g_x	g_y	g_z
0	0.00000381	0.00000383	19.87788146
523.220	0.00034216	0.00036720	16.98018800
818.655	0.00439436	0.00503270	14.33968678
1014.071	0.09145754	0.09620671	11.68831688
1196.584	0.46330632	0.65628346	9.03698636
1371.257	0.25980146	1.31299491	6.39637117
1511.481	3.75006738	3.67076089	2.57928889
1596.984	1.13699553	7.09188177	13.44717323

Table S5: Calculated g-factors for Kramers doublets for 2b

KD Energy (cm^{-1})	g_x	g_y	g_z
0	0.00000969	0.00001041	19.87840426
543.626	0.00073475	0.00080034	16.96876581
836.546	0.02505255	0.03130551	14.30661814
1021.408	0.20205373	0.23472936	11.62862749
1201.355	0.47882330	0.90445410	8.98830221
1379.002	1.09938970	2.49149352	6.33703708
1525.663	4.63228984	3.70602665	1.09393949
1610.112	13.38318416	7.62030218	1.21219044

Table S6: Calculated g-factors for Kramers doublets for 3a

KD Energy (cm^{-1})	g_x	g_y	g_z
0	0.00002833	0.00003066	19.85757050
479.895	0.00101929	0.00111466	17.00099831

770.982	0.02315641	0.02998312	14.30803996
966.256	0.23217229	0.25747527	11.58918264
1147.730	0.65154888	1.15219801	8.99049668
1309.070	0.98769620	2.53209829	6.43083554
1422.454	3.51382526	3.97288818	5.77595629
1493.777	0.95460070	4.68010531	14.63947359

Table S7: Calculated g-factors for Kramers doublets for 3b

KD Energy (cm^{-1})	g_x	g_y	g_z
0	0.00000134	0.00000147	19.88535543
663.123	0.00011857	0.00013096	16.88743926
954.965	0.00371661	0.00394304	14.39210045
1095.362	0.00885631	0.01624771	11.80921986
1247.808	0.19936077	0.22046127	9.06529275
1433.324	0.34136105	0.77975770	6.31969553
1610.425	3.69798487	3.51999798	2.42753769
1727.128	13.40842289	7.38051629	1.12829582

Table S8: Calculated g-factors for Kramers doublets for 4

KD Energy (cm^{-1})	g_x	g_y	g_z
0	0.00004726	0.00005961	19.83831569
457.226	0.00182730	0.00202816	17.00636315
740.054	0.03633266	0.04466680	14.31419944
920.689	0.32185274	0.36184854	11.54119464
1084.933	0.62137216	1.27692685	8.96668768
1221.693	2.93668807	4.46764879	6.20082694
1309.281	2.82382913	3.29906091	11.70929694
1375.045	0.50775885	1.69979525	16.59704742

Table S9: Calculated g-factors for Kramers doublets for 5

KD Energy (cm^{-1})	g_x	g_y	g_z
0	0.00001446	0.00001636	19.83084934
450.915	0.00045648	0.00049580	17.01100085
745.921	0.01027879	0.01343901	14.34798795
931.082	0.17848541	0.18017454	11.58066609
1085.347	0.57999969	0.95513609	9.02851946
1205.334	0.13801072	0.96747899	6.62307170
1257.521	10.52707335	7.14479306	2.66446209
1334.871	0.02708417	0.58813828	17.45741372

Table S10: List of spin phonon coupling coefficients for 1

Frequency (cm^{-1})	$\left\|\partial U_{eff}^{TI}/\partial q_\alpha\right\|$	$\left\|\partial B_m^l/\partial q_\alpha\right\|$
77.64441	17.31727	1.10418
82.66692	71.70047	4.86142
104.26694	33.46626	1.062
146.10887	12.33958	1.30821
149.54114	23.4927	0.86749
152.92066	0.24739	1.12722
161.29663	26.28052	1.07923
181.58744	11.41249	0.4393
183.56029	1.86754	0.75939
186.44526	5.04557	0.34387
189.67861	0.85994	0.72008
193.06366	7.19484	0.47222
223.24807	9.42688	1.06555
272.40508	1.4306	0.88658
308.76634	9.25397	0.97312
309.41743	0.88715	0.12129
311.05036	3.02549	0.15817
317.18945	3.3053	0.78836
320.88659	8.12074	1.59706
322.38398	9.79105	0.94994
332.12201	12.37396	0.82121
373.35417	7.97495	0.48334
394.32955	19.37115	0.44178
420.45207	7.1919	0.22834
437.99837	26.44859	0.1578
482.85849	0.83011	0.56233
483.74031	0.22022	0.45524
485.31109	2.3789	1.0703
485.73039	0.34788	0.88951
488.84717	0.42135	0.15919
491.10159	3.56617	0.04794
493.15749	0.73363	0.44071
495.52545	0.84579	0.14152
497.68624	1.48697	0.27494
502.47051	3.27292	0.74439
506.15589	4.32044	0.32225
543.94703	3.70139	0.80679
549.29208	0.34813	1.68239
550.96866	4.81098	0.74456
557.07954	0.33786	1.56176
575.31957	13.44421	1.12445
584.98471	16.68404	1.11145
607.51407	3.1814	0.37689
612.74426	9.6823	0.7479

618.28742	18.05133	0.72122
627.34063	8.05228	0.20141
724.12575	4.38936	0.34652
736.97701	2.0217	0.03174
776.90215	2.6961	0.22022
797.45278	22.79039	0.74972
833.64187	5.19058	0.34584
842.61181	11.85668	0.55103
855.42325	3.08835	0.60311
860.82217	13.14355	0.58458
890.07079	19.95392	0.37308
910.61361	7.21109	0.53626
958.58452	6.42081	0.35343
1011.80627	1.73651	0.58206
1015.11558	2.88456	0.22074
1026.35708	10.77891	0.89026
1029.03918	4.15435	0.19258
1055.79972	9.11175	0.39109
1061.64851	0.03127	0.13009
1063.9526	1.93018	0.6065
1065.18534	2.33712	0.18235
1067.19581	0.18352	0.19305
1068.65409	11.31088	0.27666
1071.21411	5.43191	0.54595
1072.73791	0.80812	0.19903
1074.24422	3.61302	0.63542
1081.94465	13.30464	0.85901
1095.67292	1.70479	0.62398
1112.45263	0.34835	0.39947
1113.98877	0.42368	0.58085
1117.55108	1.20477	0.40611
1119.49006	1.72984	0.07961
1137.76181	8.48434	0.08305
1162.17716	2.92135	0.31304
1209.44371	2.5445	0.19727
1236.87666	0.08454	0.37254
1240.79565	0.07631	0.94246
1246.33119	2.08966	0.65819
1254.53027	1.3396	0.3398
1287.35479	0.64979	0.13011
1297.34209	3.09482	0.04685
1325.94811	5.70978	0.07025
1354.61163	0.13267	0.23988
1365.47013	4.98626	0.13
1393.12776	10.50599	0.07536
1424.42747	4.11142	1.52721

1424.95666	1.51632	0.92461
1426.17309	1.37908	0.42043
1428.18393	5.08233	0.6147
1429.33918	0.92651	1.05675
1431.47842	8.4526	0.96869
1433.30252	3.15268	1.45072
1434.59576	1.99292	1.11067
1461.6294	0.14138	0.17342
1464.64806	1.66668	0.27152
1467.07243	3.68376	0.34362
1470.91537	1.60669	0.18572
1475.65091	1.80883	0.08178
1476.61221	0.53937	0.54866
1478.01844	5.02229	0.05669
1479.67029	0.52587	0.17239
1481.2534	5.45136	0.10543
1482.51184	1.5438	0.50825
1483.61163	0.96465	0.15462
1485.31148	0.85788	0.08043
1485.70396	1.59285	0.36164
1487.45028	1.31552	0.09522
1488.25844	0.23556	0.54627
1488.71191	5.37468	0.15647
1496.48964	1.69782	0.12334
1497.86639	1.16833	0.07877
1502.8008	7.47847	0.18477
1515.82364	16.6304	0.17513
1524.97316	32.7429	0.02964
1531.34722	1.80448	0.09017
1545.52045	4.34338	0.28003
1547.20438	4.14386	0.22428
1551.97345	0.96602	0.3493
1556.79542	0.78735	0.13743
1577.45576	7.56666	0.20667
1583.40397	2.68583	0.14925
1596.78125	0.8503	0.48891
1600.52309	3.39601	0.24079
3051.59852	0.88982	0.02101
3236.80395	0.41825	0.04534
3249.15006	4.31597	0.78123
3260.95647	0.54121	0.08073
3262.48501	0.86315	0.06563
3263.68714	0.35947	0.04842
3266.15779	1.5655	0.02839
3266.63719	0.70027	0.06554
3268.49495	0.69128	0.09549

3269.68861	1.65893	0.04727
3271.65756	0.98741	0.18035
3274.91388	0.55366	0.13956
3275.70866	0.53064	0.73108
3279.31192	5.13839	0.21896
3282.03937	6.12079	0.08125
3283.16858	0.43904	0.09158
3285.09097	1.50593	0.03528
3286.26589	0.40486	0.09121
3290.62969	1.22523	0.0394
3291.6982	1.02941	0.15903
3293.88016	3.54704	0.10829
3294.83321	0.26279	0.03754
3295.72993	1.06954	0.05348
3296.80811	1.14323	0.1576
3299.22967	1.17081	0.11849
3301.35203	1.11037	0.05163
3305.85023	0.33315	0.03084
3308.64453	0.63442	0.03179
3311.03526	0.35335	0.05823
3311.745	2.58349	0.23677
3313.53153	1.21903	0.10899
3314.56398	3.74952	0.30743

Table S11: List of spin phonon coupling coefficients for 2a

Frequency (cm^{-1})	$\left|\partial U_{eff}^{TI}/\partial q_\alpha\right|$	$\left|\partial B_m^l/\partial q_\alpha\right|$
21.28126	119.21237	19.0696
34.79748	1.73038	2.78021
47.94946	0.02617	0.67462
58.16033	10.89807	1.00509
64.67021	47.48713	7.47477
69.03968	1.50558	2.95629
74.83448	5.22246	0.45681
82.0563	19.32811	0.67915
91.76212	1.15469	0.96062
96.13692	0.41887	0.94101
103.07331	1.88362	1.76548
108.99335	0.21866	0.61529
121.46832	3.68636	1.05024
122.63934	14.16908	1.20623
139.92888	0.92114	1.15806
145.50757	5.22457	0.70742
147.84218	1.79177	0.45422
155.94192	0.07491	0.97714
177.71269	2.05362	0.94866
185.3413	3.9507	1.5464
212.34655	2.84553	1.06261
247.40643	2.01309	0.41782
263.71996	5.09917	1.06205
267.43847	3.98288	0.63758
275.8728	1.97104	1.67739
279.38128	4.32303	0.39854
282.05945	4.80559	0.36981
285.70659	10.3913	1.06289
290.87553	10.51509	3.01667
322.6984	9.25616	0.90531
328.49777	7.43924	2.05372
337.61534	4.56709	1.81985
360.81205	8.48808	1.35491
375.48114	9.60597	0.83137
385.07994	5.95795	2.21916
419.13016	4.31124	0.86472
453.09454	2.33717	0.63542
465.69109	5.379	0.5567
491.08943	7.82078	0.46776
523.03152	0.69969	1.62546
532.06814	5.04329	0.48534
539.43173	2.90679	0.23038
544.68197	6.63309	0.80571
552.52999	4.40167	0.19319

557.10088	3.80214	0.71884
597.01683	2.32817	0.71317
600.88527	0.36777	0.38641
640.7774	0.70603	0.39739
645.63923	0.49437	0.20272
669.4947	4.28263	0.85851
683.3077	1.7443	1.40487
725.30758	4.23564	0.83664
754.34388	0.70335	0.17886
773.4589	0.9925	0.64
786.66464	3.69174	0.63257
815.89169	8.59131	2.28935
823.84826	6.46635	0.82061
835.85183	1.61956	0.31278
850.59256	5.07062	1.34069
897.4772	1.55422	0.15512
938.79938	0.39262	0.74156
952.39413	1.04341	0.43387
966.82014	3.54812	0.34379
976.81628	2.5753	0.32266
980.01669	2.94308	0.22823
1000.09022	0.84838	0.45444
1007.43867	2.4298	0.73744
1023.99878	2.56449	1.04882
1027.65686	6.94721	0.30054
1031.0854	3.86553	0.54805
1032.88109	9.86635	0.62243
1034.28662	1.58328	0.18643
1037.32092	1.57298	0.399
1042.69584	1.19314	0.53795
1050.30122	0.49267	0.25605
1071.47686	4.03075	0.61926
1078.0856	2.01754	0.15443
1078.8444	1.24807	0.3116
1089.57375	5.74776	0.149
1100.71776	0.909	0.56812
1109.32482	6.97563	0.31997
1126.2923	6.3662	0.52606
1139.94606	0.83557	0.29719
1155.3389	6.44876	0.33856
1160.17643	2.26612	0.28649
1170.37714	5.86242	0.6624
1191.30531	2.93916	0.51396
1199.9967	1.49282	0.13823
1212.09199	3.37129	0.35449
1251.92271	0.49146	0.18144

1263.73809	3.21723	0.39123
1282.16247	0.45771	0.74891
1290.23658	2.08087	0.1987
1326.34743	0.69978	0.1993
1333.98005	0.95046	0.57441
1342.16749	1.60715	0.49627
1347.25277	0.83398	0.08977
1352.96149	5.43135	1.01596
1358.38718	0.13417	0.37032
1377.4069	7.07639	0.60936
1382.57859	1.56846	0.92085
1395.14058	1.52686	0.78708
1399.99837	3.79603	0.83044
1405.43674	2.52398	0.24094
1407.97064	1.91726	0.5198
1419.02115	3.35503	0.89912
1420.26247	3.86636	0.99184
1421.38535	2.92402	0.61897
1423.66815	11.25847	1.37334
1427.92499	2.55601	0.78348
1430.05008	1.58758	0.20294
1434.79516	0.43157	0.20547
1436.28445	5.5559	0.81882
1459.37043	6.11575	1.53804
1459.88787	1.11974	0.46825
1470.49052	5.05634	0.82839
1472.26673	2.70227	0.35672
1485.6735	1.20136	0.24855
1486.85315	0.58714	0.26243
1487.77518	0.4416	0.19749
1490.8345	2.42691	0.22574
1492.3926	0.678	0.29774
1493.81516	0.27604	0.31302
1496.41172	1.49051	0.15056
1497.81874	2.96009	0.35395
1500.57735	1.22439	0.25659
1502.48189	1.55913	0.19106
1504.06788	3.30737	0.6847
1506.07353	0.69865	0.6935
1506.54682	1.41984	0.22962
1508.57364	0.87281	0.27812
1510.7573	2.32143	0.27923
1515.87797	2.90065	0.52076
1517.69083	2.1176	0.3945
1521.1054	0.12124	0.04505
1524.88701	1.8135	0.601

1525.19122	2.75531	0.83245
2881.84125	10.26596	1.18591
2985.14223	2.58148	0.1368
3007.40805	0.98715	0.33091
3040.63943	0.31729	0.21593
3041.74955	0.57496	0.18362
3042.00175	4.279	0.26234
3042.9007	2.23845	0.11259
3043.12239	0.21661	0.1072
3051.06479	1.32701	0.21192
3051.50453	0.43915	0.43534
3052.98275	3.37436	0.39323
3062.0842	0.01898	0.087
3074.99302	0.51227	0.12089
3078.74354	2.0216	0.18451
3090.59801	2.50709	0.82221
3093.43982	1.65503	0.11479
3097.6738	1.95123	0.62656
3098.80392	1.09375	0.27951
3099.14833	0.45953	0.11514
3099.82931	1.4298	0.45814
3104.60193	0.45477	0.1033
3105.58866	1.04854	0.32934
3106.37206	0.30654	0.10987
3108.38359	1.09077	0.29189
3110.48463	0.533	0.1323
3110.9826	0.47675	0.11754
3126.94162	0.55376	0.10283
3127.1339	6.00914	1.30671
3128.32133	1.65743	0.47417
3132.31423	1.41058	0.61611
3139.10144	0.85381	0.454
3143.65915	0.31867	0.18732
3145.68344	0.24454	0.12575
3151.52325	3.49513	0.58128

Table S12: List of spin phonon coupling coefficients for 2b

Frequency (cm^{-1})	$\left|\partial U_{eff}^{TI}/\partial q_\alpha\right|$	$\left|\partial B_m^l/\partial q_\alpha\right|$
43.79988	48.09504	2.18261
53.08914	30.09882	0.81512
64.49576	12.96037	0.7109
66.67974	3.95714	0.49586
68.6954	12.552	0.27253
71.26723	14.89762	0.51766
75.94599	23.16901	0.8505
80.1041	0.28615	0.09751
107.46201	35.78646	1.21942
111.64242	39.90099	0.89261
120.07191	7.89993	0.13625
127.93026	15.16884	0.33907
135.69236	8.4736	0.31523
137.1455	33.60158	0.57216
151.48274	14.86591	0.32504
161.60391	0.18826	0.1988
171.9131	1.63475	0.22097
181.96651	4.45036	0.18448
190.52901	18.88119	0.68241
201.9133	1.91084	0.19448
207.86051	45.21327	1.13158
257.40933	1.67243	0.3657
265.61336	3.92117	0.11488
273.84504	9.80337	0.17176
276.71024	0.6147	0.18754
279.92429	2.93208	0.6596
284.67003	0.04678	0.19137
291.04188	1.45902	0.31433
296.21974	16.64362	0.51544
310.69812	4.1914	0.14403
314.76595	9.33678	0.19213
341.60459	15.84752	0.91114
383.05829	6.09899	0.17678
383.96913	13.50527	0.31326
387.51533	5.62518	0.35954
409.55522	0.96634	0.13742
470.54442	9.68316	0.13601
499.55667	0.26003	0.12885
520.55506	1.21717	0.09541
531.49786	6.30567	0.18518
533.20575	1.07061	0.11806
543.10476	0.8963	0.0825
544.77563	6.83759	0.24136
555.77368	5.73013	0.11302

563.14831	0.62479	0.16065
588.46657	4.66757	0.09441
589.30659	6.48144	0.1612
660.30233	0.12236	0.16263
668.73127	2.54594	0.32737
686.13801	5.53974	0.19624
687.88808	1.66612	0.18363
727.06024	7.57507	0.19621
770.07164	3.45822	0.29961
772.77843	1.14137	0.06003
783.19926	1.89735	0.08755
799.10829	3.65887	0.34499
816.59041	15.73013	0.2814
825.17157	4.46905	0.02104
850.74865	1.90445	0.05527
899.78065	1.15073	0.0382
912.82794	0.98268	0.37135
954.85398	7.2927	0.09521
961.13786	4.2986	0.09764
970.2676	2.89411	0.04559
978.56962	9.55424	0.1775
992.03819	0.74745	0.41597
1006.76037	0.47017	0.09646
1030.7706	2.14497	0.06077
1031.19255	6.45894	0.18521
1034.4515	0.46189	0.21278
1036.22392	0.96769	0.03225
1040.13369	2.63291	0.07267
1041.69538	1.31733	0.06284
1045.13968	5.72396	0.15569
1046.38146	2.19135	0.08848
1056.33951	4.03686	0.27635
1059.68992	0.89059	0.07882
1070.94041	1.83292	0.10604
1081.17033	2.74278	0.10261
1086.66926	2.82207	0.07803
1097.33565	12.17176	0.18425
1136.04508	1.06074	0.06317
1138.07525	0.73913	0.17074
1149.26079	7.71487	0.07178
1152.60635	0.8311	0.06455
1156.77065	4.91646	0.08608
1161.93012	2.21526	0.07091
1194.13221	0.73699	0.1957
1200.14833	0.07788	0.11874
1255.56521	0.68483	0.05035

1260.46761	2.8388	0.08989
1282.84884	3.51867	0.05101
1284.96075	0.49281	0.08628
1313.51117	5.63344	0.09868
1315.51151	5.67192	0.14283
1332.9615	0.73897	0.0354
1335.23535	1.37845	0.01749
1344.23985	1.45737	0.09213
1348.29187	2.45472	0.06818
1376.10424	0.3222	0.06136
1378.06331	0.90959	0.07365
1395.0735	2.18112	0.16282
1404.10103	0.6409	0.09086
1408.11896	0.65217	0.12742
1409.88145	1.66447	0.13904
1418.18393	0.21156	0.02795
1419.00509	1.91018	0.06312
1421.65677	0.73239	0.05466
1423.82344	2.37415	0.05702
1425.02809	11.40147	0.2349
1425.87449	9.92811	0.19138
1433.92725	3.3011	0.07206
1439.45686	0.33855	0.12603
1456.12303	0.79635	0.11312
1458.34861	1.26989	0.02148
1479.42186	1.78707	0.10001
1482.181	3.10971	0.04513
1487.19133	1.38782	0.0205
1487.61567	0.1429	0.05498
1488.75654	0.02392	0.02843
1490.2952	1.66308	0.0541
1493.43113	0.95566	0.0828
1496.50852	1.52187	0.0764
1499.70475	1.95437	0.08121
1500.58119	0.2335	0.06108
1502.31366	1.20762	0.04936
1503.22292	2.81447	0.077
1504.48478	1.68993	0.05254
1504.65631	2.1448	0.05278
1512.34203	9.02136	0.20571
1514.01237	5.01682	0.14664
1515.86966	3.43188	0.0719
1518.99853	2.64602	0.05756
1519.51566	9.54038	0.24521
1522.42427	1.46751	0.02932
1524.0311	4.2793	0.10772

1527.47539	3.20817	0.10159
2845.21945	22.9027	0.30602
2856.33868	26.87103	0.38971
3039.16443	2.5997	0.03093
3039.31989	2.12187	0.04258
3043.56265	1.23056	0.01487
3043.69801	2.58315	0.05538
3045.69046	2.358	0.14063
3048.21073	0.2271	0.07217
3049.04649	0.99271	0.03181
3049.55644	1.11003	0.04479
3057.69679	1.62706	0.02104
3057.88532	0.72303	0.03686
3069.37352	1.78012	0.03317
3069.97912	1.66457	0.03433
3083.03484	0.71406	0.05871
3084.14807	0.15809	0.06663
3099.99982	4.37983	0.07229
3100.14477	2.52817	0.05525
3100.60776	0.54887	0.01904
3100.70461	0.75401	0.02271
3105.91788	0.95527	0.02068
3105.99663	0.76355	0.02316
3107.61798	2.3658	0.07048
3108.10344	0.05076	0.03967
3112.39809	0.65226	0.0317
3113.12623	0.15809	0.01302
3117.36177	2.93761	0.06357
3118.50697	2.75254	0.06195
3129.83981	0.37823	0.03291
3131.10943	0.27758	0.01679
3134.42553	1.13667	0.0257
3135.52627	0.7575	0.01725
3148.33239	0.48275	0.01658
3148.87832	0.50933	0.01881

Table S13: List of spin phonon coupling coefficients for 3a

Frequency (cm^{-1})	$\left\|\partial U_{eff}^{TI}/\partial q_\alpha\right\|$	$\left\|\partial B_m^l/\partial q_\alpha\right\|$
42.56797	3.67311	0.1874
50.98947	2.78058	0.14683
55.07862	2.30168	0.18429
59.9856	6.90895	0.17557
70.21511	10.04878	0.32421
70.56225	40.18013	1.28574
84.7239	62.2061	1.48385
103.13914	38.45806	1.28554
104.03701	30.67171	0.98005
112.26486	4.24457	0.46236
124.40713	2.4949	0.19585
131.04805	11.04315	0.41619
131.47997	11.04421	0.44817
157.08592	3.54077	0.2658
164.1184	0.13651	0.26899
175.18105	3.93145	0.24856
182.74408	7.52353	0.19717
187.33569	32.12942	0.61807
197.43612	5.64778	0.18179
218.30166	4.01951	0.594
255.7363	6.38216	0.10225
266.98086	9.24356	0.19533
272.70093	0.29893	0.05756
275.74857	8.02083	0.53726
286.27006	3.26071	0.1674
290.49056	5.95555	0.40309
294.8854	14.72055	0.46605
296.44883	4.00588	0.40275
324.24757	7.74387	0.13478
331.38668	0.44227	0.16037
335.69414	9.81402	0.42677
370.4866	4.16683	0.59297
380.44446	0.68227	0.04912
380.89196	3.87356	0.12805
393.6124	3.61292	0.05823
421.58479	1.23057	0.03673
423.65341	6.53055	0.36754
459.9767	9.70032	0.29103
486.30232	0.18696	0.13986
504.1103	5.88292	0.10165
523.27789	3.16327	0.1256
534.47887	7.89428	0.23895
534.73066	0.41245	0.17762
546.25019	2.36082	0.14876

556.09109	3.3026	0.09753
558.25383	2.12795	0.06127
616.52942	0.23433	0.10203
618.41255	2.60354	0.06629
656.02704	0.33519	0.15803
659.66106	5.94963	0.13854
686.82387	7.75219	0.32509
692.88416	5.73924	0.06448
716.75889	2.59605	0.14592
733.47021	2.3036	0.43828
770.06559	0.4336	0.03536
774.86568	0.02135	0.08188
782.0315	3.72177	0.14354
813.31713	10.40308	0.19412
825.21863	6.10132	0.15394
827.56174	4.59537	0.0802
841.77375	2.70571	0.08719
851.07638	10.15952	0.13943
881.53598	1.24127	0.03466
922.7304	2.08986	0.16779
943.66636	1.2914	0.07848
956.54516	0.82042	0.03089
971.15446	0.14518	0.03879
972.66337	1.11016	0.0553
974.53148	1.24097	0.04591
994.29477	4.70097	0.17832
1002.26641	6.80385	0.21415
1005.44945	0.36601	0.06111
1016.90023	3.89668	0.12828
1022.02903	8.5051	0.38213
1030.61406	1.27172	0.0289
1033.92732	0.60568	0.06092
1048.0216	0.66578	0.05642
1050.77002	5.8379	0.07722
1054.94257	1.25274	0.01807
1069.93276	1.30844	0.05469
1076.64565	0.49035	0.03468
1081.90616	2.28953	0.0341
1089.51904	1.70073	0.06749
1093.42693	4.42018	0.15207
1108.5593	0.99536	0.12012
1110.946	6.17043	0.0977
1132.27265	0.9292	0.04837
1146.32968	3.42781	0.13312
1153.82581	1.71146	0.04293
1160.06105	1.8889	0.07382

1162.87283	2.40216	0.11281
1184.19817	2.20029	0.08773
1193.69039	2.86648	0.09694
1204.03841	0.07673	0.03354
1247.74853	2.88522	0.05682
1251.59012	0.97136	0.05046
1262.9798	2.37298	0.0377
1275.66227	3.23596	0.06056
1278.4007	2.23538	0.03489
1281.25045	0.26285	0.01094
1302.84709	0.61583	0.0209
1323.07508	2.36328	0.02954
1324.91813	0.72783	0.01136
1332.99678	1.2233	0.02339
1333.969	0.09101	0.01112
1350.96308	0.75334	0.04455
1351.78448	2.32622	0.08566
1358.15073	2.04443	0.03102
1370.05464	0.35552	0.08825
1376.52775	1.85652	0.03797
1377.60032	0.71245	0.03248
1380.45196	1.07916	0.02268
1395.54179	1.79903	0.08963
1400.96031	0.49135	0.04523
1405.90334	1.53615	0.11894
1412.67847	4.5761	0.11618
1414.81319	4.70487	0.09922
1417.40423	9.02442	0.09401
1419.17047	5.56938	0.06877
1424.09494	0.11014	0.05527
1429.03866	6.40955	0.05903
1430.67011	4.67793	0.10644
1431.92236	8.2926	0.27388
1459.13592	0.70183	0.14482
1459.49665	0.5287	0.03999
1472.28119	0.62144	0.15807
1473.39098	0.46256	0.09376
1487.36095	0.71822	0.04527
1488.11694	5.15915	0.05949
1489.53815	0.02524	0.04817
1490.88557	2.29774	0.01647
1491.10064	1.11634	0.01152
1493.61025	4.58882	0.05856
1494.38612	3.80696	0.03682
1495.89718	0.32298	0.01076
1496.85977	1.65579	0.04961

1497.44465	0.57743	0.02396
1501.81195	6.94637	0.14668
1504.56685	8.29285	0.16246
1508.52956	3.47237	0.06324
1510.63103	1.94872	0.12645
1511.44012	5.01424	0.09442
1511.6646	1.03923	0.11882
1514.22697	11.68564	0.2496
1515.11401	5.28342	0.13001
1518.47471	5.09056	0.09468
1520.87522	3.73389	0.08452
2853.74944	14.83319	0.34019
2864.0293	22.36333	0.49537
2985.36032	3.97878	0.05352
2986.15627	9.5521	0.15689
3035.71544	1.33392	0.01433
3036.7943	1.64099	0.03081
3041.81022	3.19051	0.06836
3042.51782	1.23213	0.0261
3046.17039	0.81769	0.01578
3046.69878	0.19393	0.01664
3061.87629	0.00714	0.0044
3065.10872	2.09191	0.02351
3066.3581	0.03311	0.02575
3072.01871	0.7517	0.01559
3078.12728	0.12891	0.02016
3078.32681	1.13921	0.03431
3086.61211	0.1429	0.00555
3088.21617	0.5048	0.00791
3090.97232	0.53127	0.0523
3091.52708	1.08418	0.0129
3096.71013	1.51642	0.03834
3097.22544	1.99409	0.04374
3099.83198	2.56404	0.02904
3100.80722	1.19784	0.03215
3104.57541	0.77487	0.03102
3108.64433	0.08652	0.0206
3109.66507	1.07652	0.02822
3114.65889	0.39774	0.0119
3115.85686	3.13199	0.06968
3116.95117	0.91228	0.01498
3130.16963	2.79383	0.05563
3133.95673	0.24162	0.01694
3143.34402	0.63427	0.04017
3143.76318	0.33242	0.02665
3151.1507	0.08257	0.02528

3151.26217	1.39047	0.0354

Table S14: List of spin phonon coupling coefficients for 3b

Frequency (cm^{-1})	$\left|\partial U_{eff}^{TI}/\partial q_\alpha\right|$	$\left|\partial B_m^l/\partial q_\alpha\right|$
33.21581	41.3243	1.18504
43.18018	57.08877	4.59113
52.77167	32.80058	1.21513
57.4162	2.57149	0.69231
63.79019	21.18896	0.47661
78.33659	1.18557	0.33454
88.54758	3.02487	0.37126
114.24797	9.71515	0.49808
123.01001	31.39608	2.26826
132.25146	19.07155	1.00295
140.11061	15.55673	0.57743
157.28445	19.68647	0.71313
159.02182	3.75602	0.5285
169.0576	2.51028	0.16017
174.61161	8.80007	0.2273
179.95456	32.78351	0.64735
189.53867	1.07582	0.31683
203.33027	13.00923	0.70699
217.9444	0.67475	0.50168
223.84733	44.87682	0.71289
255.68189	11.25064	0.88786
264.46852	2.60856	0.31856
272.91076	10.05592	0.54613
274.91267	1.53306	0.43371
277.84211	23.12222	1.04397
296.80197	1.26476	0.45786
301.71738	16.73687	0.52609
313.17209	3.76845	0.7098
316.13146	15.2597	0.51876
324.60432	1.95411	0.42781
354.90733	9.57046	0.207
359.45816	6.39257	0.32635
389.23392	10.70287	0.61414
394.3426	14.35956	0.78091
423.11084	8.15091	0.28799
445.76861	3.04313	0.16373
460.45475	3.09085	0.22352
481.83446	4.19663	0.27639
527.22972	8.3447	0.39241
532.04715	1.87535	0.38624
535.09289	9.72695	0.34832
546.48446	8.67334	0.71976
548.89405	7.44842	0.43376
552.55918	2.76811	0.24179

561.93982	5.94814	0.15857
573.75968	3.38528	0.27112
598.19417	1.25432	0.17197
614.85266	7.39019	0.48675
693.07415	10.34761	0.32201
695.01859	11.07722	0.28256
703.78866	8.81334	0.35088
707.92927	12.81891	0.67254
712.82946	13.96092	0.39036
752.89475	6.12557	0.62217
760.47795	2.96775	0.26976
767.77297	3.90355	0.25431
783.84509	16.24843	0.29986
798.62839	11.57733	1.22333
804.80197	5.32316	0.24981
814.83852	3.14292	0.14912
833.08191	5.42498	0.24709
848.49017	10.83791	0.23078
890.42259	3.75369	0.17444
897.05166	6.38893	0.34165
932.17931	10.31113	0.23264
942.09389	6.60616	0.13111
947.44372	2.54228	0.12172
952.71447	2.77666	0.29252
973.96253	0.00454	0.19979
982.97827	2.57397	0.3324
992.99178	0.12073	0.13589
1001.93843	3.00779	0.19201
1017.67584	8.21607	0.39304
1026.40492	3.70982	0.17159
1032.13336	0.09483	0.14127
1034.84661	7.15391	0.26796
1037.89632	1.66988	0.14526
1040.13676	2.39824	0.32906
1044.83142	3.1084	0.25841
1052.26437	4.22878	0.09616
1056.85515	0.14329	0.25392
1065.68383	4.54263	0.12602
1067.71485	7.68919	0.11856
1073.86957	4.91536	0.36419
1088.19635	2.51625	0.23257
1106.87019	0.18685	0.18858
1126.72759	1.45112	0.1738
1140.28231	6.64088	0.29023
1142.33562	2.86847	0.11121
1153.6634	5.23293	0.14521

1155.77636	1.371	0.22585
1180.51906	9.21855	0.37013
1181.09855	3.75088	0.24366
1192.21466	0.93661	0.14411
1205.27681	0.4424	0.14204
1244.46956	3.18387	0.07074
1253.44778	3.82185	0.13487
1259.40089	1.16349	0.17802
1269.18626	0.2738	0.12092
1277.455	6.59995	0.19435
1286.42747	6.71716	0.16584
1302.69841	3.94855	0.20962
1306.76178	3.78468	0.21123
1324.89252	0.75602	0.10531
1328.02543	4.41256	0.27236
1328.49088	7.30601	0.21517
1330.03691	4.9254	0.19727
1333.67211	3.67718	0.11633
1338.75565	1.27577	0.14703
1345.96221	2.3171	0.07936
1357.11578	6.8325	0.14541
1368.55464	0.01389	0.15293
1371.46347	2.12005	0.18773
1389.71676	0.20874	0.11385
1391.59927	3.5566	0.17906
1398.73361	1.60999	0.09636
1402.2589	2.38748	0.18052
1417.33051	1.39077	0.28376
1421.11384	5.62277	0.35175
1422.76761	5.13778	0.25293
1423.18961	13.14123	0.56965
1426.68442	4.3454	0.17234
1431.72634	1.19269	0.20851
1458.68246	1.06667	0.16478
1467.10345	0.72012	0.20275
1472.0167	0.97231	0.24032
1479.27347	2.52012	0.27809
1484.88233	2.70727	0.1324
1487.53097	2.3633	0.25055
1488.87821	0.13914	0.18231
1491.96555	1.12808	0.18815
1494.25714	0.65901	0.06373
1495.31066	0.99826	0.1593
1495.93672	0.81227	0.11139
1497.56616	0.10176	0.12422
1499.73424	1.08418	0.06406

1502.31502	2.37427	0.15284
1508.23663	7.25856	0.38704
1510.8512	2.42709	0.17754
1514.70817	4.3626	0.46604
1518.09113	0.46849	0.16578
1520.1312	1.02253	0.29733
1522.87822	0.39906	0.23465
1525.44816	3.42262	0.28109
1528.21774	4.16873	0.11664
1532.31805	12.20582	0.53254
1533.42853	12.98162	0.6947
2872.01351	21.41061	0.43801
2874.63449	13.24325	0.486
2913.86964	5.80337	0.2997
2928.48123	6.98486	0.52388
3022.47028	2.63106	0.09096
3024.34273	7.59913	0.22363
3039.1676	1.876	0.03828
3045.17535	1.71456	0.20797
3045.74519	2.66533	0.11995
3060.81836	1.51726	0.08799
3061.73268	0.96634	0.03656
3064.34868	0.16955	0.0495
3066.64491	0.92468	0.20892
3071.6724	0.749	0.14777
3071.92187	1.6899	0.16119
3079.24527	1.33869	0.06792
3084.88028	3.2531	0.10934
3089.05584	1.65048	0.10985
3091.57649	0.97756	0.05346
3092.83501	0.64366	0.05069
3097.19953	0.32594	0.14012
3097.96503	9.23891	0.43117
3099.07427	6.6964	0.29225
3103.06201	4.34378	0.15397
3106.07861	1.32634	0.10713
3108.23958	1.76147	0.12723
3112.5946	0.70207	0.1197
3113.56851	1.21462	0.06124
3115.21137	0.57555	0.22631
3119.38514	1.12907	0.13065
3121.15235	3.47461	0.29682
3123.96461	1.28682	0.13998
3142.63167	0.30652	0.17656
3142.86455	0.9413	0.28947
3143.80761	0.09824	0.17171

3145.96183	1.33025	0.12281

Table S15: List of spin phonon coupling coefficients for 4

Frequency (cm^{-1})	$\left|\partial U_{eff}^{TI}/\partial q_{\alpha}\right|$	$\left|\partial B_{m}^{l}/\partial q_{\alpha}\right|$
34.3764	26.80794	0.53657
77.88511	13.62468	0.22777
87.47095	13.08208	0.27233
88.66773	4.94253	0.22333
90.74745	98.63724	2.42381
108.05565	10.90879	0.47134
111.0047	19.10497	0.82664
112.18174	3.9475	0.15732
118.47613	4.72748	0.40669
136.69189	15.31208	0.20132
150.42865	34.97956	0.92476
165.08833	10.39915	0.15189
170.13354	35.36449	0.69832
185.83626	23.14628	0.57058
191.72778	26.86284	0.78888
199.04045	42.58754	0.85913
211.2701	7.29051	0.13078
217.21815	23.83269	0.21767
241.90217	1.77674	0.36891
264.47475	7.39792	0.1347
268.50952	1.93529	0.049
273.73972	3.51847	0.1716
279.35244	4.49286	0.14064
289.80465	5.09266	0.09873
299.38485	13.12318	0.33847
308.88259	11.61293	0.33753
313.01299	14.9702	0.49288
322.68223	4.23556	0.41409
331.37358	28.04825	0.47488
340.10157	9.51307	0.27229
368.60891	2.279	0.35716
372.17016	2.03464	0.10361
393.81079	9.49514	0.13446
401.98508	0.28429	0.06091
402.21756	5.42746	0.24761
423.98094	4.29183	0.07901
443.57968	6.50167	0.14791
463.09889	6.86591	0.09715
477.34445	2.50169	0.06492
502.34643	0.3036	0.07269
508.15267	0.01742	0.10052
530.22717	6.3683	0.22375
542.7867	10.13707	0.18993

549.94837	1.9713	0.16376
553.08187	5.30609	0.14631
568.97178	5.91877	0.09548
576.55206	5.63232	0.08466
613.19525	4.10494	0.1955
620.03373	3.76924	0.10777
678.13077	5.43711	0.13594
690.66606	16.01487	0.25523
697.37354	1.06439	0.08635
702.05435	0.48224	0.20091
703.89621	14.25963	0.36157
720.54187	1.95541	0.27972
758.00587	1.07855	0.10354
762.71962	2.2078	0.1108
770.96416	5.3591	0.11858
780.49559	0.78571	0.14165
804.83066	0.75271	0.12453
808.73228	13.6942	0.28295
816.81461	1.09722	0.08359
826.85754	1.37993	0.01939
839.2364	13.34792	0.18751
844.81329	5.32775	0.05305
870.91096	6.89765	0.12872
891.91822	7.08508	0.10226
929.23799	0.59128	0.03356
937.2689	5.58841	0.10499
944.56764	8.31352	0.06059
947.33159	7.25428	0.35492
959.32338	0.47739	0.07504
962.9136	0.76333	0.11771
964.9268	0.90851	0.02775
991.56457	3.46057	0.09594
996.54863	3.76553	0.15024
999.28909	8.55209	0.26708
1012.6457	0.0096	0.03044
1015.84238	0.32614	0.08883
1034.01439	2.10849	0.06501
1042.58054	12.9304	0.2298
1046.98546	2.48533	0.09237
1047.10017	0.78801	0.01805
1054.81898	3.26876	0.0683
1072.83332	1.89082	0.10176
1075.32021	4.20629	0.09509
1079.29145	8.18389	0.25164
1091.45382	0.13233	0.10115
1091.69429	6.38734	0.08461

1105.20392	7.17274	0.07628
1123.88208	2.07958	0.14031
1130.11912	1.47192	0.05589
1133.70184	3.4479	0.08902
1146.57162	2.18042	0.12921
1154.27538	1.93319	0.07414
1181.06634	3.18286	0.03662
1181.77941	3.01839	0.05803
1182.8739	0.6738	0.06246
1204.17507	1.34345	0.06809
1247.0233	7.1924	0.12264
1250.43802	3.17138	0.07797
1265.31582	1.13028	0.02223
1265.68816	5.36596	0.11504
1270.4192	0.66614	0.05059
1274.13075	3.05963	0.06189
1279.67347	3.17136	0.05878
1283.24455	7.11787	0.1398
1298.03707	3.49134	0.06187
1300.36329	1.11413	0.02036
1305.24203	5.72603	0.12133
1311.99222	3.46994	0.05824
1326.60888	1.00559	0.05029
1331.16803	1.30476	0.05555
1334.61926	3.98522	0.08762
1342.88169	0.60849	0.04731
1351.11251	2.13198	0.06117
1354.90428	1.52519	0.04753
1363.65907	3.36736	0.08548
1367.45477	0.79266	0.0419
1372.28678	0.28345	0.02201
1374.35562	2.72882	0.05633
1376.85905	0.03549	0.01671
1381.98085	0.0108	0.03355
1389.33203	1.6904	0.06599
1392.88624	1.53215	0.0519
1397.57374	2.11596	0.08478
1399.65352	6.58364	0.19926
1404.50612	2.50819	0.06286
1409.32176	6.23506	0.11545
1416.79577	3.53106	0.11658
1421.70053	6.56689	0.15003
1427.55875	3.913	0.10226
1428.94336	7.67953	0.19537
1446.63279	0.14963	0.04891
1447.49158	2.71341	0.08361

1474.7591	1.6329	0.08196
1476.36862	1.27607	0.11629
1481.29328	0.00617	0.00907
1485.30076	3.45228	0.06783
1486.70741	5.39096	0.04869
1488.63174	2.40001	0.04893
1489.90323	0.91313	0.03731
1492.09305	5.38745	0.08528
1492.82082	1.7066	0.03542
1494.18982	1.23721	0.06888
1496.13259	1.64285	0.06449
1497.56353	2.06554	0.05285
1498.47899	1.6123	0.05956
1501.04985	2.80223	0.0426
1503.32656	0.42335	0.05976
1503.95437	0.29524	0.0241
1506.67298	1.19473	0.0354
1509.35776	0.59774	0.05807
1511.42906	0.88318	0.02844
1523.21388	1.85691	0.09423
1534.38801	15.60405	0.31409
1540.77252	25.74372	0.67538
2859.63296	29.48961	0.59354
2867.02158	21.02731	0.55361
2970.94896	9.57436	0.13174
2985.70266	9.82661	0.12898
3027.54076	1.01558	0.02378
3027.86559	1.22991	0.03085
3042.68372	1.23159	0.02479
3043.53495	0.68122	0.02008
3050.04135	0.50377	0.01424
3052.24415	2.61582	0.14834
3058.59905	1.02784	0.02586
3061.19456	0.09283	0.01285
3061.77137	0.02345	0.00857
3067.97985	1.27682	0.03566
3069.02004	0.54649	0.02135
3073.61696	0.50064	0.00914
3075.34858	0.1426	0.01405
3077.45893	1.91835	0.04571
3080.3968	1.13195	0.0263
3083.85163	0.27642	0.01427
3087.86749	0.56888	0.01491
3088.83799	1.02371	0.02115
3094.78187	1.81418	0.02435
3096.14434	0.9672	0.0924

3098.61737	3.26961	0.05859
3100.81072	0.67222	0.01747
3101.08431	1.72735	0.04569
3105.14868	3.39458	0.07346
3106.8691	0.16371	0.0275
3108.32533	1.44052	0.0297
3110.67918	6.83968	0.14063
3118.01756	3.7364	0.0623
3119.57782	2.39326	0.07669
3130.70936	0.50123	0.00611
3143.1527	0.17938	0.03046
3144.46427	0.02104	0.0513
3150.27335	0.05661	0.0246
3151.72581	0.23147	0.02741

Table S16: List of spin phonon coupling coefficients for 5

Frequency (cm^{-1})	$\left|\partial U_{eff}^{TI}/\partial q_\alpha\right|$	$\left|\partial B_m^l/\partial q_\alpha\right|$
71.63418	88.99999	0.87245
92.67997	66.3479	1.19046
102.71128	96.03363	1.97185
109.33254	8.18792	0.28548
129.18172	10.62035	0.28393
133.71715	74.24865	0.62949
150.41486	20.28105	0.06143
157.15492	19.9266	1.02057
163.46091	46.86827	0.12181
171.56154	176.21266	1.18
187.37694	4.89389	0.16342
190.10642	149.28987	0.97447
193.66834	35.31671	0.16158
205.87135	18.25968	0.11057
213.62393	114.06779	0.87773
232.55807	9.5296	0.16837
235.41623	78.65156	1.22225
256.34723	8.99184	0.34004
263.07037	37.28837	0.24957
266.88164	8.38024	0.12887
277.15325	6.93766	0.11013
280.5573	28.00963	0.37234
284.80568	12.76171	0.11487
289.43733	24.86633	0.45312
313.94906	2.68482	0.08202
319.15278	14.50853	0.2496
329.64475	45.25637	0.77849
337.58093	5.78298	0.12248
343.95411	52.23823	0.24128
359.86588	16.32583	0.58341
373.78421	4.91373	0.29607
375.18173	0.3671	0.08556
394.63785	6.0015E-4	0.19535
396.1342	3.00434	0.04924
398.96837	7.9134	0.39398
410.49671	10.08441	0.12055
432.61088	1.88651	0.08966
443.62784	5.73962	0.1725
497.00403	3.28929	0.13627
507.2622	9.05204	0.14631
510.56937	5.26926	0.16125
519.28913	17.55357	0.22257
519.49093	25.03816	0.12161
532.072	12.56625	0.07316

556.33842	17.02202	0.09811
563.96067	7.9465	0.27945
593.72839	6.6564	0.06303
595.3219	1.69979	0.18597
613.81734	12.05711	0.12051
623.92136	25.8557	0.25965
663.94764	7.59078	0.33887
677.51292	25.7968	0.69884
695.73587	3.99939	0.2646
699.42624	1.91461	0.2429
719.27653	4.83954	0.44451
731.23999	20.19018	0.42726
740.31385	4.88516	0.14425
752.94195	26.39371	0.14995
756.41323	3.31287	0.01133
761.4635	13.49209	0.09071
787.70357	33.94063	0.21286
801.8398	6.17234	0.22386
805.27923	0.12646	0.18711
805.73352	10.05084	0.41816
811.09126	15.52644	0.10241
816.95279	10.24778	0.16831
836.85557	11.44924	0.06926
839.7388	17.32847	0.14356
867.95913	2.95256	0.05167
873.02844	5.27846	0.10875
907.75614	25.94053	0.26293
910.3248	0.89577	0.0816
928.50582	5.05904	0.03563
937.59931	14.25809	0.4653
938.2997	7.9243	0.17641
947.4603	3.66372	0.05873
952.14182	2.08646	0.15816
952.51829	7.57153	0.32667
960.23303	6.94235	0.04269
971.23386	16.4548	0.25297
985.54571	4.41699	0.04963
990.5917	24.16174	0.11074
996.377	9.82768	0.04327
1008.4122	2.11466	0.01902
1010.2168	6.58503	0.06086
1021.19418	12.56987	0.23356
1026.51781	0.80391	0.11995
1058.10219	7.20458	0.12702
1062.27212	6.49298	0.0352
1068.34179	14.67878	0.10083

1078.73468	6.93831	0.06885
1083.21233	3.89137	0.11035
1083.9443	0.20465	0.07188
1100.82595	3.27171	0.06318
1114.34378	3.95082	0.10706
1124.32652	9.73609	0.01638
1130.39519	8.47054	0.07609
1154.62557	14.97849	0.0688
1156.73219	6.91198	0.08636
1173.16369	1.82719	0.05317
1178.35559	5.42823	0.23514
1180.56312	0.73938	0.10295
1181.89271	0.99493	0.09459
1201.33708	3.40785	0.01666
1239.97934	2.9002	0.05311
1242.05115	5.28057	0.0711
1252.88602	0.03076	0.01422
1258.87943	1.08832	0.04027
1267.4616	1.99515	0.04284
1270.66657	10.07954	0.03994
1271.37772	4.58223	0.06236
1273.43522	1.8762	0.01665
1273.62688	3.11283	0.06226
1275.58537	4.14861	0.0503
1293.48303	1.54418	0.03156
1294.05936	3.57187	0.04668
1299.72628	3.64503	0.02129
1306.3498	0.01297	0.10439
1306.60482	14.92961	0.09586
1309.91926	13.83093	0.09512
1321.25248	5.25688	0.09832
1333.68171	5.99099	0.06769
1336.06192	0.50946	0.03857
1338.69415	0.51605	0.05828
1340.85083	5.19782	0.02364
1351.55871	5.69865	0.16469
1351.97871	3.37142	0.07596
1352.67921	8.99069	0.1326
1357.24083	2.23626	0.08817
1359.1418	0.0496	0.0134
1367.44112	4.67953	0.02383
1369.59754	4.20829	0.06987
1375.51355	0.2466	0.12814
1380.0943	2.76184	0.02081
1380.79567	0.17138	0.04735
1388.25521	0.30009	0.07197

1388.55789	0.98217	0.07708
1390.41444	0.96041	0.05854
1395.53522	5.17888	0.07634
1403.63118	5.6419	0.17935
1404.32928	0.06979	0.12947
1410.41098	3.39539	0.12529
1411.17266	3.6078	0.02969
1448.7802	5.37555	0.09995
1449.55251	6.61772	0.15597
1464.12873	1.82086	0.1028
1465.96366	4.21041	0.13245
1479.32179	1.94452	0.02186
1480.87239	1.3045	0.02456
1484.10693	3.35799	0.0272
1485.16222	1.41059	0.06072
1489.61215	3.76792	0.05311
1489.8472	0.36912	0.04579
1492.33663	3.41212	0.02756
1492.49185	6.14016	0.06189
1494.11071	0.87325	0.02553
1494.50523	0.65617	0.05929
1495.95061	1.04957	0.02568
1497.09151	2.21757	0.07355
1499.02633	6.82887	0.02757
1504.37274	11.2862	0.125
1506.25144	8.91757	0.05547
1509.38332	6.92235	0.05478
1512.55578	4.40407	0.01955
1525.88834	33.90926	0.30319
1561.25116	13.62147	0.08043
1562.4345	51.23897	0.46701
2975.81866	10.59851	0.02784
2980.40227	71.78542	0.73236
3032.7743	1.15092	0.01316
3033.17263	0.18194	0.03713
3034.74678	0.5735	0.01553
3034.75231	1.11612	0.00975
3046.53155	0.0849	0.01959
3046.6167	0.81858	0.02132
3061.63003	0.10907	0.00408
3064.09629	0.75658	0.018
3064.33825	1.32754	0.011
3064.60169	2.67345	0.02559
3064.95131	1.51421	0.02519
3072.94636	11.00574	0.02955
3073.83471	7.85389	0.04192

3074.19309	6.99313	0.02754
3075.90861	1.60791	0.02407
3079.28399	0.22914	0.01457
3079.84454	1.20688	0.02443
3084.03418	1.84983	0.05064
3084.20284	4.28502	0.07182
3089.70215	26.36652	0.21112
3090.75553	1.77713	0.01091
3091.05033	0.59613	0.01682
3091.98501	0.51707	0.00624
3092.14018	0.43893	0.00734
3104.05546	9.3704	0.04572
3107.47262	3.91751	0.05396
3107.82576	7.31795	0.08737
3109.88551	16.82478	0.1912
3115.38257	4.15862	0.09316
3115.90196	19.2871	0.20229
3120.11685	5.73972	0.03573
3120.91746	2.59777	0.01886
3122.02939	0.85264	0.01458
3122.98502	7.47907	0.04179
3131.23802	0.43918	0.00914
3131.86368	5.19846	0.09104
3158.56337	1.61145	0.03601
3159.42526	0.93882	0.00569

Table S17: List of spin phonon coupling coefficients for 6

Frequency (cm^{-1})	$\left|\partial U_{eff}^{TI}/\partial q_{\alpha}\right|$	$\left|\partial B_{m}^{l}/\partial q_{\alpha}\right|$
21.03112	37.55413	2.29271
31.48649	55.04524	11.6597
39.2821	93.64832	9.87409
43.83062	54.26968	2.88804
44.07878	5.45659	1.59259
48.40746	43.16629	1.06085
53.81945	1.73149	3.67787
64.09642	54.68418	6.34864
83.81486	1.28829	1.90865
88.83563	0.71107	0.4422
95.59856	6.09068	2.43374
104.34332	6.72171	1.76216
107.27702	0.15307	1.25613
110.65971	1.89707	2.08979
110.90764	17.80109	0.92574
118.58111	13.44521	0.62876
119.54193	11.99917	0.87409
125.83035	17.96157	1.60851
128.33733	7.17838	2.50458
137.50893	4.95411	1.76557
146.4483	0.16955	0.88706
152.13234	12.97537	1.59373
156.63393	6.48433	0.26718
157.9974	8.34557	1.31338
163.7297	2.96819	0.92435
170.67246	2.99713	0.98931
171.57923	8.92516	0.80206
185.84872	6.71695	2.43009
191.49387	4.36559	0.61216
197.28797	1.41578	2.49821
202.33428	0.19624	1.09974
204.90151	0.44585	0.56167
207.08054	7.80162	1.31848
213.53074	1.99675	1.03816
216.46232	3.19443	0.60774
218.13075	7.74618	0.49239
221.85914	8.12388	0.76712
222.57212	4.14287	0.79865
224.72412	5.72311	0.25594
231.65242	7.86702	1.33713
243.26285	3.65046	0.47457
252.84796	1.27895	0.79005
256.25989	6.71435	0.82736
267.668	3.77508	0.66912

270.24153	3.73178	0.74494
274.59644	0.68585	2.05411
275.87039	3.84633	0.30207
290.56871	6.40451	0.19837
293.06499	1.79688	0.74687
312.4968	7.70107	0.94961
331.32055	3.25885	1.86933
332.60566	22.60648	1.02841
370.94788	18.62861	0.47523
386.19435	4.25025	0.63735
388.2765	12.86959	0.48725
414.87652	1.93084	0.8036
416.07914	8.45571	0.40738
466.61346	14.41753	1.13635
467.73509	9.05888	0.58323
491.48789	2.77438	1.28522
508.42356	11.45464	0.62068
538.50432	0.17841	0.29696
541.83818	0.13745	0.51615
542.85741	2.21264	0.71508
548.62579	4.08909	0.47183
550.38433	2.21202	0.4803
562.835	7.44535	0.48076
563.88743	2.28867	0.91376
589.90303	0.8504	0.50484
623.30613	5.45294	0.38489
627.85013	3.14691	0.66171
723.17396	10.78704	0.37191
726.63762	1.56628	0.69282
731.21735	4.71772	0.57137
750.43373	3.01658	0.23786
751.02018	2.70057	0.71198
802.28817	3.48898	1.49049
803.73045	1.72119	0.34939
866.6827	3.64501	0.62354
874.16998	2.41933	0.31964
881.69317	6.67744	0.52521
908.75903	0.83581	1.09571
910.63906	2.74994	0.64742
914.68728	1.39235	0.30913
917.01376	0.166	0.26573
918.09772	1.88388	0.61773
921.66461	0.35935	0.33519
924.85478	0.52000	0.63924
949.7452	1.66376	0.29028
954.63647	0.06541	1.01129

955.94584	8.76987	0.74572
958.49386	0.07372	0.92673
960.66203	0.70598	0.42613
962.55149	1.99219	0.1472
964.24548	0.23912	0.26093
1011.90629	0.97091	0.32324
1018.88556	1.0896	0.44923
1029.59182	6.78496	0.26923
1032.05776	2.69298	0.24145
1036.15852	2.0127	1.24008
1040.34064	0.35396	0.46241
1042.39558	2.92819	0.25252
1076.05536	1.1518	0.6523
1076.60395	0.51348	0.31219
1095.67534	0.83703	0.20347
1097.90651	0.60453	0.82298
1114.41616	1.56624	0.46168
1115.48867	2.34073	0.31969
1120.53833	1.01827	0.19445
1121.48928	1.11629	0.59247
1128.42772	1.26875	0.14119
1130.13655	1.58871	0.56731
1130.74294	0.41048	0.38526
1132.46605	1.20683	0.49009
1134.85332	0.70369	0.20212
1160.6691	3.37686	0.24523
1163.85062	1.59606	0.50239
1172.31722	0.78493	0.11599
1183.72181	0.21814	0.37174
1184.35242	4.37958	0.83497
1200.75595	1.01504	0.38858
1205.58277	0.02811	0.34505
1318.42061	2.41511	0.64206
1324.59793	0.42977	0.25874
1332.06078	1.16414	0.25406
1335.71464	1.87371	0.2315
1343.51563	3.16015	1.52682
1346.30419	3.63603	0.75975
1346.78387	1.62259	0.47624
1353.80874	0.70884	0.44561
1354.92968	2.02542	0.30822
1357.45144	0.98912	0.32952
1394.99385	0.49111	0.54744
1395.54902	0.37609	0.16485
1396.80815	2.37658	0.9026
1400.65209	0.45829	0.58386

1403.61064	3.70193	0.77563
1405.84986	3.63925	0.91817
1406.36705	1.74973	0.94461
1407.37616	0.75526	0.20693
1408.23208	6.49407	1.39178
1415.50911	1.19085	0.3817
1419.38422	0.68487	0.24084
1420.89975	6.22647	1.39011
1422.0143	1.02076	0.53144
1422.90016	3.48521	0.78963
1423.94405	5.57886	0.59594
1425.14133	1.35865	0.24752
1428.00301	1.65515	0.27099
1429.62409	1.58324	0.59062
1430.04491	1.99838	0.20933
1434.32679	3.00032	0.19093
1440.22289	1.08261	0.5758
1456.04797	1.15408	0.98178
1458.96089	2.21704	0.23884
1470.84511	1.32396	0.39114
1474.96049	1.13119	0.45738
1483.96739	0.62766	0.34846
1486.00058	0.50209	0.3908
1486.70233	0.47249	0.4456
1490.98217	1.21302	0.34032
1492.01415	0.29334	0.54651
1496.37534	0.65823	0.25816
1497.46136	2.79559	0.45737
1498.1438	0.63144	0.2048
1499.95912	0.10938	0.17945
1500.37412	1.54064	0.56451
1506.0731	1.12017	0.25526
1506.65039	0.69427	0.68126
1507.09128	0.91239	0.31549
1507.26937	1.15367	0.20284
1508.34664	1.97014	0.7252
1508.63168	3.77216	0.33157
1511.92806	2.26059	0.24232
1514.7698	1.05872	0.58136
1515.59517	0.23822	0.71187
1515.99519	0.03678	0.37629
1516.86685	2.18943	0.20578
1516.94514	0.3382	0.58438
1517.99037	2.24794	0.21626
1518.38081	0.09147	0.14287
1520.06984	7.14284	0.17529

1521.09874	2.59484	0.26395
1522.32521	8.02653	0.97225
1523.30217	5.87182	0.94435
1524.12297	5.0686	0.7331
1529.15256	2.25525	0.21406
2946.30457	13.36358	0.60093
2949.25455	24.10797	0.50309
3016.20659	1.74021	0.23956
3019.87709	1.62204	0.45804
3037.00238	0.18564	0.4324
3041.42191	2.86707	0.26767
3041.91421	0.19207	0.45053
3043.0482	0.63563	0.34237
3043.59681	1.75259	0.38757
3044.17189	0.88892	0.19523
3058.34935	0.11413	0.28394
3059.21483	0.79738	0.39111
3059.35909	0.45241	0.23191
3060.50577	1.67172	0.39983
3060.71717	0.25902	0.11423
3081.01383	4.77529	0.45449
3085.42854	4.81362	0.56918
3094.12986	1.64254	0.28802
3096.25056	0.757	0.51494
3097.56471	1.22654	0.33791
3098.67836	0.46793	0.14748
3104.13122	1.05403	0.64423
3104.16745	1.24807	0.04521
3104.88732	0.64972	0.48701
3106.88141	0.774	0.30334
3107.39304	2.08516	0.22994
3107.83145	0.36651	0.28548
3110.94602	0.71943	0.15042
3112.42269	0.6089	0.40378
3113.23457	0.25915	0.21114
3129.0796	5.78159	0.5054
3132.77176	1.35619	0.36304
3133.00271	0.25582	0.42099
3136.25517	0.49871	0.17805
3136.36124	2.04112	0.20076
3137.49059	1.173	0.44618
3137.81767	1.14685	0.06991
3139.07474	0.64983	0.21925
3139.73637	0.38542	0.18589
3140.4802	1.54774	0.46242
3140.7372	0.60838	0.28887

3140.9929	0.58455	0.52521
3143.03371	0.95442	0.53478
3144.3905	1.23795	0.40945
3145.8723	0.44654	0.32496
3149.22818	0.69454	0.7438
3150.55363	0.44278	0.05752
3153.46568	1.11924	0.68544
3154.29129	0.12099	0.26957
3156.02272	0.53466	0.09787

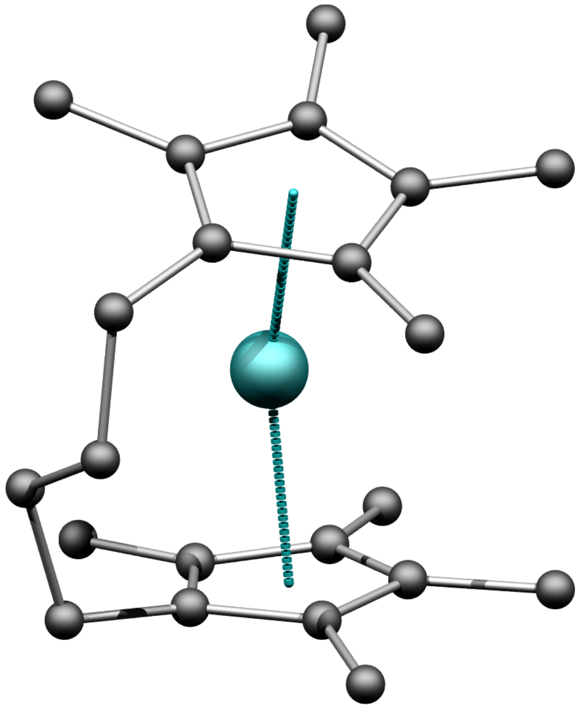

Figure S1: The computed molecular geometry of 1. The hydrogen atoms are not shown for clarity. The centroid-Dy-centroid contacts are depicted by dotted lines.

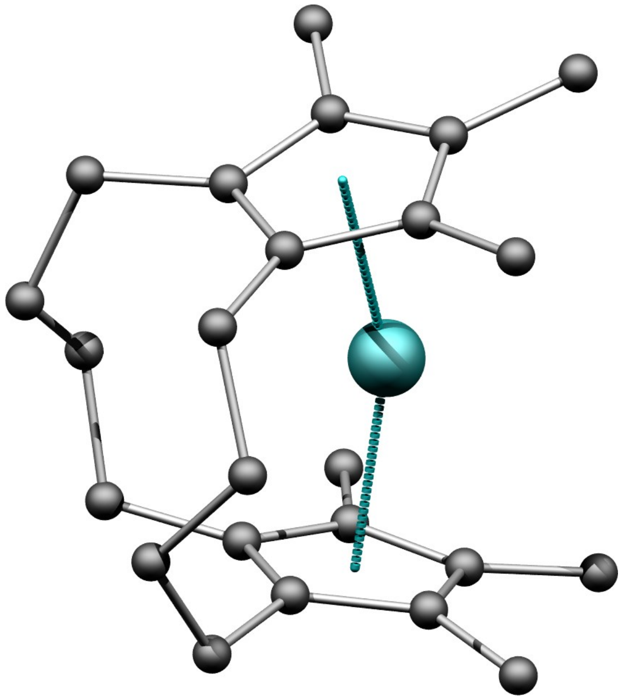

Figure S2: The computed molecular geometry of 2a. The hydrogen atoms are not shown for clarity. The centroid-Dy-centroid contacts are depicted by dotted lines**.**

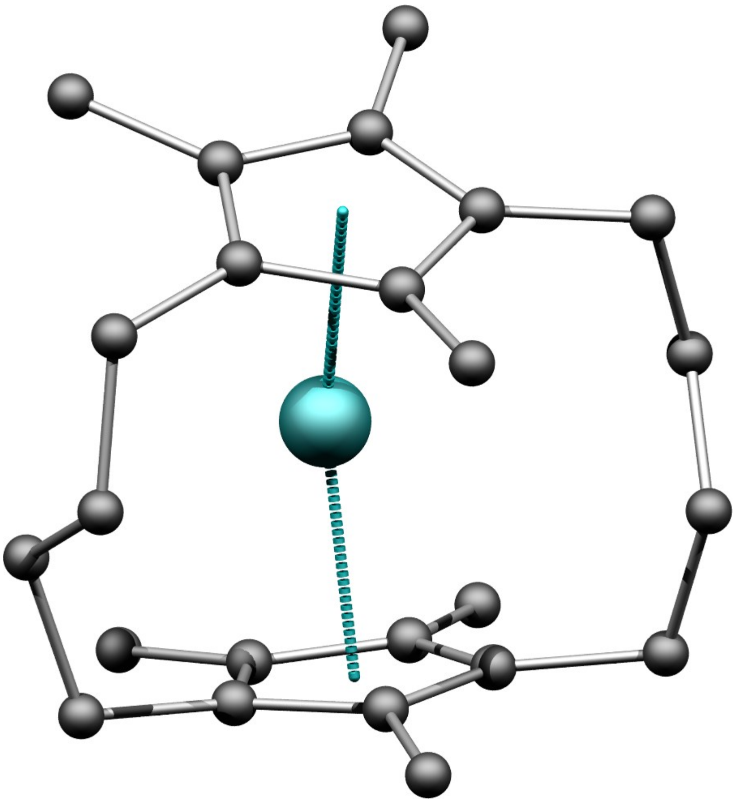

Figure S3: The computed molecular geometry of 2b. The hydrogen atoms are not shown for clarity. The centroid-Dy-centroid contacts are depicted by dotted lines.

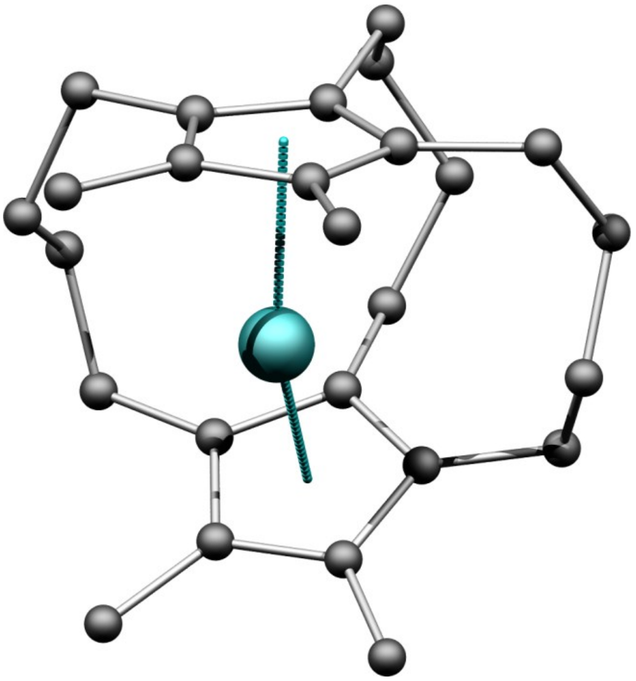

Figure S4: The computed molecular geometry of 3a. The hydrogen atoms are not shown for clarity. The centroid-Dy-centroid contacts are depicted by dotted lines.

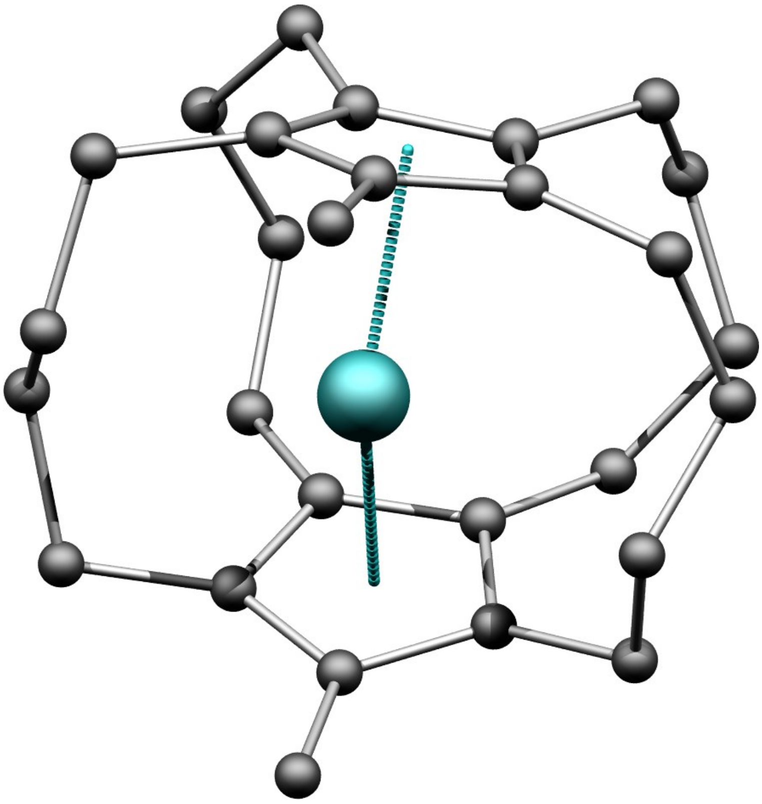

Figure S5: The computed molecular geometry of 4. The hydrogen atoms are not shown for clarity. The centroid-Dy-centroid contacts are depicted by dotted lines**.**

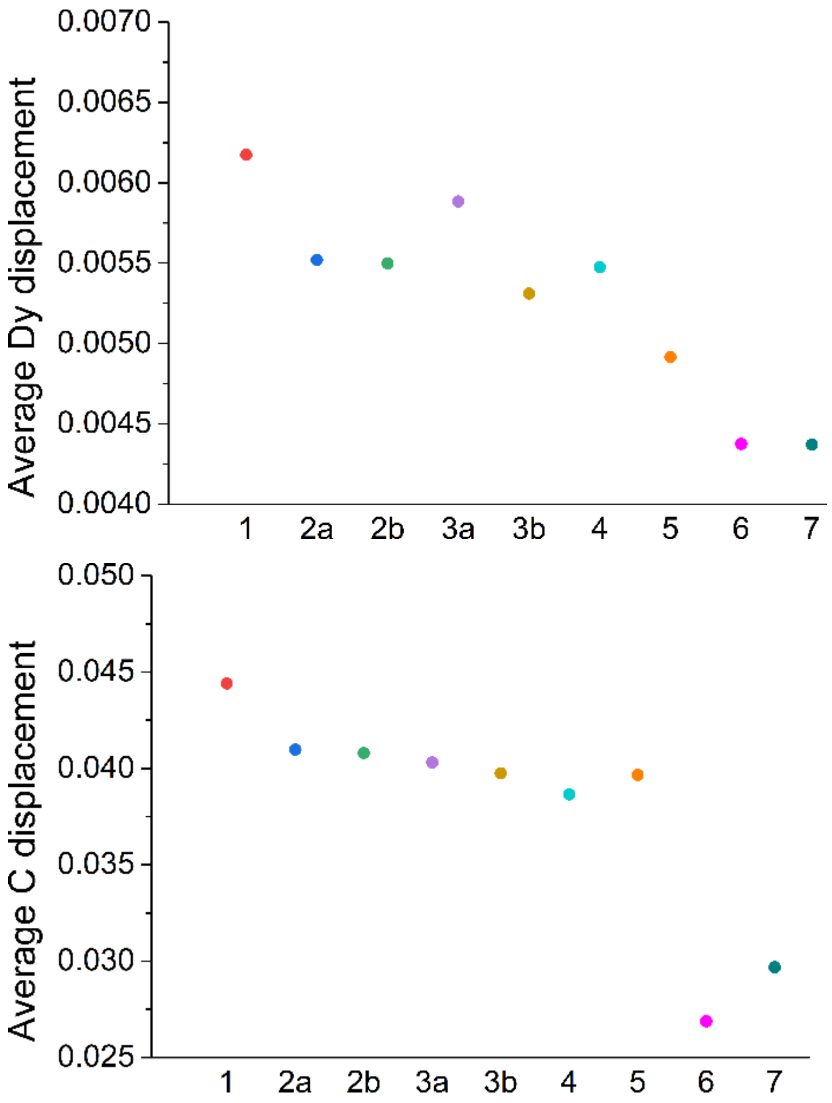

Figure S6: Sum of all (3N-6) vibrational displacement vectors for selected atoms, used to quantify rigidity of studied complexes. Selected atoms were central Dy atom, and ten carbon atoms, connected directly to Dy. Sum of displacement is divided by number of vibrations for respected complex, and also by number of atoms used for calculation (1 for Dy, 10 for C).

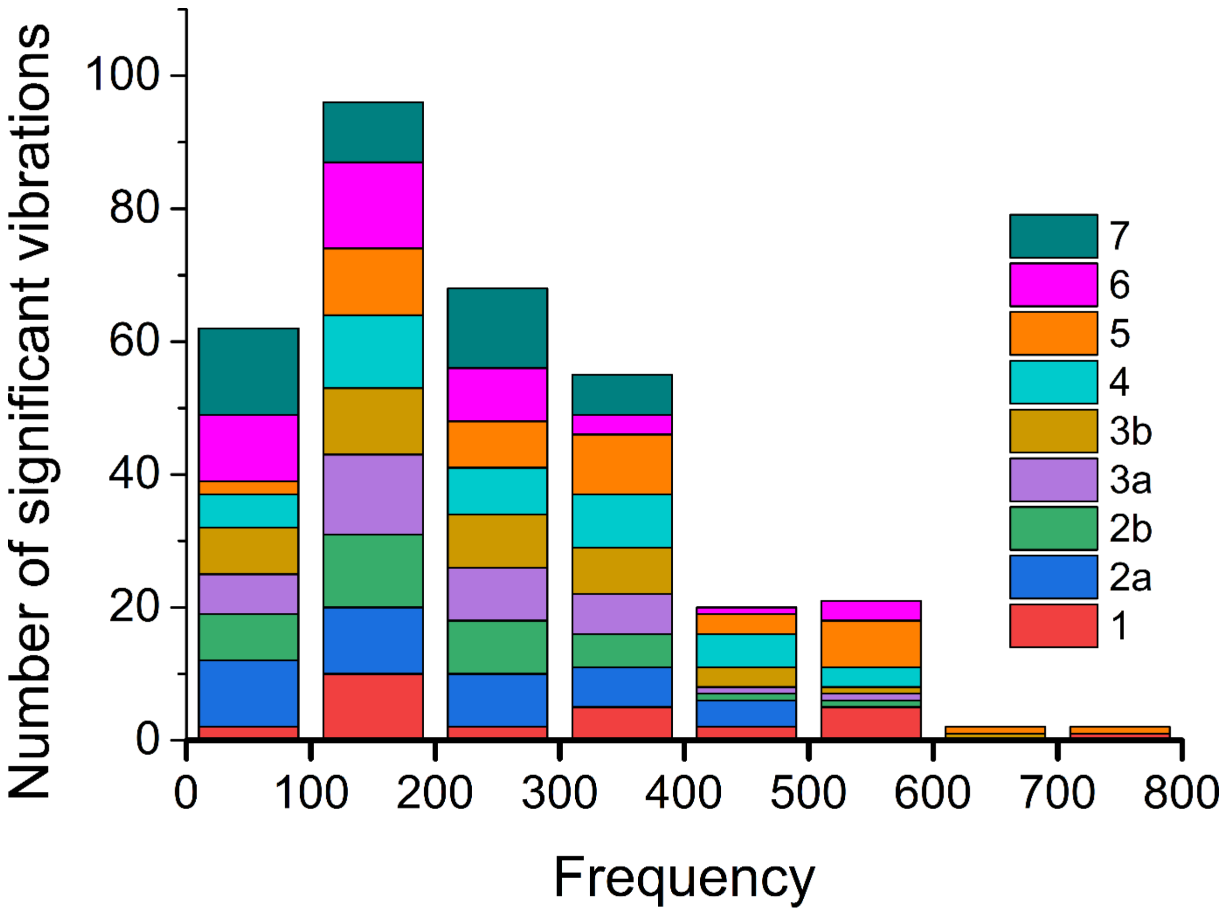

Figure S7: Comparison of numbers of significant vibrations, and their positions on energy scale. Significant vibrations are selected as those, which has displacement vector of Dy atom larger than 0.005 Å

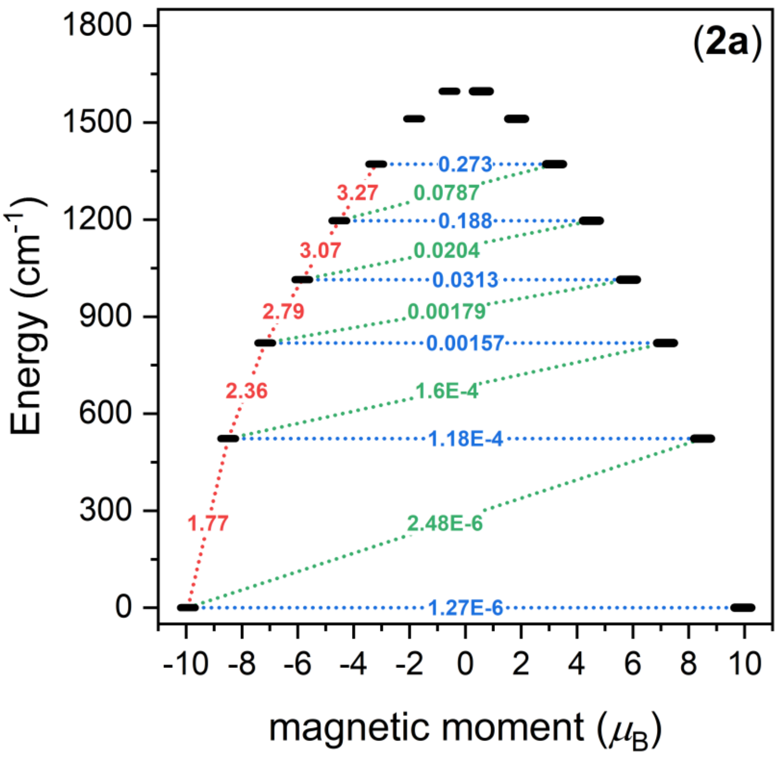

Figure S8: Visualization of *ab initio* magnetization blocking barrier for complex 2a

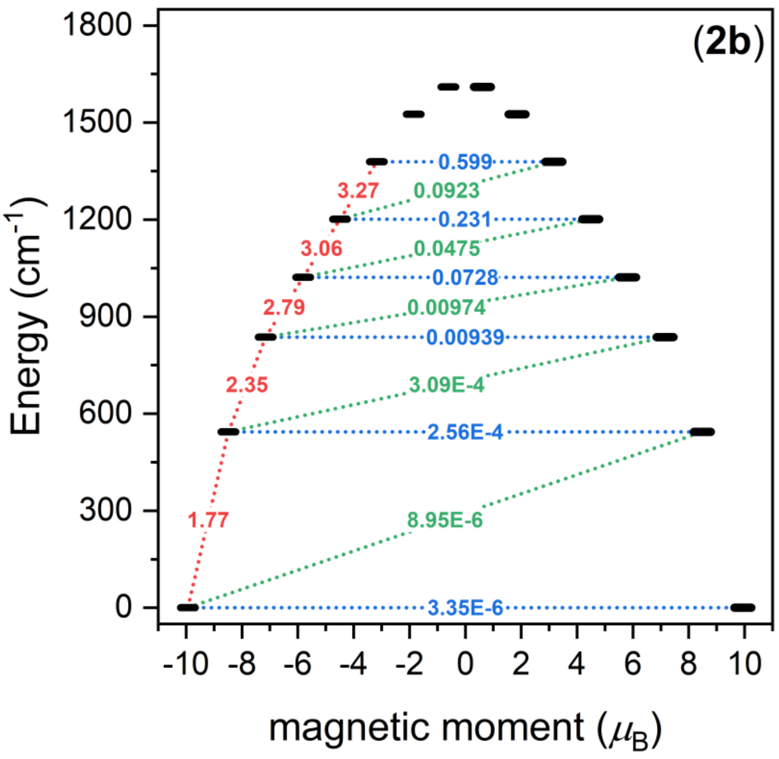

Figure S9: Visualization of *ab initio* magnetization blocking barrier for complex 2b

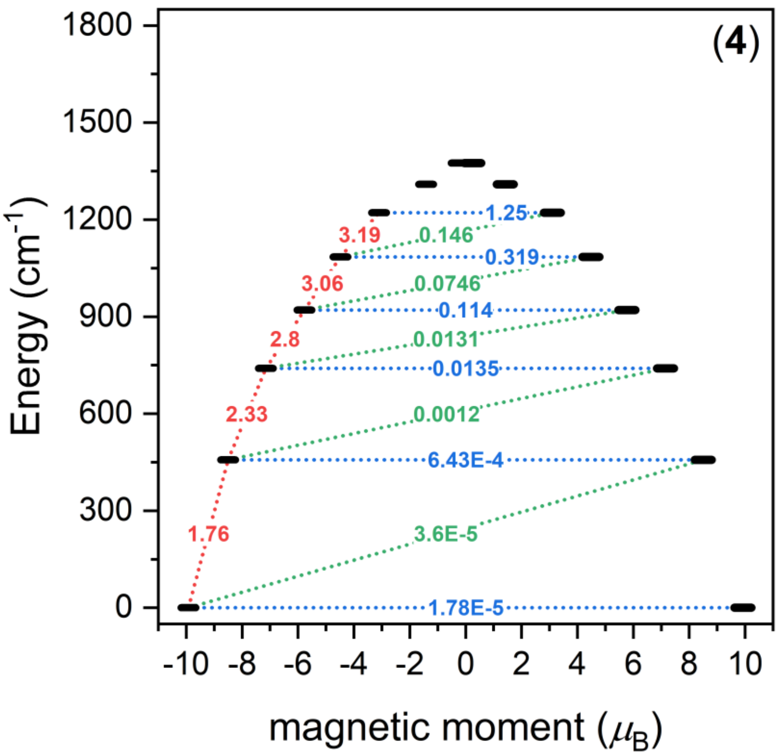

Figure S10: Visualization of *ab initio* magnetization blocking barrier for complex 4

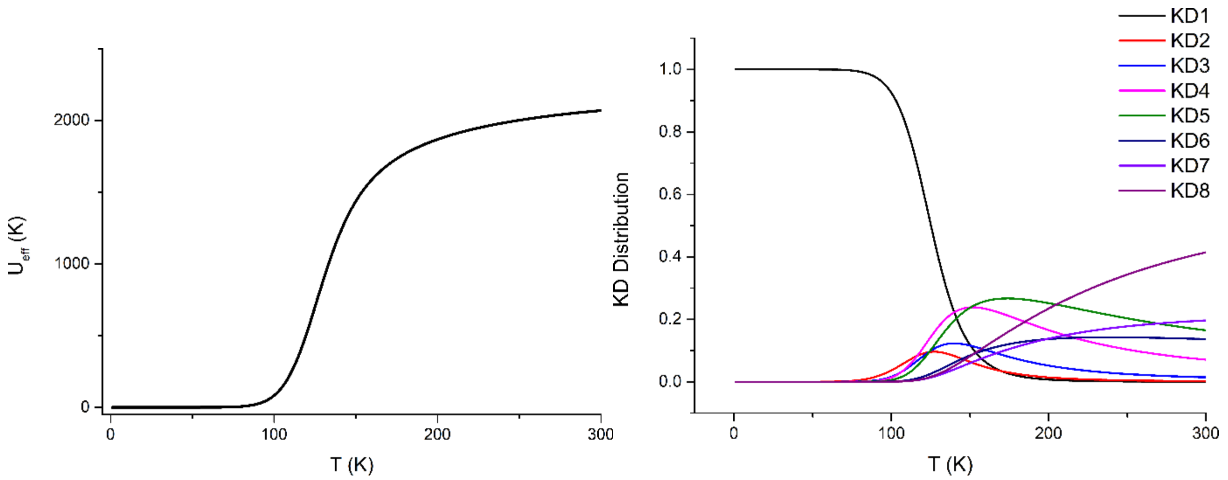

Figure S11: Temperature dependence of calculated U_{eff} (left), and relative contribution of each Kramers doublet to the relaxation calculated as $k_i(T)/N_k$ (right) for **1**

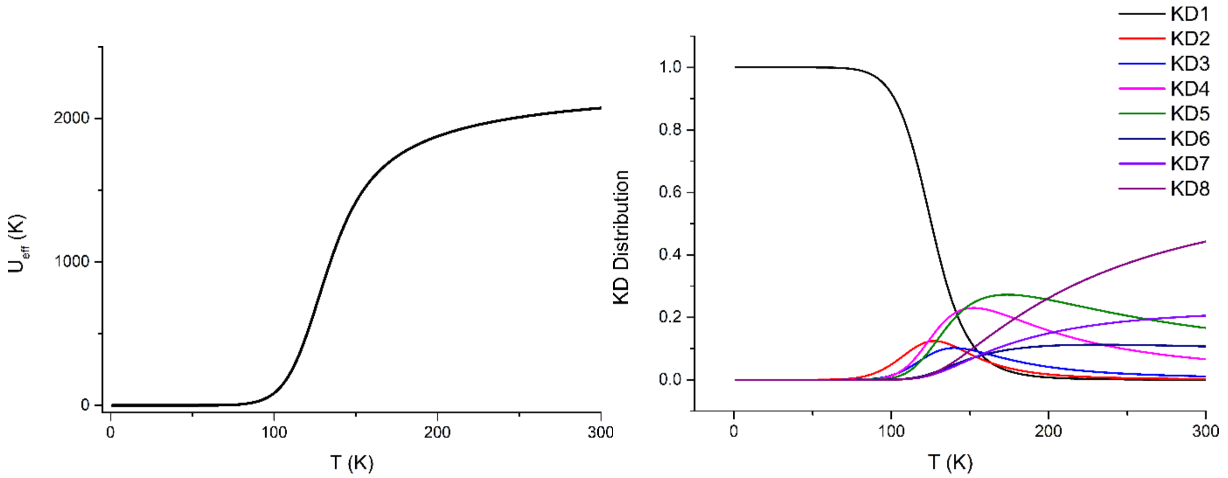

Figure S12: Temperature dependence of calculated Ueff (left), and relative contribution of each Kramers doublet to the relaxation calculated as ki(T)/Nk (right) for 2a

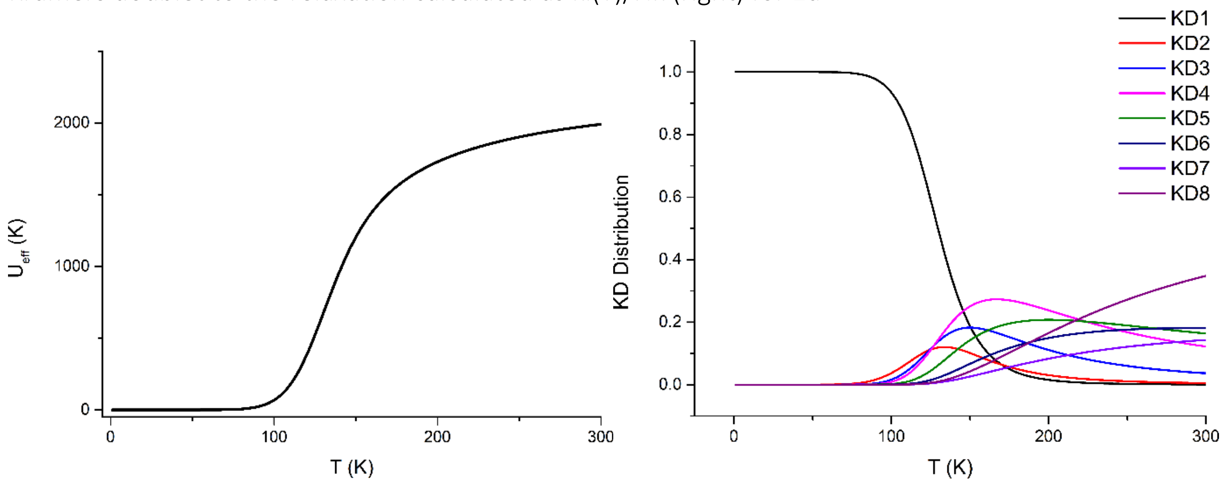

Figure S13: Temperature dependence of calculated U_{eff} (left), and relative contribution of each Kramers doublet to the relaxation calculated as $k_i(T)/N_k$ (right) for **2b**

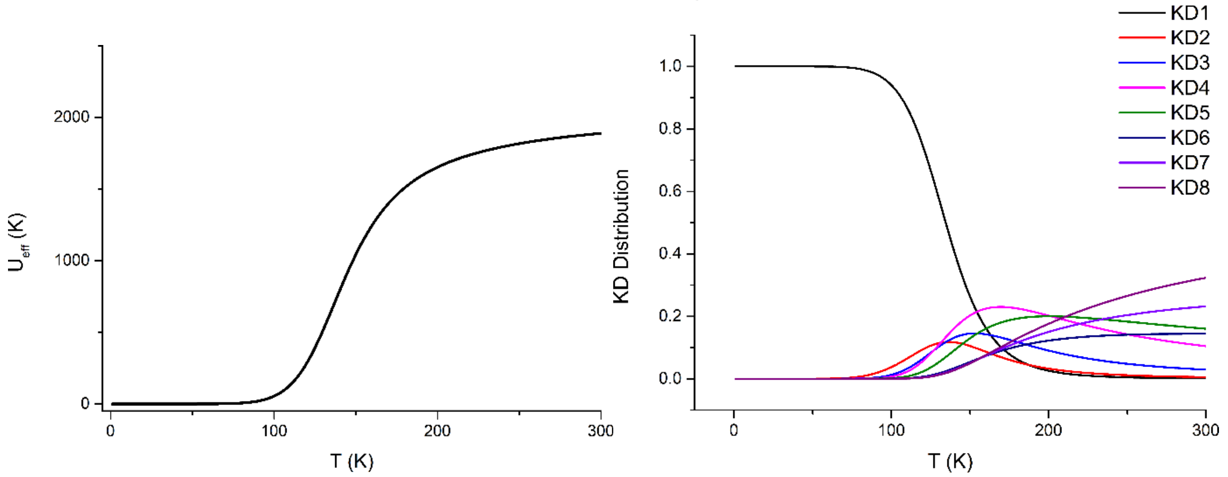

Figure S14: Temperature dependence of calculated U_{eff} (left), and relative contribution of each Kramers doublet to the relaxation calculated as $k_i(T)/N_k$ (right) for **3a**

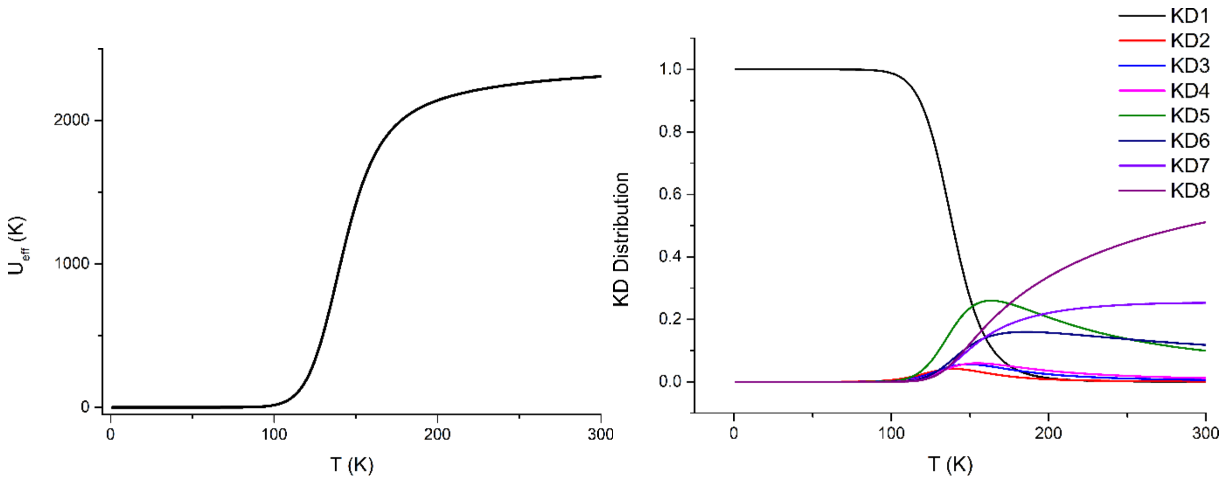

Figure S15: Temperature dependence of calculated U_{eff} (left), and relative contribution of each Kramers doublet to the relaxation calculated as $k_i(T)/N_k$ (right) for **3b**

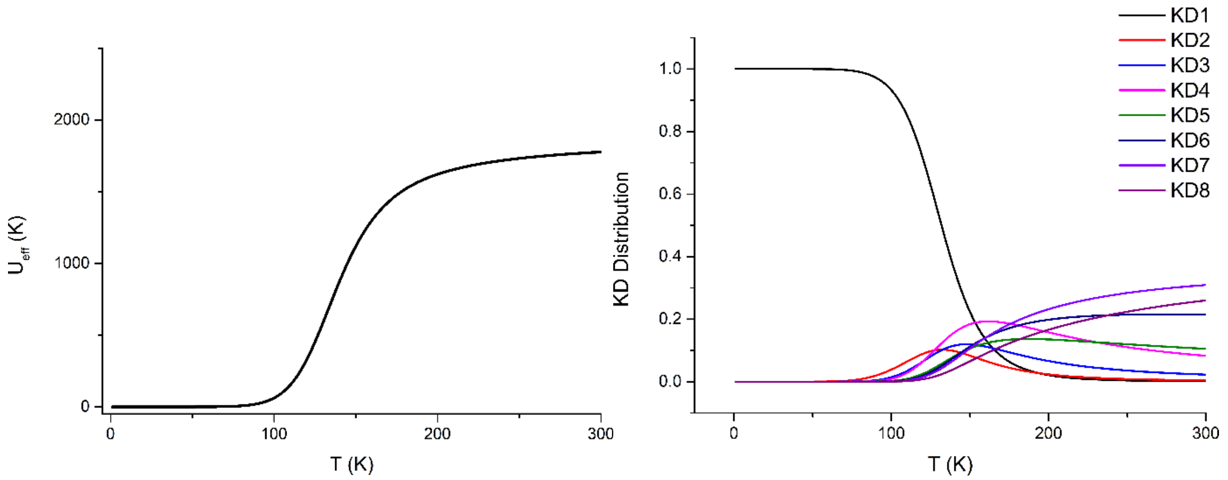

Figure S16: Temperature dependence of calculated U_{eff} (left), and relative contribution of each Kramers doublet to the relaxation calculated as $k_i(T)/N_k$ (right) for **4**

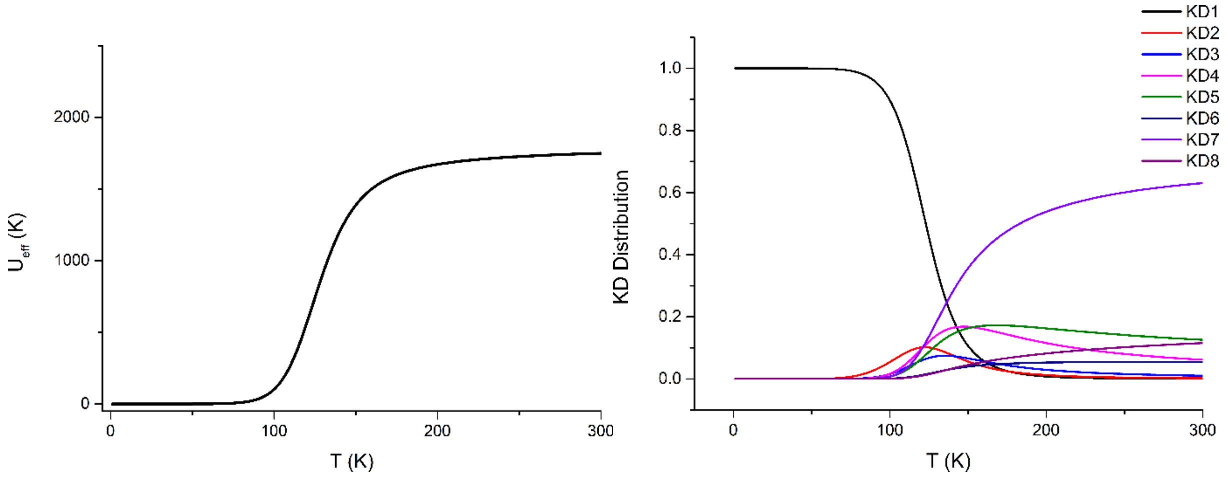

Figure S17: Temperature dependence of calculated U_{eff} (left), and relative contribution of each Kramers doublet to the relaxation calculated as $k_i(T)/N_k$ (right) for **5**

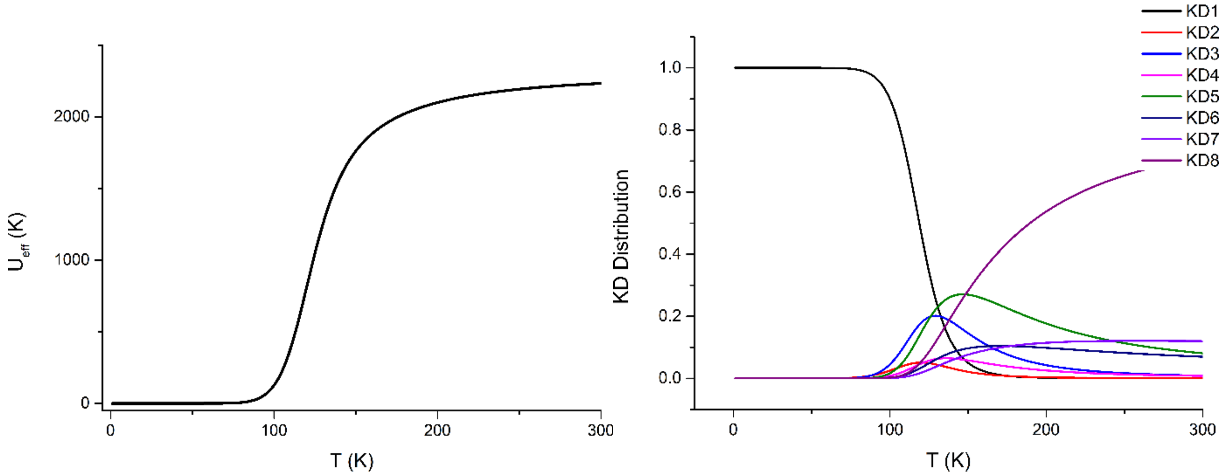

Figure S18: Temperature dependence of calculated U_{eff} (left), and relative contribution of each Kramers doublet to the relaxation calculated as $k_i(T)/N_k$ (right) for **6**

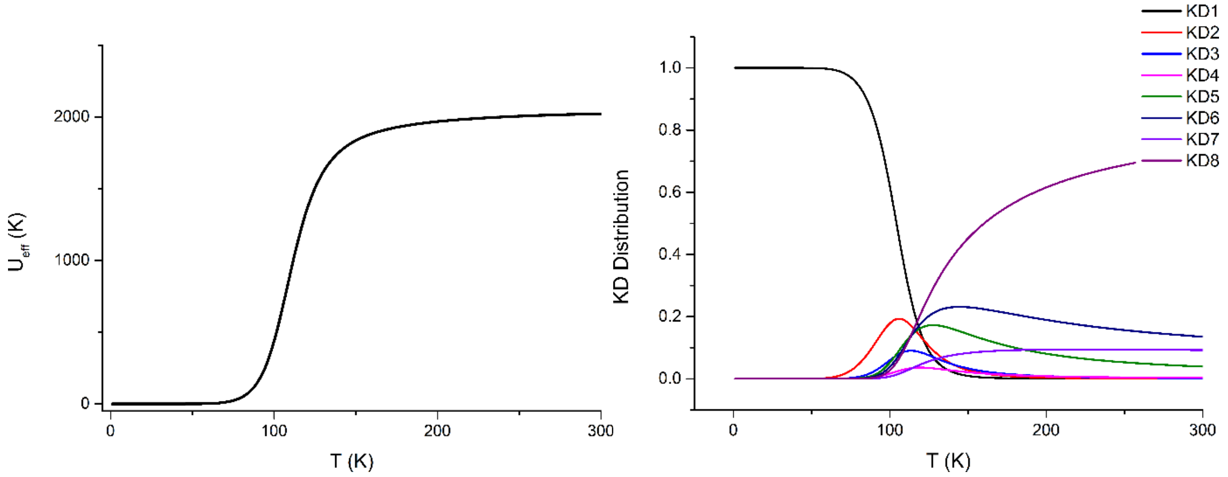

Figure S19: Temperature dependence of calculated U_{eff} (left), and relative contribution of each Kramers doublet to the relaxation calculated as $k_i(T)/N_k$ (right) for **7**

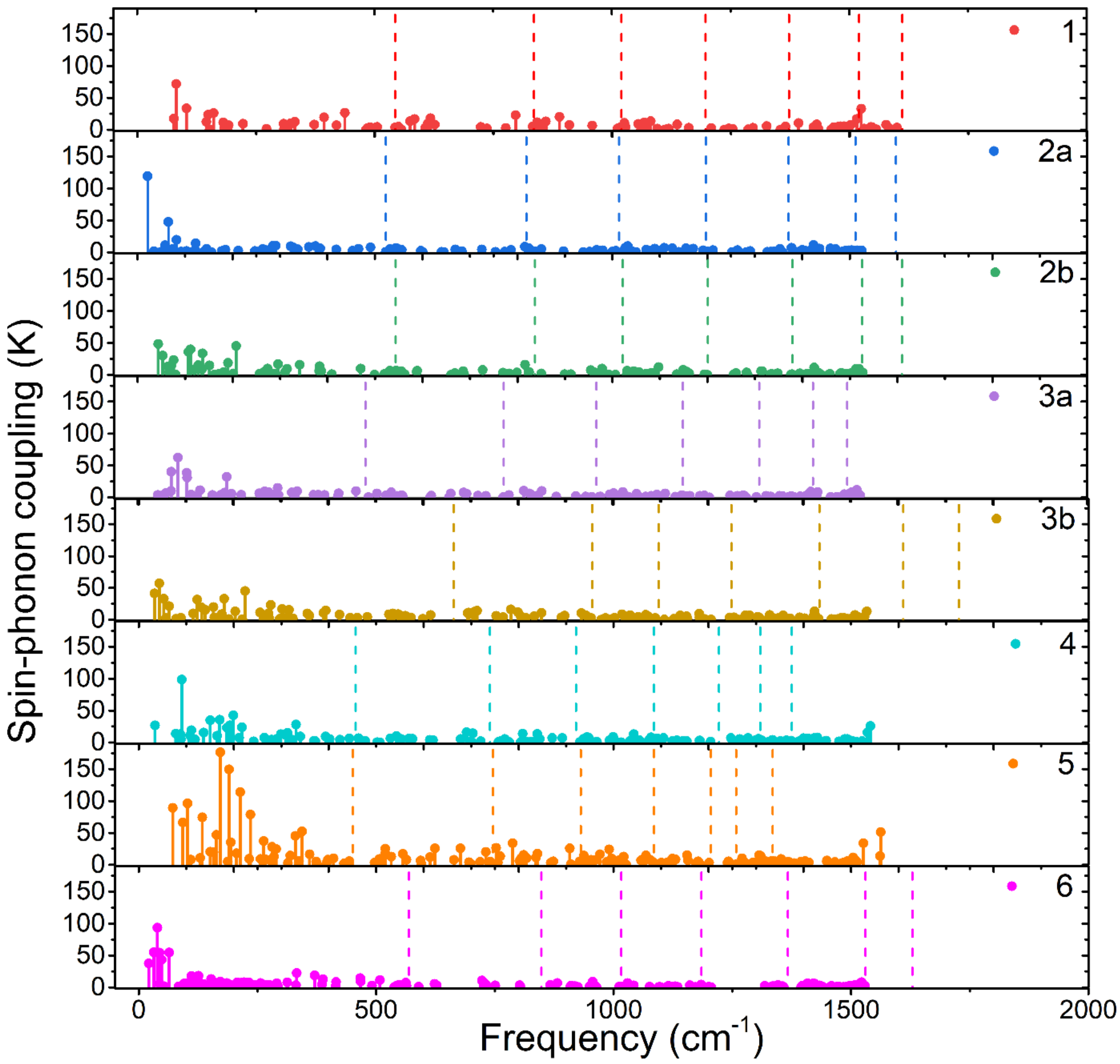

Figure S20: Spin-phonon coupling spectrum of $\left|\partial U_{\text{eff}}^{\text{TI}}/\partial q_{\alpha}\right|$ with shown energies of Kramers doublets transitions, marking their overlap with vibrations.

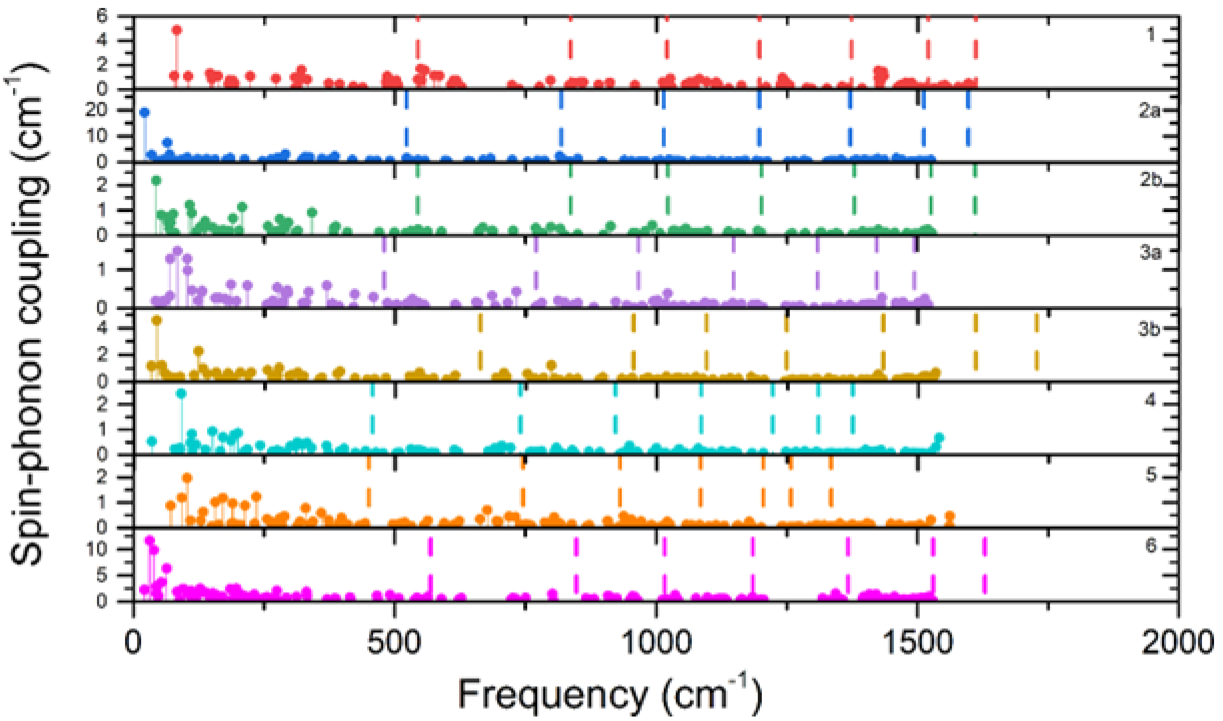

Figure S21: Spin-phonon coupling spectrum of $\left|\partial B_m^l/\partial q_\alpha\right|$ with shown energies of Kramers doublets transitions, marking their overlap with vibrations

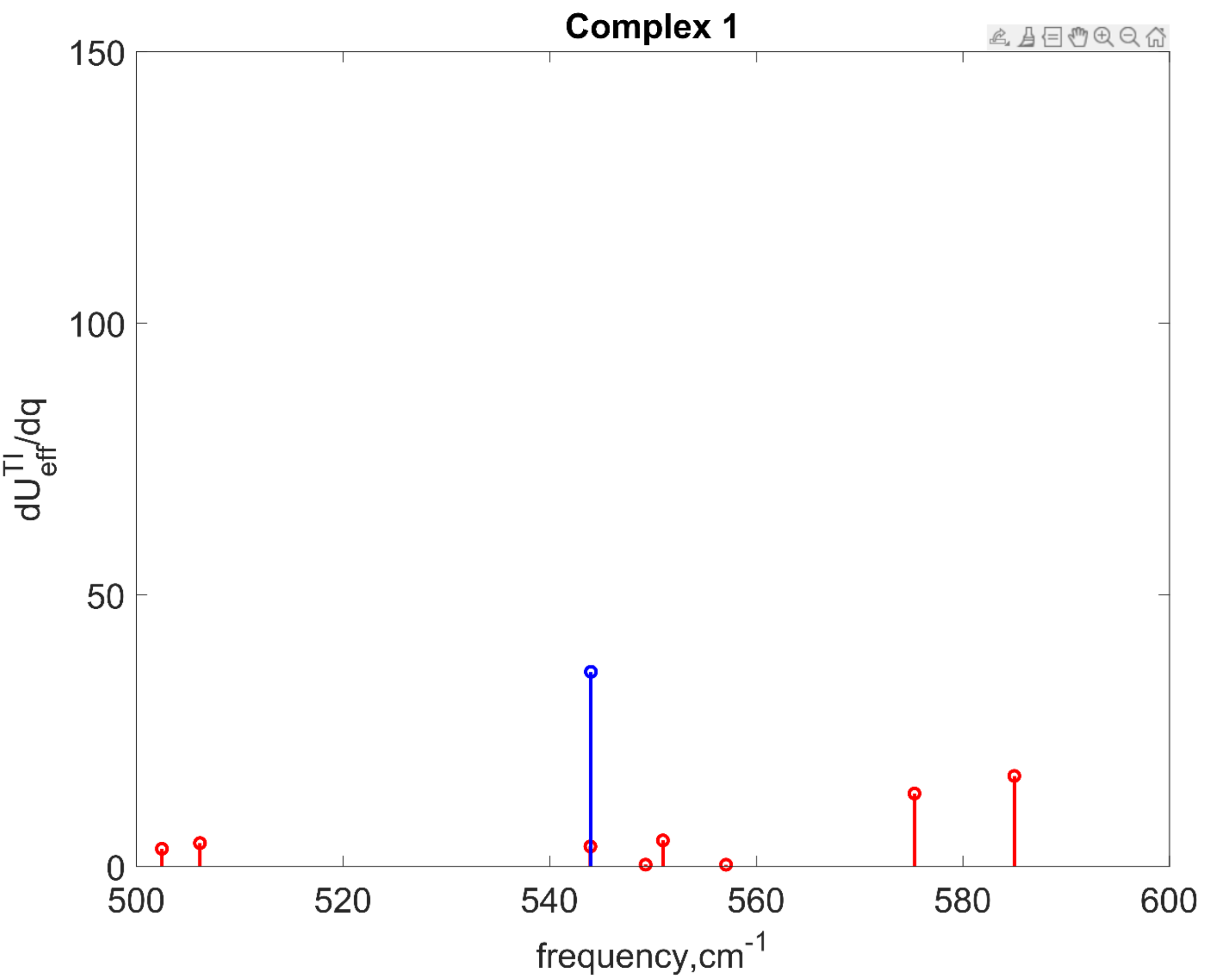

Figure S22. Spin-phonon coupling parameters $\partial U_{eff}^{TI} / \partial q_{\alpha}$ of vibrational frequencies in the vicinity of the transition (in red) from the ground to the lowest excited Kramers doublet (in blue) for complex 1.

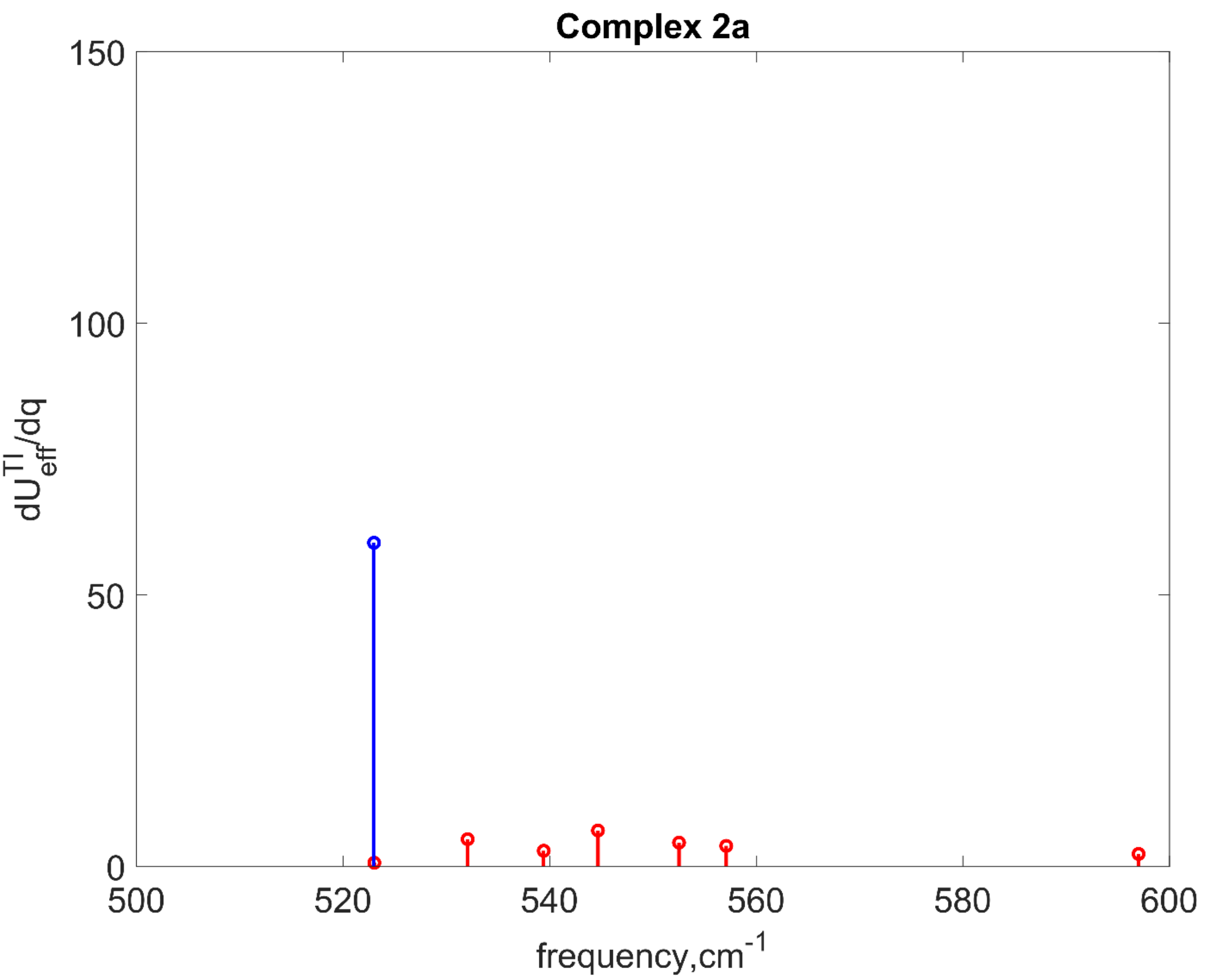

Figure S23. Spin-phonon coupling parameters $\partial U^{TI}_{eff} / \partial q_\alpha$ of vibrational frequencies in the vicinity of the transition (in red) from the ground to the lowest excited Kramers doublet (in blue) for complex **2a**.

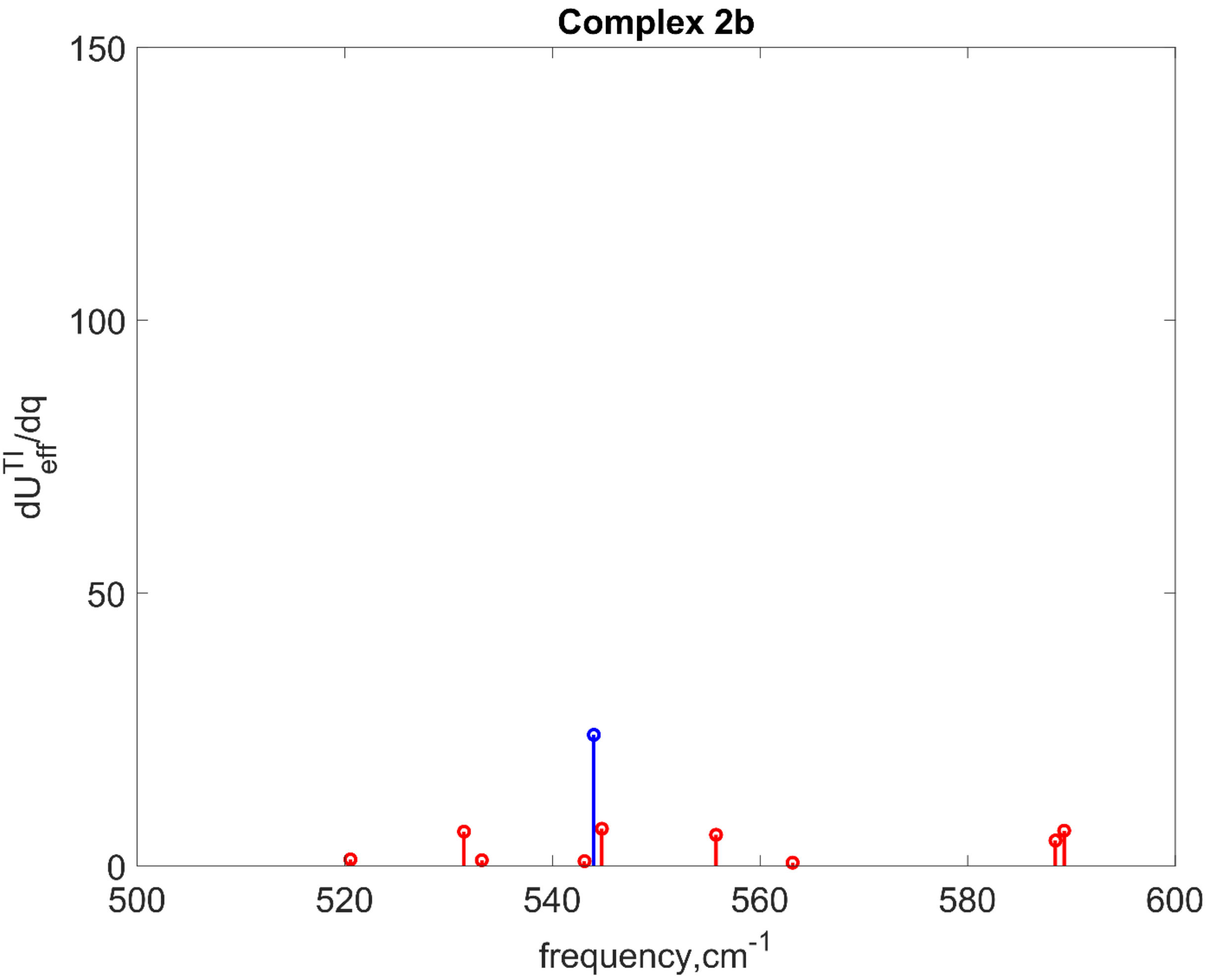

Figure S24. Spin-phonon coupling parameters $\partial U_{eff}^{TI} / \partial q_{\alpha}$ of vibrational frequencies in the vicinity of the transition (in red) from the ground to the lowest excited Kramers doublet (in blue) for complex 2b.

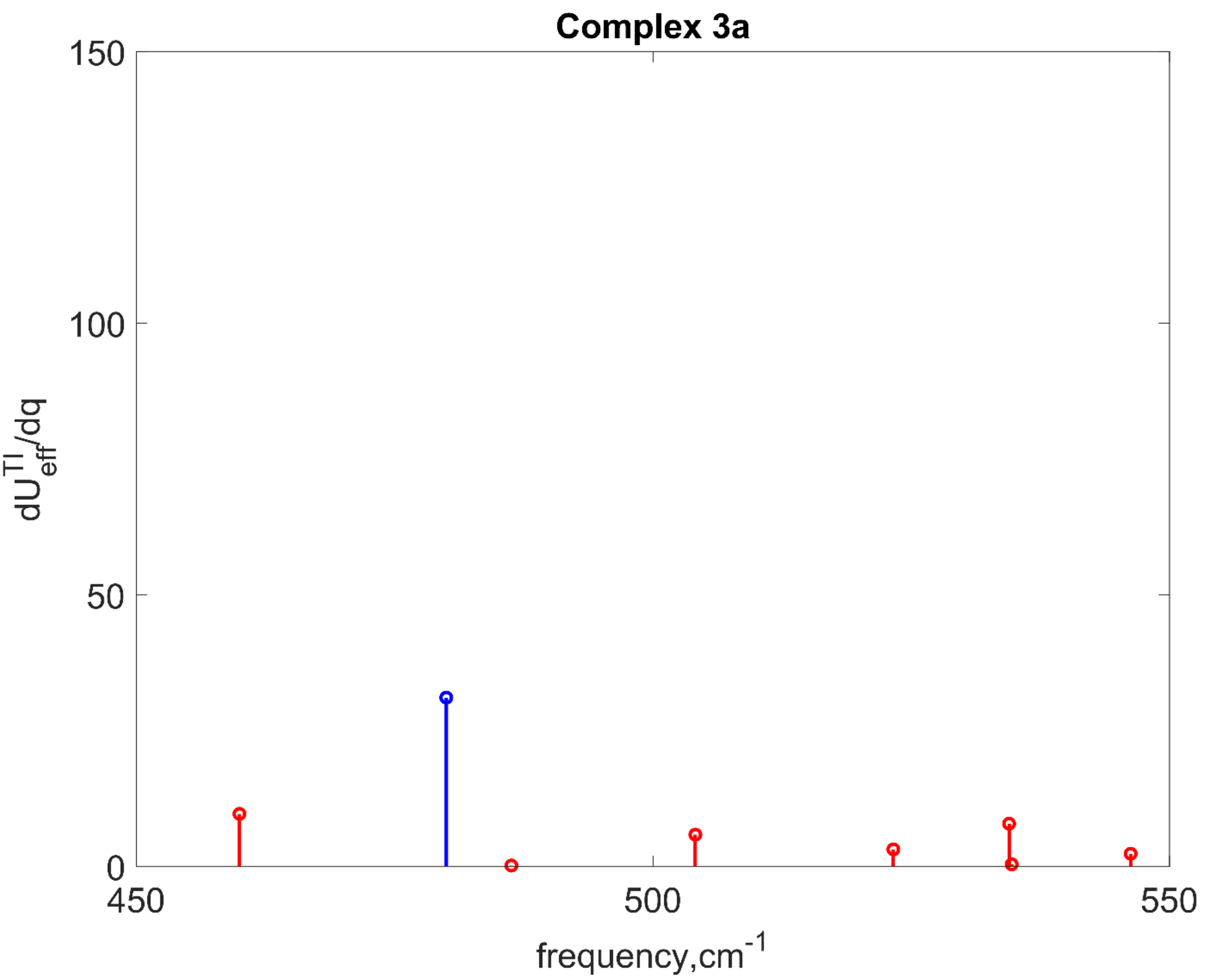

Figure S25. Spin-phonon coupling parameters $\partial U_{eff}^{TI} / \partial q_{\alpha}$ of vibrational frequencies in the vicinity of the transition (in red) from the ground to the lowest excited Kramers doublet (in blue) for complex 3a.

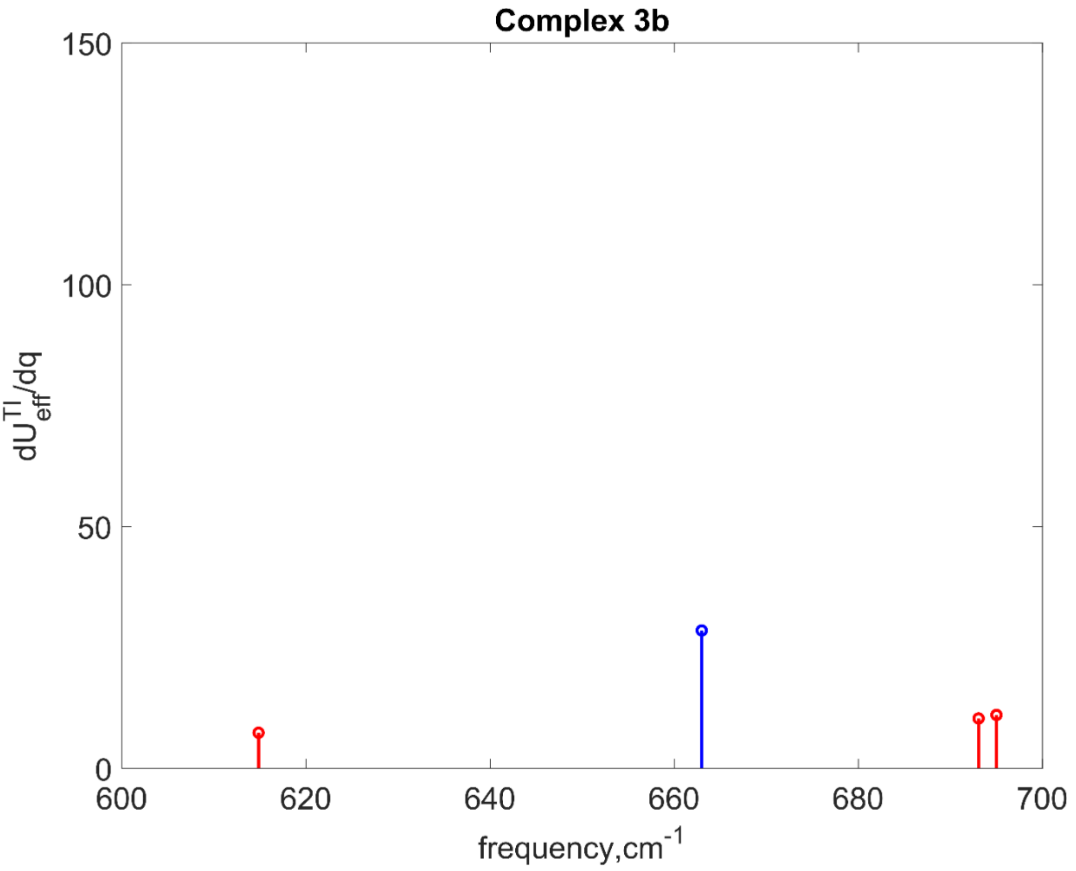

Figure S26. Spin-phonon coupling parameters $\partial U_{eff}^{TI} / \partial q_{\alpha}$ of vibrational frequencies in the vicinity of the transition (in red) from the ground to the lowest excited Kramers doublet (in blue) for complex **3b.**

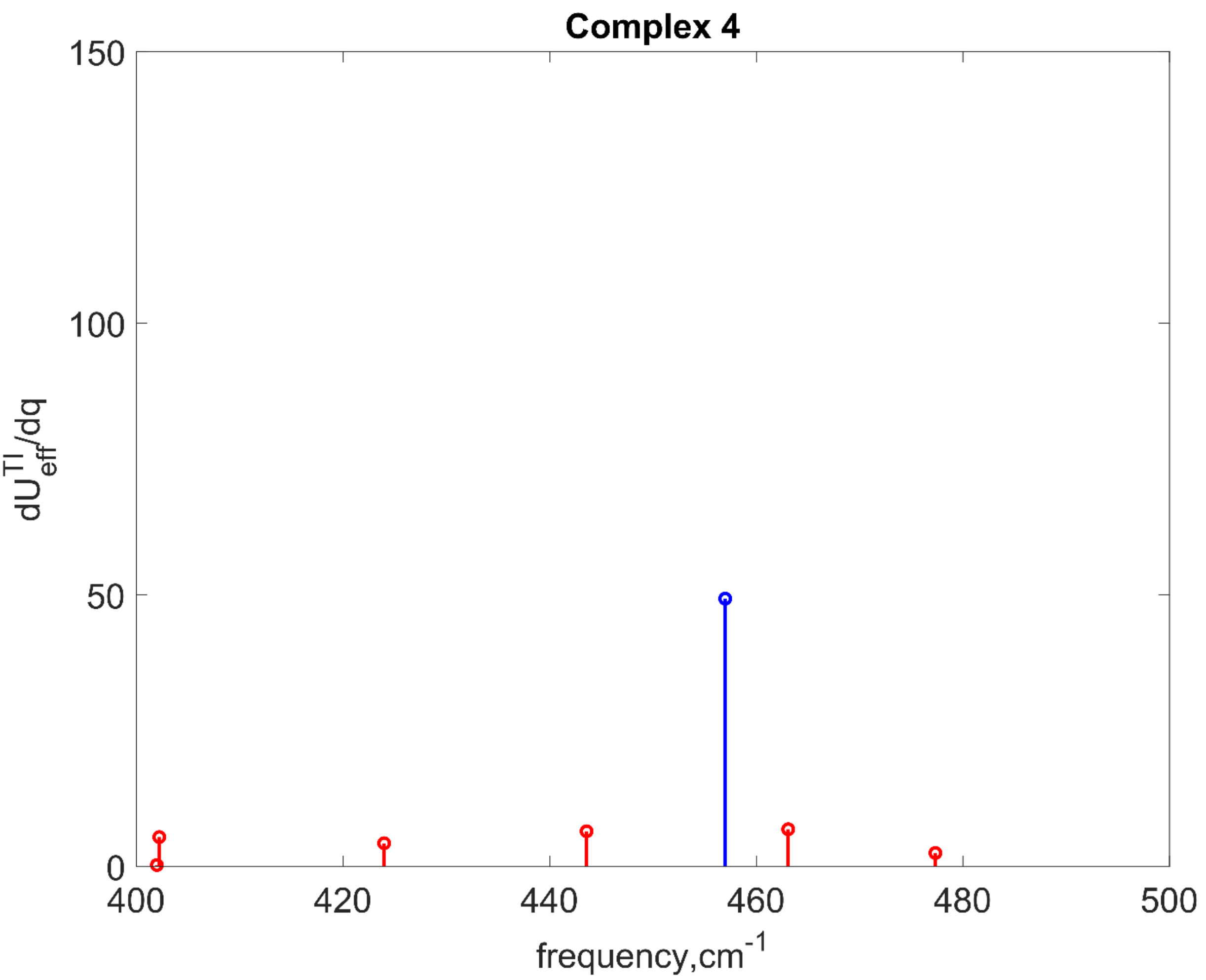

Figure S27. Spin-phonon coupling parameters $\partial U_{eff}^{TI} / \partial q_{\alpha}$ of vibrational frequencies in the vicinity of the transition (in red) from the ground to the lowest excited Kramers doublet (in blue) for complex 4.

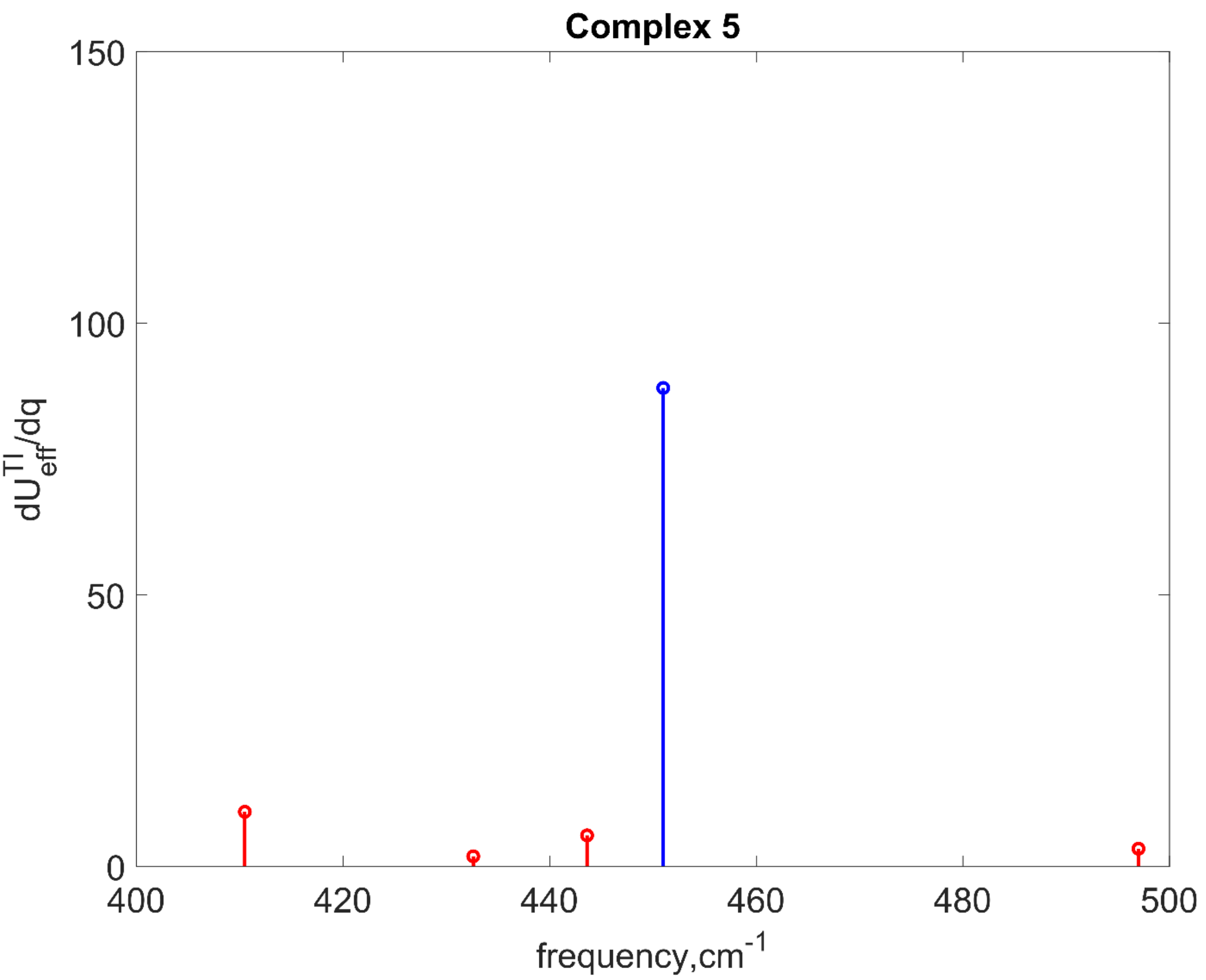

Figure S28. Spin-phonon coupling parameters $\partial U_{eff}^{TI} / \partial q_{\alpha}$ of vibrational frequencies in the vicinity of the transition (in red) from the ground to the lowest excited Kramers doublet (in blue) for complex **5.**

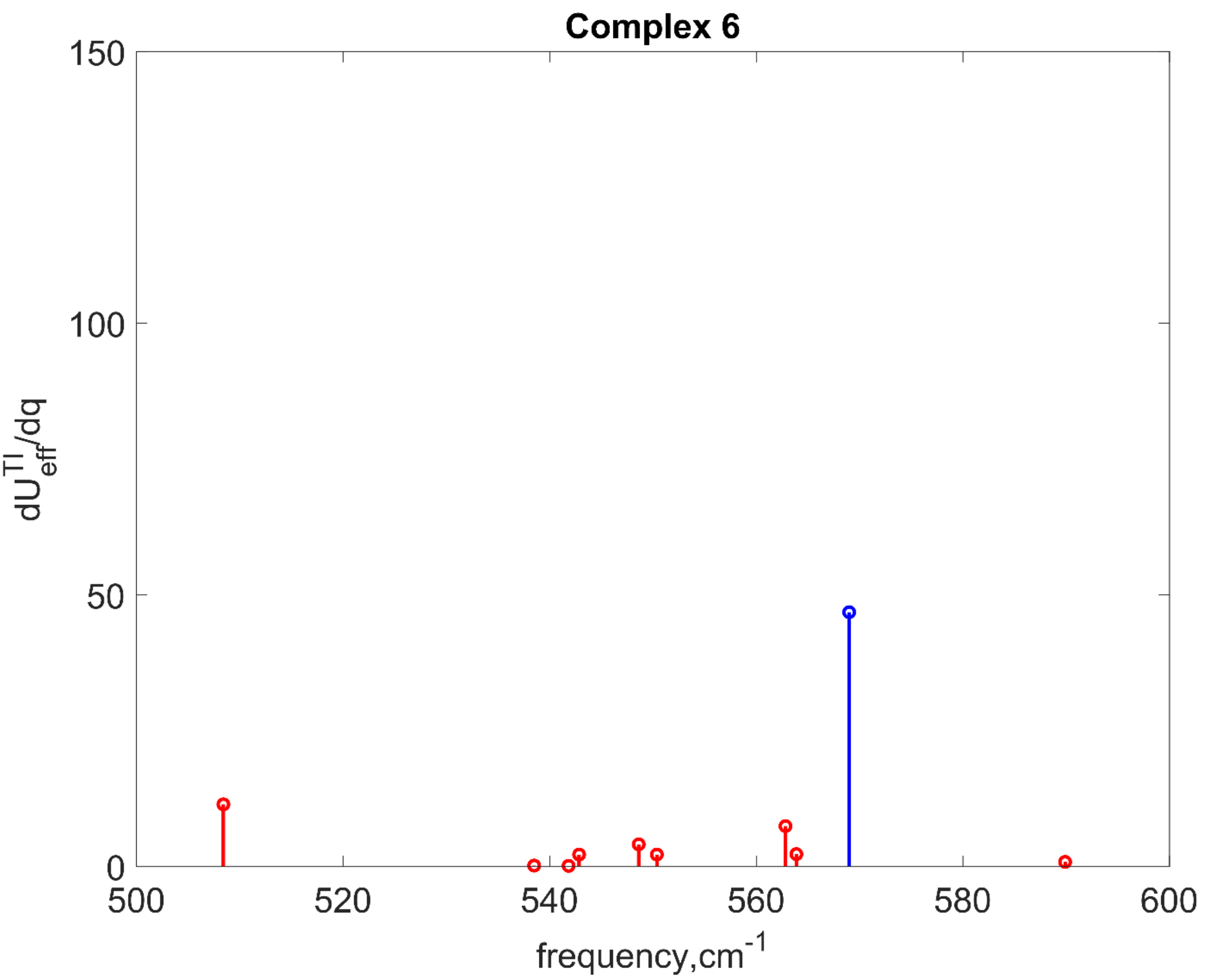

Figure S29. Spin-phonon coupling parameters $\partial U_{eff}^{TI} / \partial q_{\alpha}$ of vibrational frequencies in the vicinity of the transition (in red) from the ground to the lowest excited Kramers doublet (in blue) for complex **6.**

Script 1: A script for U_{eff} calculation

```
format longg
clearvars
clc

mypath = 'PATH';

filename1= 'U.txt';
filename2 ='KD.txt';

path1=[mypath filename1];
path2=[mypath filename2];

output1 = (path1);
output2 = (path2);

E = [KD ENERGIES]; %In cm3
E = 1.4398*E;
ki = [MAGNETIC MOMENT MATRIX ELEMENTS];
T = (1:300);
k = 1.38064852*10^23;

x = size(T,2);
U = zeros(x,1);
kiNm = zeros(x,8);
for A = (1:x);
   T0 = T(A);
e0 = ((-E)/(T0));
e = exp(e0);
```

```
Z = sum(e);
kiT1= (e/Z);
kiT = kiT1.*ki;
N = sum(kiT);
kiN = kiT/N;
U0 = kiN.*E;
U1 = sum(U0)
U(A) = U1;
kiNm(A,:) = kiN(:);
end

plot(T,U)

FIN1 = [T' U];
FIN2 = [T' kiNm]
dlmwrite(output1,FIN1,' ');
dlmwrite(output2,FIN2);
```

Script 2: Python script for extracting ORCA outputs into input files for Script 2 and Script 3

```
import os
Path = os.path.dirname(os.path.abspath("BarOutputs.py"))
lst = os.listdir(Path)
lst.sort()
filelist = (lst)
print (lst)
for i in filelist:
 with open(i) as infile, open(os.path.join(Path, "Bar1.txt"), 'a+') as
outfile:
    copy = False
    for line in infile:
        if line.strip() == "Mult.|      1+     |      1-     |    E (cm-1)
|":
            copy = True
            continue
        elif line.strip() == "Matrix elements of the magnetic moment
connecting Zeeman eigenstates":
            copy = False
            continue
        elif copy:
            outfile.write(line)
for i in filelist:
        with open(i) as infile, open(os.path.join(Path, "Freq.txt"), 'a+')
as outfile:
                copy = False
                for line in infile:
                        if line.strip() == "$vibrational_frequencies":
                                copy = True
                                continue
                        elif line.strip() == "$normal_modes":
                                copy = False
                                continue
                        elif copy:
                                outfile.write(line)
        with open(i) as infile, open(os.path.join(Path, "NModes.txt"),
'a+') as outfile:
                copy = False
                for line in infile:
                        if line.strip() == "$normal_modes":
                                copy = True
                                continue
                        elif line.strip() == "#":
                                copy = False
                                continue
                        elif copy:
                                outfile.write(line)
        with open(i) as infile, open(os.path.join(Path, "Mass.txt"), 'a+')
as outfile:
                copy = False
```

```
                for line in infile:
                        if line.strip() == "$atoms":
                                copy = True
                                continue
                        elif line.strip() == "$actual_temperature":
                                copy = False
                                continue
                        elif copy:
                                outfile.write(line)
for i in filelist:
 with open(i) as infile, open(os.path.join(Path, "U1.txt"), 'a+') as
outfile:
    copy = False
    for line in infile:
        if line.strip() == "check the tunnelling splitting instead":
            copy = True
            continue
        elif line.strip() ==
"###########################################################################
##########################":
            copy = False
            continue
        elif copy:
            outfile.write(line)

fin = open("Bar1.txt", "rt")
fout = open("Bar2.txt", "wt")
for line in fin:
                fout.write(line.replace('|', ' '))
os.remove("Bar1.txt")
fin.close()
fout.close()

fin = open("Bar2.txt", "rt")
fout = open("Bar3.txt", "wt")
for line in fin:
                fout.write(line.replace('---------------', ' '))
os.remove("Bar2.txt")
fin.close()
fout.close()

fin = open("Bar3.txt", "rt")
fout = open("Bar4.txt", "wt")
for line in fin:
                fout.write(line.replace('-------------', ' '))
os.remove("Bar3.txt")
fin.close()
fout.close()

fin = open("Bar4.txt", "rt")
fout = open("Bar.txt", "wt")
for line in fin:
                fout.write(line.replace('------', ' '))
os.remove("Bar4.txt")
```

```
fin.close()
fout.close()

fin = open("U1.txt", "rt")
fout = open("U2.txt", "wt")
for line in fin: fout.write(line[-22:])
os.remove("U1.txt")
fin.close()
fout.close()

fin = open("U2.txt", "rt")
fout = open("U3.txt", "wt")
for line in fin:
                fout.write(line.replace('AVERAGE', ' '))
os.remove("U2.txt")
fin.close()
fout.close()

fin = open("U3.txt", "rt")
fout = open("U4.txt", "wt")
for line in fin:
                fout.write(line.replace('---------------------', ' '))
os.remove("U3.txt")
fin.close()
fout.close()

fin = open("U4.txt", "rt")
fout = open("U5.txt", "wt")
for line in fin:
                fout.write(line.replace('--------------------', ' '))
os.remove("U4.txt")
fin.close()
fout.close()


fin = open("U5.txt", "rt")
fout = open("U.txt", "wt")
for line in fin:
                fout.write(line.replace('|', ' '))
os.remove("U5.txt")
fin.close()
fout.close()
```

Script 3: Matlab script for calculating $\left|\partial U_{eff}^{TI}/\partial q_\alpha\right|$ spin-phonon coupling for individual vibrations

```
clearvars

clc

format longg

mypath='PATH TO FILE'

NAtom = XX; %number of atoms in structure

R = XX; %displacement used in Coords


%Outputs.py requires ORCA output files, in order '001.out', '002.out', ... or '001-0xx.out,

%0xx+1-0yy.out', ..., equilibrium file must be named '000.out'

filename1= 'Bar.txt'; %from Outputs.py script

filename2 ='Freq.txt'; %from Outputs.py script

filename3 = 'Mass.txt'; %from Outputs.py script

filename4 = 'NModes.txt'; %from Outputs.py script

filename5 = 'U.txt'; %from Outputs.py script

filename6 = sprintf('%s','SVCModes_Bar_TI.txt'); %output file for KD Spin-vibration coupling


path1=[mypath filename1];

path2=[mypath filename2];

path3=[mypath filename3];

path4=[mypath filename4];

path5=[mypath filename5];

path6=[mypath filename6];



input = (path1);

input2 = (path2);

input3 = (path3);

input4 = (path4);
```

```
input5 = (path5);

output = (path6)

NCoords = (NAtom*6)+1;
Ninputs = (((NAtom*6)+1)*8)-1;


fid = fopen(input);
Bar0 = dlmread(input);
Bar0 = Bar0(:,4);
Bar = zeros(NCoords,8)
for F1 = (1:numel(Bar)/8);
    F2 = (F1-1)*8;
 Bar(F1,1) = Bar0(F2+1);
 Bar(F1,2) = Bar0(F2+2);
 Bar(F1,3) = Bar0(F2+3);
 Bar(F1,4) = Bar0(F2+4);
 Bar(F1,5) = Bar0(F2+5);
 Bar(F1,6) = Bar0(F2+6);
 Bar(F1,7) = Bar0(F2+7);
 Bar(F1,8) = Bar0(F2+8);
end
Bar(:,1) = 0

InpFreq = dlmread(input2);
InpFreq(1,:) = [];

Freq = InpFreq(:,2);

fid2 = fopen(input3);
```

```
AllMass = textscan(fid2,'%s %f %f %f %f','headerlines',1);
InpMass = cell2mat(AllMass(1,2));
M = InpMass;
A0 = (size(M,1))-1;
M0 = A0*3;
Mass = zeros(1,M0);
for A = (0:A0)
    A1 = (3*A)+1;
    A2 = (3*A)+2;
    A3 = (3*A)+3;
    A4 = A+1;
     Mass(A1) = M(A4);
     Mass(A2) = M(A4);
     Mass(A3) = M(A4);
end
InpNmodes = dlmread(input4,'',1,0);
InpNmodes(:,1) = [];
x2 = size(Freq,1);
x3 = x2+1;
Nmodes0 = zeros(x3,x2);
x4 = size(InpNmodes,1)/x3;

for ZZZ = (1:x4)
    x6 = ((ZZZ-1)*x3)+1;
    x7 = (ZZZ*x3);
    x8 = (x6:x7);
    for ZZY = (1:5)
        x9 = ((ZZZ-1)*5)+ZZY;
        Nmodes0(:,x9) = InpNmodes(x8,ZZY);
    end
end
```

```
Nmodes0(1,:) = [];
NModes = Nmodes0(1:x2,1:x2);

Tun0 = dlmread(input5);
Tun = zeros(NCoords,8)
for F1 = (1:numel(Bar)/8);
    F2 = (F1-1)*8;
 Tun(F1,1) = Tun0(F2+1);
 Tun(F1,2) = Tun0(F2+2);
 Tun(F1,3) = Tun0(F2+3);
 Tun(F1,4) = Tun0(F2+4);
 Tun(F1,5) = Tun0(F2+5);
 Tun(F1,6) = Tun0(F2+6);
 Tun(F1,7) = Tun0(F2+7);
 Tun(F1,8) = Tun0(F2+8);
end

U = zeros(1,NCoords);
for F3 = (1:NCoords);
E = Bar(F3,:);
E = 1.4398*E;
ki = Tun(F3,:);
U2 = zeros(1,1);
kiNm = zeros(1,8);
N = sum(ki);
kiN = ki/N;
U0 = kiN.*E;
U1 = sum(U0);%/(1.4398);
U(F3) = U1;
end
```

```
SPFin = zeros(x2,1);
 for C = (1:x2);
  C1 = 2*C;
  C2 = C1+1;
  for D1 = (1:5)
  y = [U(C1) U(1) U(C2)];
  x = [-R 0 R];
  n = polyfit(x,y,2);
  dn = polyder(n);
  if dn == 0
      dE0 = 0;
  else
  dE0 = dn(1)*0 + dn(2);
  end
  SPFin(C) = abs(dE0);
  end
  end

 Emode = zeros(x2,1);
 for i=1:x2;
  sumE = 0.0;
      for j=1:x2
      sumE=sumE+5.806495*NModes(j,i)*sqrt(1/(Freq(i,1)*Mass(j)))*SPFin(j);
      Emode(i)=sumE;
      end
 end


 FIN = [Freq Emode];

 dlmwrite(output,FIN,'delimiter',' ');
```

Script 4: A Matlab script for generating xyz files with displacement of every atom in Cartesian coordinates

```
%input file - standard xyz file
%Be careful, every running of this script overwrites output file
mypath=‘'; %file location
filename1='Opt.xyz';
filename2='Coords.xyz';
path1=[mypath filename1];
path2=[mypath filename2];
R = 0.05 %displacement size
input = (path1);%input file
output = (path2); %output file - does create if it doesnt exist
fid1 = fopen(input,'r')
startfile = textscan(fid1,'%s %f %f %f','headerlines',2);
numcellsx = startfile(1,2)
numcellsy = startfile(1,3)
numcellsz = startfile(1,4)
Coordsx = cell2mat(numcellsx)
Coordsy = cell2mat(numcellsy)
Coordsz = cell2mat(numcellsz)
Coordsstart = [Coordsx,Coordsy,Coordsz];
stringcells = startfile(1,1)
Elem = stringcells;
fid2 = fopen(output,"w")
x = size(Coordsstart);
x1 = x(1);
x3 = x1*6
Coordsfin = zeros(x1,3,x3);
for A = (1:x1);
for B = (1:6);
N1 = A;
```

```
N2 = B;
if mod(N2,2) == 1;
B1 = (B+1)/2;
Num = Coordsstart(A,B1);
Num2 = Num + R;
Coords2 = Coordsstart;
Coords2(A,B1) = Num2;
N = (6*N1)-(6-N2);
Coordsfin(:,:,N) = Coords2;
else;
B1 = B/2;
Num = Coordsstart(A,B1);
Num2 = Num - R;
Coords2 = Coordsstart;
Coords2(A,B1) = Num2;
N = (6*N1)-(6-N2);
Coordsfin(:,:,N) = Coords2;
end
Coordsfin(:,:,N) = Coords2;
N3 = N1+N2;
if N3 < 3;
fprintf(fid2,'%g\n %s %g\n',x1,'Structure',N);
for C = (1:x1)
w1 =  Elem{1,1}{C,1};
w2 =  Coords2(C,1);
w3 =  Coords2(C,2);
w4 =  Coords2(C,3);
fprintf(fid2,'%s\t %3.7f\t %3.7f\t %3.7f\n',w1,w2,w3,w4);
end
else
fprintf(fid2,'%c\n %g\n %s %g\n','>',x1,'Structure',N);
```

```
for C = (1:x1);
w1 =  Elem{1,1}{C,1};
w2 =  Coords2(C,1);
w3 =  Coords2(C,2);
w4 =  Coords2(C,3);
fprintf(fid2,'%s\t %3.7f\t %3.7f\t %3.7f\n',w1,w2,w3,w4);
end
end
end;
end;
fclose(fid2)
```

Script 5: Matlab script for calculating $|\partial B^l_m/\partial q_\alpha|$ spin-phonon coupling for individual vibrations

```
format long
mypath='C:\Users\kotrka00\Documents\Manuscript\Dycparm 2021\Science\Spin-
phonon script\';
NAtom = 81; %number of atoms in structure
R = 0.05; %displacement used in Coords

%Outputs.py requires ORCA output files, in order '001.out', '002.out', ...
or '001-0xx.out,
%0xx+1-0yy.out', ..., equilibrium file must be named '000.out'
filename1= 'CF.txt'; %from Outputs.py script
filename2 ='Freq.txt'; %from Outputs.py script
filename3 = 'Mass.txt'; %from Outputs.py script
filename4 = 'NModes.txt'; %from Outputs.py script



filename5 = sprintf('%s','SVCModes_CF.txt'); %output file for KD Spin-
vibration coupling

path1=[mypath filename1];
path2=[mypath filename2];
path3=[mypath filename3];
path4=[mypath filename4];
path5=[mypath filename5];

input = (path1);
input2 = (path2);
input3 = (path3);
input4 = (path4);

outputCF = (path5)

NCoords = (NAtom*6)+1;
Ninputs = (((NAtom*6)+1)*8)-1;


fid = fopen(input);
All = dlmread(input);

CFAll = zeros(size(All,3));
for A1 = (1:size(All,1));
    if All(A1,1)== 2;
        CFAll(A1,1) = All(A1,1);
        CFAll(A1,2) = All(A1,2);
        CFAll(A1,3) = All(A1,4);
    else
    end
end

CFAll = CFAll(~all(CFAll == 0, 2),:);

b1 = (0:NCoords-1);
CFm2 = zeros (NCoords,2);
CFm2(:,1) = b1';
```

```
CFm1 = zeros (NCoords,2);
CFm1(:,1) = b1';

CF0 = zeros (NCoords,2);
CF0(:,1) = b1';

CFp1 = zeros (NCoords,2);
CFp1(:,1) = b1';

CFp2 = zeros (NCoords,2);
CFp2(:,1) = b1';
b2 = int32(5)

for A2 = (1:size(CFAll,1));
    if CFAll(A2,2) == -2;
        CFm2(idivide(A2,b2,'ceil'),2) = CFAll(A2,3);
    elseif CFAll(A2,2) == -1;
        CFm1(idivide(A2,b2,'ceil'),2) = CFAll(A2,3);
    elseif CFAll(A2,2) == 0;
        CF0(idivide(A2,b2,'ceil'),2) = CFAll(A2,3);
    elseif CFAll(A2,2) == 1;
        CFp1(idivide(A2,b2,'ceil'),2) = CFAll(A2,3);
    else
        CFp2(idivide(A2,b2,'ceil'),2) = CFAll(A2,3);
    end
end

InpFreq = dlmread(input2);
InpFreq(1,:) = [];

Freq = InpFreq(:,2);

fid2 = fopen(input3);
AllMass = textscan(fid2,'%s %f %f %f %f','headerlines',1);
InpMass = cell2mat(AllMass(1,2));
M = InpMass;
A0 = (size(M,1))-1;
M0 = A0*3;
Mass = zeros(1,M0);
for A = (0:A0)
    A1 = (3*A)+1;
    A2 = (3*A)+2;
    A3 = (3*A)+3;
    A4 = A+1;
     Mass(A1) = M(A4);
     Mass(A2) = M(A4);
     Mass(A3) = M(A4);
end
InpNmodes = dlmread(input4,'',1,0);
InpNmodes(:,1) = [];
x2 = size(Freq,1);
x3 = x2+1;
Nmodes0 = zeros(x3,x2);
x4 = size(InpNmodes,1)/x3;

for ZZZ = (1:x4)
    x6 = ((ZZZ-1)*x3)+1;
    x7 = (ZZZ*x3);
    x8 = (x6:x7);
    for ZZY = (1:5)
```

```
        x9 = ((ZZZ-1)*5)+ZZY;
        Nmodes0(:,x9) = InpNmodes(x8,ZZY);
    end
end
Nmodes0(1,:) = [];
NModes = Nmodes0(1:x2,1:x2);

CF = [CFm2(:,2) CFm1(:,2) CF0(:,2) CFp1(:,2) CFp2(:,2)];

for C = (1:A3)
 C1 = 2*C;
 C2 = C1+1;
 for D1 = (1:5)
 y = [CF(C1,D1) CF(1,D1) CF(C2,D1)];
 x = [-R 0 R];
 n = polyfit(x,y,2);
 dn = polyder(n);
 if dn == 0
     dE0 = 0;
 else
 dE0 = dn(1)*0 + dn(2);
 end
 SPFin(C,D1) = abs(dE0);
 end
 end

Emode = zeros(x2,5);
for A7 = (1:5)
for i=1:x2;
 sumE = 0.0;
     for j=1:x2

sumE=sumE+5.806495*NModes(j,i)*sqrt(1/(Freq(i,1)*Mass(j)))*SPFin(j,A7);
     Emode(i,A7)=sumE;
     end
end
end

FIN = [Freq abs(Emode)];
dlmwrite(outputCF,FIN,'delimiter',' ');
```
