# Supplementary figures and images for: Theoretical Magnetic Relaxation and Spin–Phonon Coupling Study in a Series of Molecular Engineering Designed Bridged Dysprosocenium Analogues
Source: Inorg Chem. 2023 Oct 9;62(42):17499–509. doi: 10.1021/acs.inorgchem.3c02916 (PMC10598879; doi:10.1021/acs.inorgchem.3c02916)

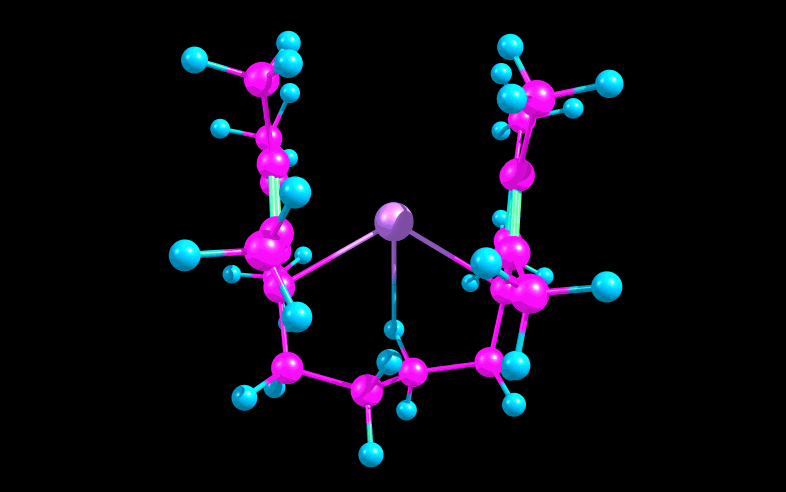

Supplement: Supplementary file 3 — ic3c02916_si_003.zip [file ic3c02916_si_003.zip › vibrations/comp1vibr83.gif]

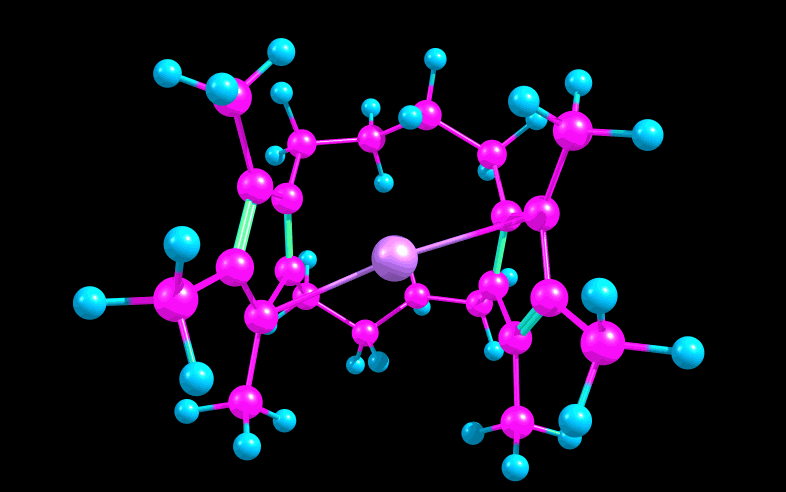

Supplement: Supplementary file 3 — ic3c02916_si_003.zip [file ic3c02916_si_003.zip › vibrations/comp2avibr22.gif]

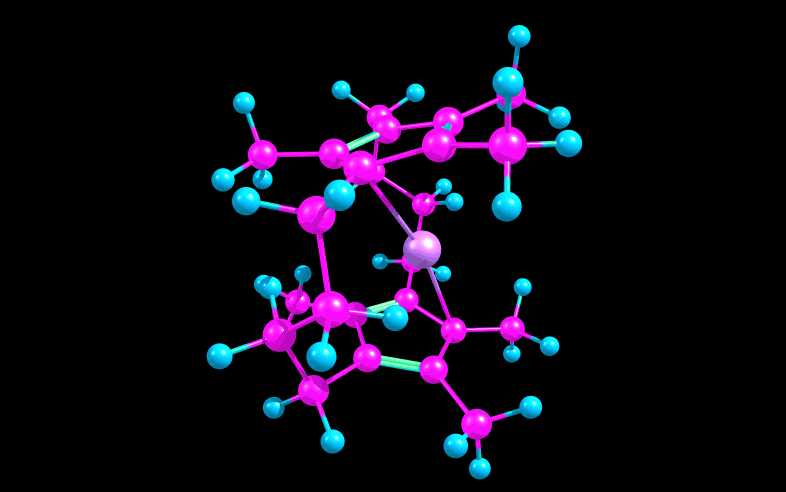

Supplement: Supplementary file 3 — ic3c02916_si_003.zip [file ic3c02916_si_003.zip › vibrations/comp2bvibr107.gif]

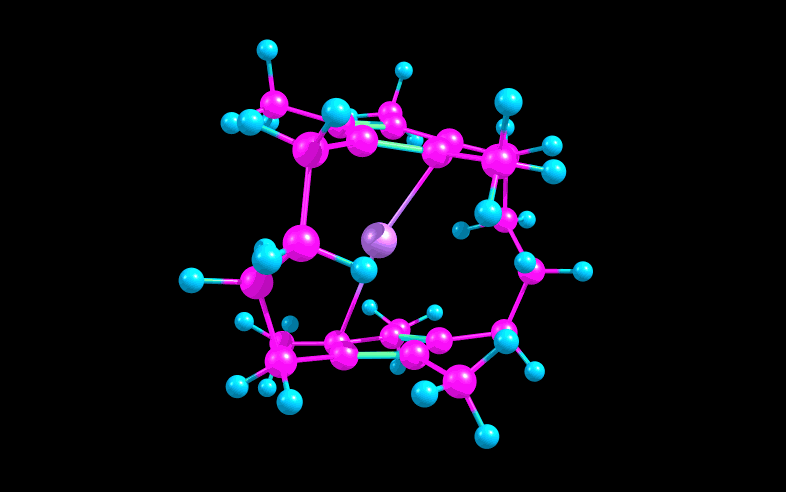

Supplement: Supplementary file 3 — ic3c02916_si_003.zip [file ic3c02916_si_003.zip › vibrations/comp2bvibr112.gif]

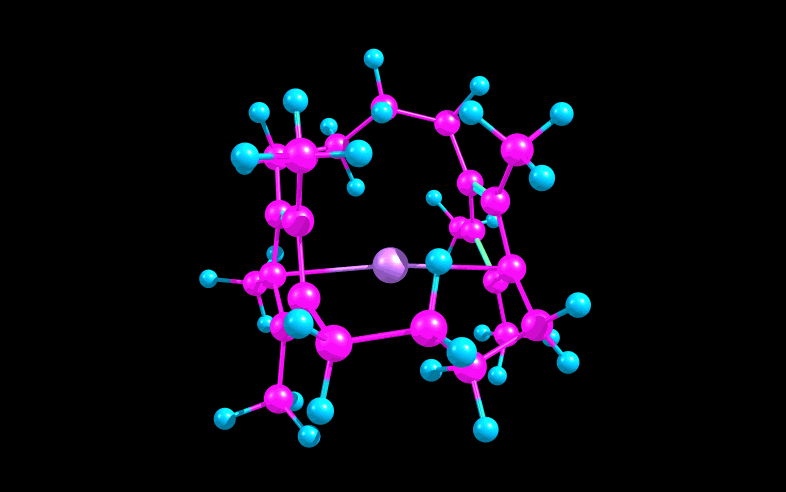

Supplement: Supplementary file 3 — ic3c02916_si_003.zip [file ic3c02916_si_003.zip › vibrations/comp2bvibr208.gif]

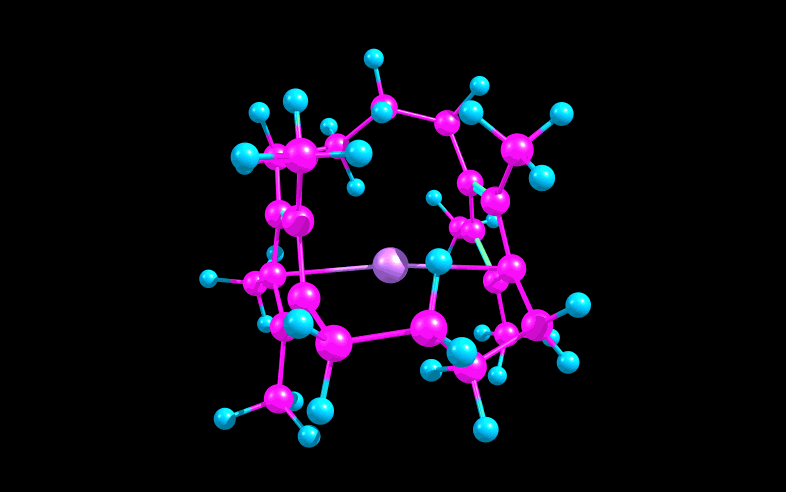

Supplement: Supplementary file 3 — ic3c02916_si_003.zip [file ic3c02916_si_003.zip › vibrations/comp2bvibr44.gif]

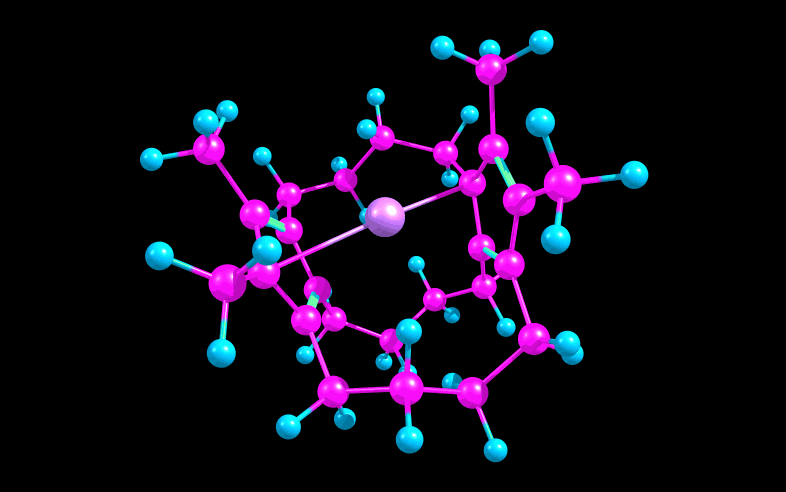

Supplement: Supplementary file 3 — ic3c02916_si_003.zip [file ic3c02916_si_003.zip › vibrations/comp3avibr103.gif]

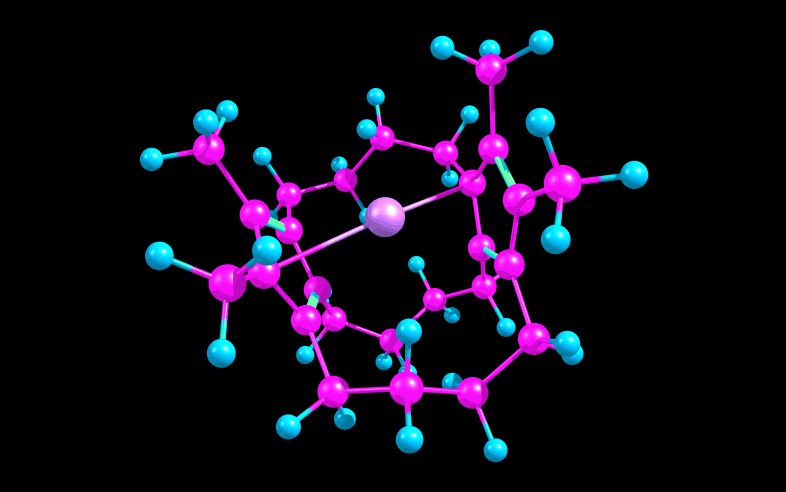

Supplement: Supplementary file 3 — ic3c02916_si_003.zip [file ic3c02916_si_003.zip › vibrations/comp3avibr104.gif]

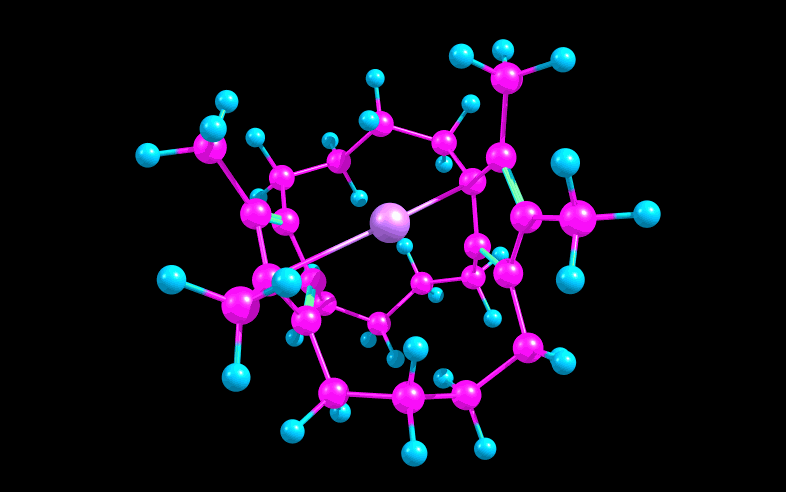

Supplement: Supplementary file 3 — ic3c02916_si_003.zip [file ic3c02916_si_003.zip › vibrations/comp3avibr71.gif]

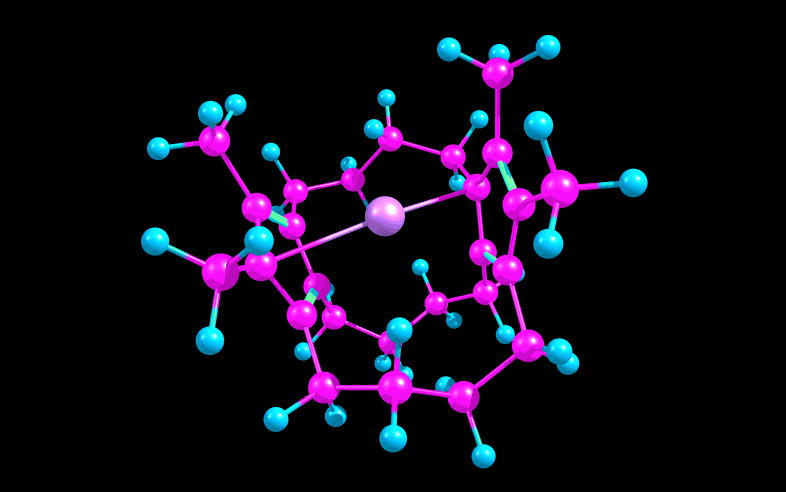

Supplement: Supplementary file 3 — ic3c02916_si_003.zip [file ic3c02916_si_003.zip › vibrations/comp3avibr85.gif]

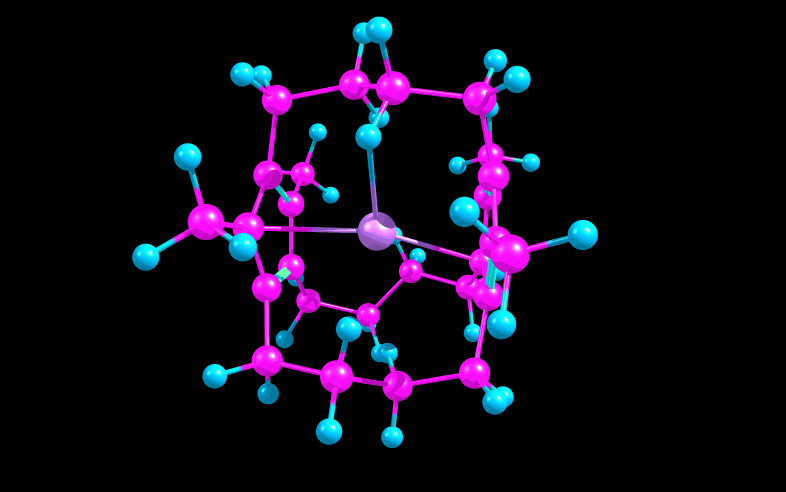

Supplement: Supplementary file 3 — ic3c02916_si_003.zip [file ic3c02916_si_003.zip › vibrations/comp3bvibr224.gif]

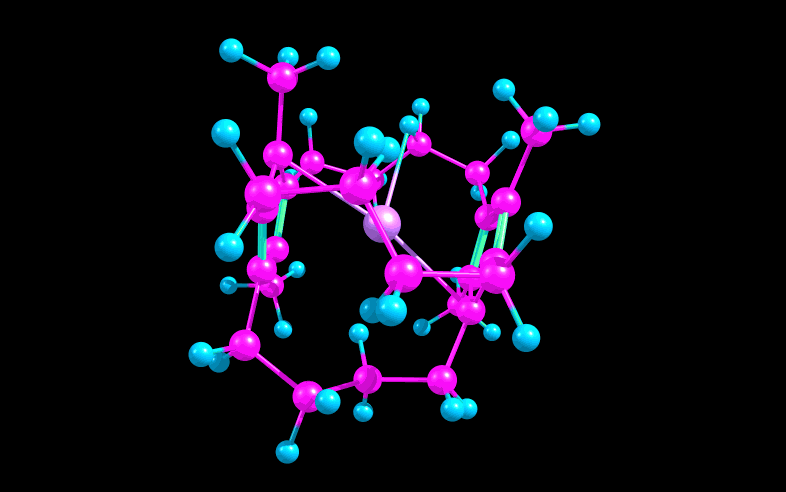

Supplement: Supplementary file 3 — ic3c02916_si_003.zip [file ic3c02916_si_003.zip › vibrations/comp3bvibr33.gif]

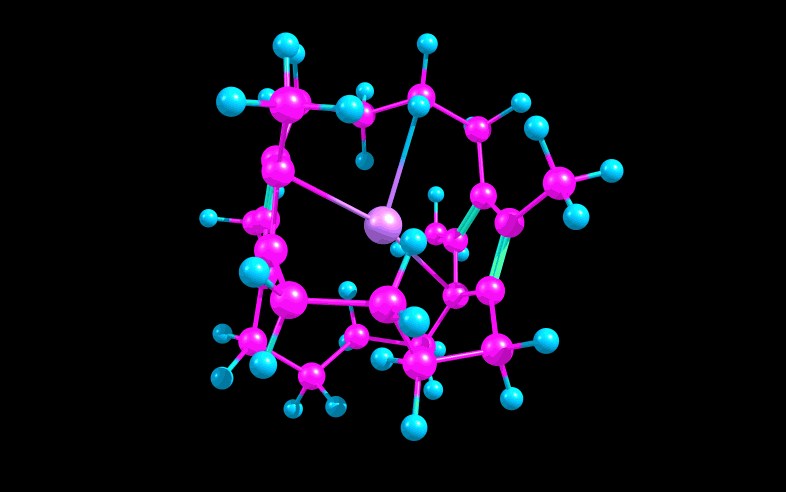

Supplement: Supplementary file 3 — ic3c02916_si_003.zip [file ic3c02916_si_003.zip › vibrations/comp3bvibr43.gif]

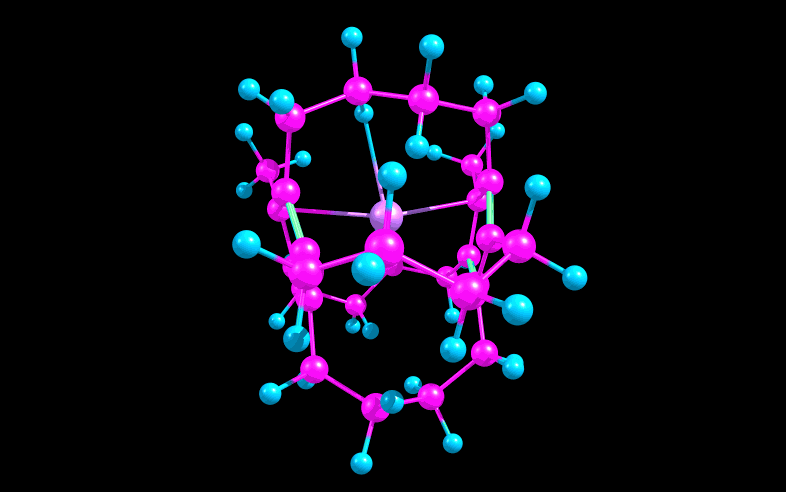

Supplement: Supplementary file 3 — ic3c02916_si_003.zip [file ic3c02916_si_003.zip › vibrations/comp4vibr170.gif]

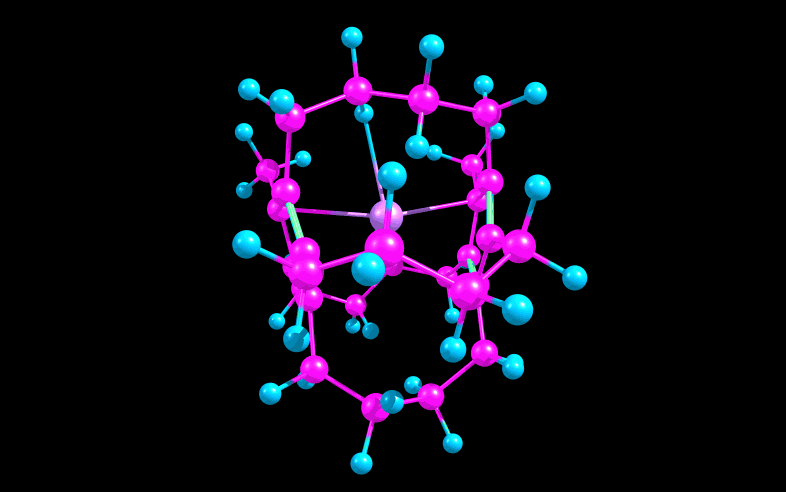

Supplement: Supplementary file 3 — ic3c02916_si_003.zip [file ic3c02916_si_003.zip › vibrations/comp4vibr199.gif]

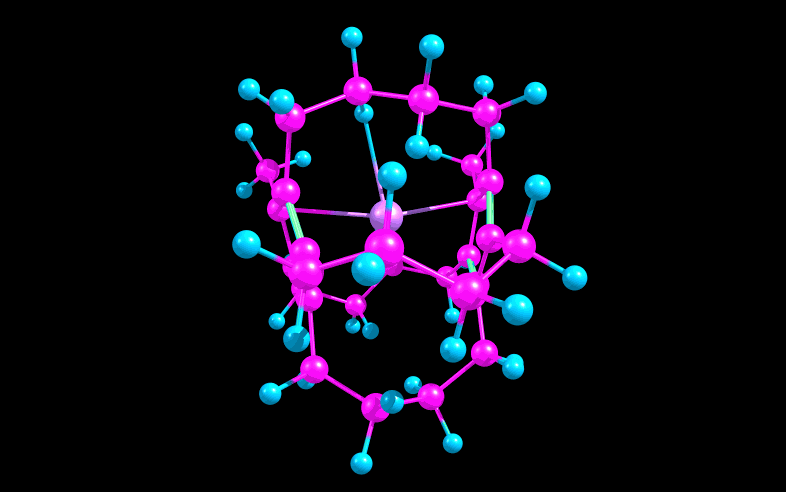

Supplement: Supplementary file 3 — ic3c02916_si_003.zip [file ic3c02916_si_003.zip › vibrations/comp4vibr34.gif]

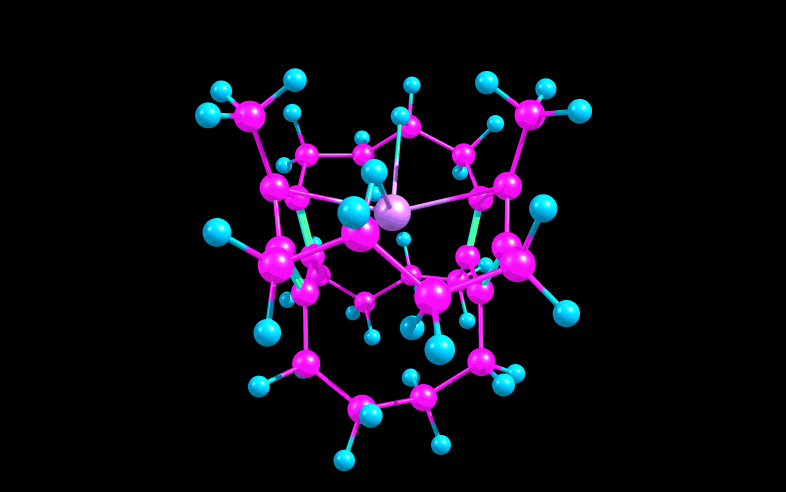

Supplement: Supplementary file 3 — ic3c02916_si_003.zip [file ic3c02916_si_003.zip › vibrations/comp4vibr93.gif]

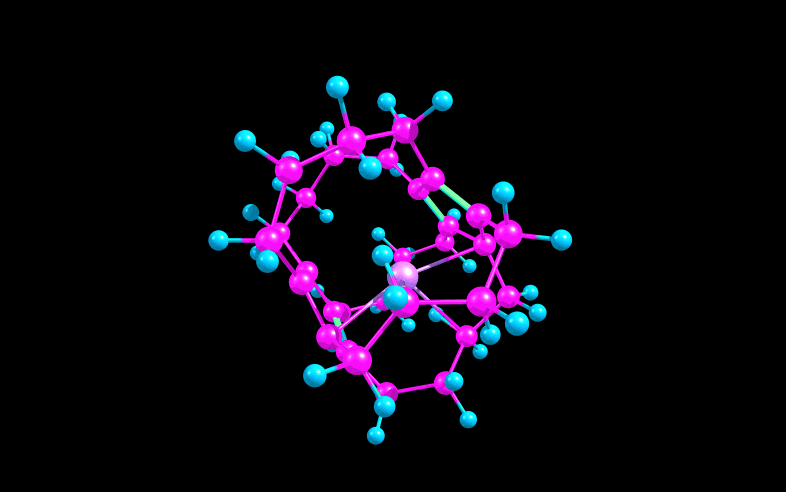

Supplement: Supplementary file 3 — ic3c02916_si_003.zip [file ic3c02916_si_003.zip › vibrations/comp5vibr103.gif]

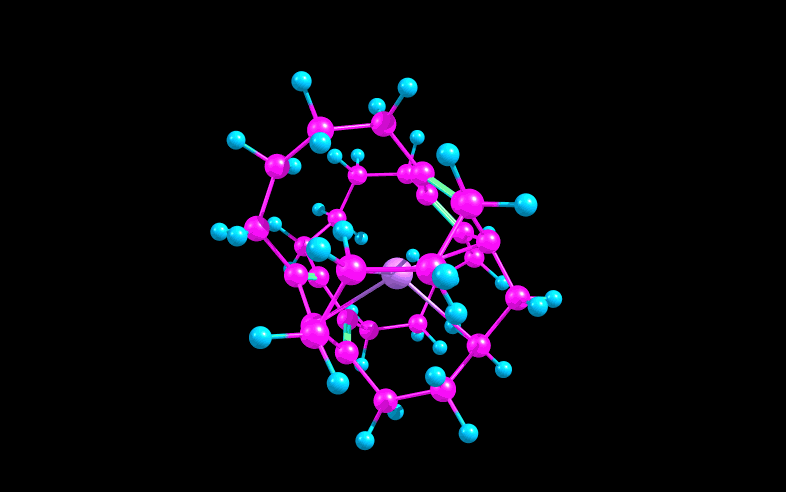

Supplement: Supplementary file 3 — ic3c02916_si_003.zip [file ic3c02916_si_003.zip › vibrations/comp5vibr190.gif]

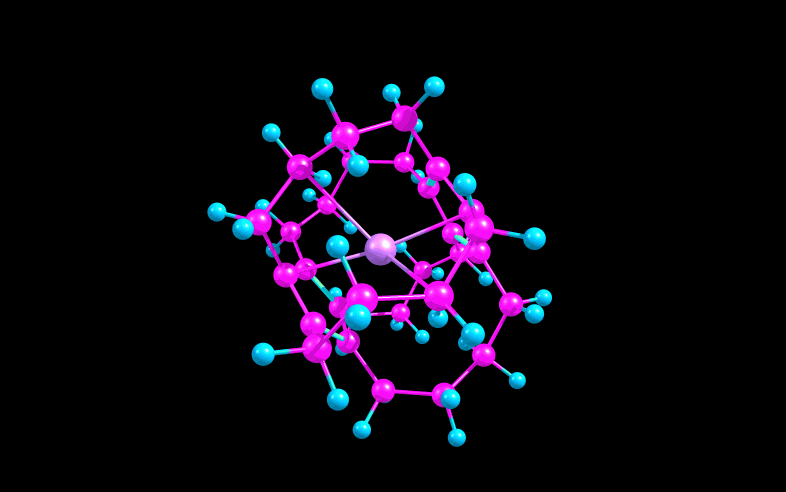

Supplement: Supplementary file 3 — ic3c02916_si_003.zip [file ic3c02916_si_003.zip › vibrations/comp5vibr72.gif]

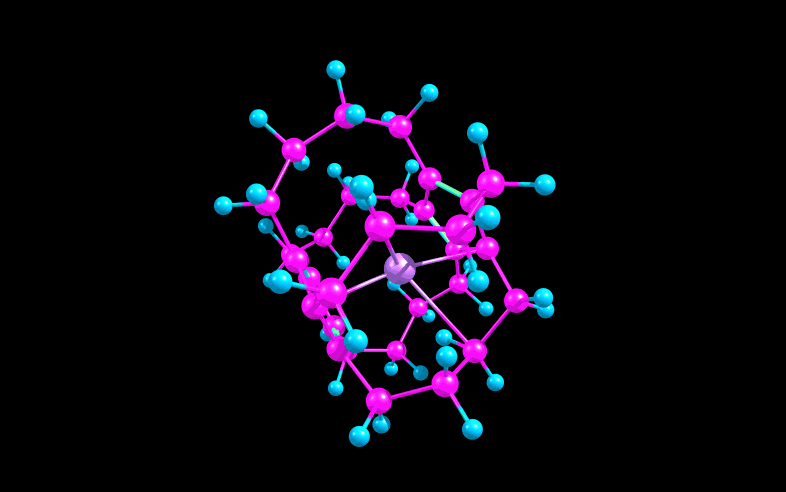

Supplement: Supplementary file 3 — ic3c02916_si_003.zip [file ic3c02916_si_003.zip › vibrations/comp5vibr93.gif]
